# Quantitative RNAseq analysis of Ugandan KS tumors reveals KSHV gene expression dominated by transcription from the LTd downstream latency promoter

**DOI:** 10.1371/journal.ppat.1007441

**Published:** 2018-12-17

**Authors:** Timothy M. Rose, A. Gregory Bruce, Serge Barcy, Matt Fitzgibbon, Lisa R. Matsumoto, Minako Ikoma, Corey Casper, Jackson Orem, Warren Phipps

**Affiliations:** 1 Department of Pediatrics, University of Washington, Seattle, Washington, United States of America; 2 Center for Global Infectious Disease Research, Seattle Children’s Research Institute, Seattle, Washington, United States of America; 3 Vaccine and Infectious Disease Division, Fred Hutchinson Cancer Research Center, Seattle, Washington, United States of America; 4 Department of Medicine, University of Washington, Seattle, Washington, United States of America; 5 Department of Global Health, University of Washington, Seattle, Washington, United States of America; 6 Uganda Cancer Institute, Kampala, Uganda; Hannover Medical School, GERMANY

## Abstract

KSHV is endemic in Uganda and the HIV epidemic has dramatically increased the incidence of Kaposi sarcoma (KS). To investigate the role of KSHV in the development of KS, we obtained KS biopsies from ART-naïve, HIV-positive individuals in Uganda and analyzed the tumors using RNAseq to globally characterize the KSHV transcriptome. Phylogenetic analysis of ORF75 sequences from 23 tumors revealed 6 distinct genetic clusters with KSHV strains exhibiting M, N or P alleles. RNA reads mapping to specific unique coding sequence (UCDS) features were quantitated using a gene feature file previously developed to globally analyze and quantitate KSHV transcription in infected endothelial cells. A pattern of high level expression was detected in the KSHV latency region that was common to all KS tumors. The clear majority of transcription was derived from the downstream latency transcript promoter P3(LTd) flanking ORF72, with little evidence of transcription from the P1(LTc) latency promoter, which is constitutive in KSHV-infected lymphomas and tissue-culture cells. RNAseq data provided evidence of alternate P3(LTd) transcript editing, splicing and termination resulting in multiple gene products, with 90% of the P3(LTd) transcripts spliced to release the intronic source of the microRNAs K1-9 and 11. The spliced transcripts encode a regulatory uORF upstream of Kaposin A with alterations in intervening repeat sequences yielding novel or deleted Kaposin B/C-like sequences. Hierarchical clustering and PCA analysis of KSHV transcripts revealed three clusters of tumors with different latent and lytic gene expression profiles. Paradoxically, tumors with a latent phenotype had high levels of total KSHV transcription, while tumors with a lytic phenotype had low levels of total KSHV transcription. Morphologically distinct KS tumors from the same individual showed similar KSHV gene expression profiles suggesting that the tumor microenvironment and host response play important roles in the activation level of KSHV within the infected tumor cells.

## Author summary

Kaposi’s sarcoma (KS) is among the world’s most common AIDS-associated malignancies. The Kaposi sarcoma-associated herpesvirus (KSHV) was first identified in KS tumors and is now known to be the causative agent of all forms of KS, including classical, endemic, iatrogenic and HIV-associated. KSHV is endemic to sub-Saharan Africa with high infection rates in children and adults. Compounded with the high rate of HIV and AIDS in this area, pediatric and adult KS are some of the most common malignancies with the highest fatality rates. We used RNA deep sequencing to characterize KSHV expression in a large collection of KS biopsies from HIV-infected Ugandans. Using a novel approach to quantitate expression in complex genomes like KSHV, we found that RNA from a single KSHV promoter within the latency region constituted the majority of KSHV transcripts in the KS tumors. Alternate RNA processing produced different spliced and un-spliced transcripts with different coding potentials. Differential expression of other KSHV genes was detected which segregated the tumors into three different types depending on their expression of lytic or latency genes. Quantitative analysis of KSHV expression in KS tumors provides an important basis for future studies on the role of KSHV in the development of KS.

## Introduction

Since its discovery in 1994, the Kaposi sarcoma-associated herpesvirus (KSHV), also known as human herpesvirus-8 (HHV-8), has been identified as the etiologic cause of all types of Kaposi sarcoma (KS), and is etiologically associated with primary effusion lymphoma (PEL) and multicentric Castleman Disease (MCD)[1]. The KSHV genome encodes more than 90 genes, including a core of genes highly conserved among the different herpesviruses [2]. In addition, a number of novel genes exhibiting sequence homology to cellular genes implicated in mitosis, cell cycle regulation and immunity have been identified [3].

In vitro, KSHV infects a variety of cell types including endothelial, epithelial, fibroblast and lymphocyte lineages [4] and establishes a latent infection in which only a subset of genes are detected, including LANA (ORF73)—the latency-associated nuclear antigen, vCYC (ORF72)–a cyclin D homolog, and vFLIP (ORF71)–a homolog of the Fas-associated protein with death domain-like interleukin 1beta-converting enzyme/caspase-8-inhibitory protein [5–7]. Early studies with cultured PEL cells determined that these genes were present on a tricistronic mRNA originating from the constitutive latency transcript (LTc) promoter upstream of ORF73 [6, 8]. Latent KSHV infections, first characterized in endothelial cells in vitro, resulted in the majority of cells expressing LANA as punctate dots in the nucleus, with a small population of cells (~1%) expressing ORF59, a DNA polymerase processivity factor [9–11]. In most cell types, the widespread lytic reactivation necessary for production of infectious virus was achieved only by using chemical inducers such as the phorbol ester TPA or the HDAC inhibitor sodium butyrate or by overexpression of exogenous recombinant ORF50, the replication transactivator (RTA) [4, 11, 12]. The complement of KSHV genes has been divided into functional groups based on their initial expression during establishment of viral latency and their response to artificial induction, with latency, immediate-early, early and late gene designations. While ORF50 RTA and ORF59 represent immediate-early and early genes, respectively, the genes encoding the major capsid protein (MCP; ORF25), and the virion envelope glycoproteins, gB(ORF8) and K8.1 are examples of KSHV late genes.

Early attempts to determine the expression profile of KSHV in KS tumors examined the RNA transcripts in KS lesions by Northern analysis. Two small RNA transcripts were detected, including T0.7 encoding the K12 Kaposin membrane-associated protein and T1.1, a polyadenylated nuclear RNA (PAN) [13]. Using in situ hybridization, the T0.7 RNA was detected in all KS tumor cells, while the T1.1 RNA was present in only 10% of the T0.7 positive cells [14]. In addition, RNA encoding the MCP late gene was detected in the same cells containing the T1.1 transcript. Once antibodies were available, the expression and localization of viral proteins was examined by immunohistochemical methods. The major latency-associated protein LANA was consistently detected in the nuclei of the vast majority of spindeloid tumor cells in the KS lesion [5, 15, 16]. In contrast, markers of lytic replication, including ORF50 RTA, ORF59, and the vIL-6 homolog K2 were detected very rarely (<1%) in the tumor cells, while no expression of late genes, including ORF26 and ORFK8.1 was observed [17–22]. Using an array of real-time PCR assays targeting the majority of known KSHV genes, the expression of mRNA transcripts from the latency locus, including ORFs 71 (vFLIP), 72 (vCYC) and 73 (LANA), was detected in 21 KS biopsies [23]. Subsequently, using the same PCR array, two types of transcriptional signatures were detected in a panel of KS tumors [24]. In half of these tumors, KSHV transcription was limited to the latency-associated genes. In the other half of the KS tumors, variable and incomplete expression of viral lytic mRNAs was observed.

We have utilized RNA deep sequencing (RNAseq) to globally examine the KSHV transcriptome in latently-infected tissue culture cells in vitro [25]. Due to the highly complex nature of the KSHV transcriptome, we developed a novel approach to more accurately quantitate specific viral transcripts using unique coding sequence (UCDS) features targeting non-overlapping regions of KSHV transcripts. We sequenced the RNA transcripts from in vitro infected cells to a great depth, with more than a million reads mapping to the KSHV genome. High levels of transcripts were observed across the complete KSHV genome in the absence of artificial induction with chemicals or recombinant proteins. This allowed us to develop a detailed map of KSHV transcription, which informed the development of the UCDS features in a new gene feature file (KSHV NC_009333 UCDS ver 020116.GFF) to globally analyze and quantitate KSHV gene expression [25].

Recently, RNAseq has been used to characterize the viral and cellular transcriptome in KS tumor and non-cancer biopsies of African epidemic HIV+ KS patients undergoing anti-retroviral therapy (ART) [26]. Lesions from four individuals were analyzed yielding 718–17,202 reads that mapped to known KSHV ORFs in the NC_009333 KSHV reference genome. High level expression of the latency region was reported but no obvious pattern was observed between the tumor biopsies. In the current study, we have used RNAseq to analyze and quantitate KSHV gene expression in a large collection of 41 KS tumor biopsies from HIV-infected individuals in Uganda who were naïve to ART. The RNAseq libraries were sequenced to an average depth of 100 million reads yielding up to 159,000 KSHV-mapped reads. Using the new gene feature file, we quantitated the RNA reads mapping to non-overlapping UCDS features and identified a set of transcripts from the latency region that was highly and consistently expressed in all the KS tumors.

## Results

### Participant and KS sample characteristics

**Fig 1:**
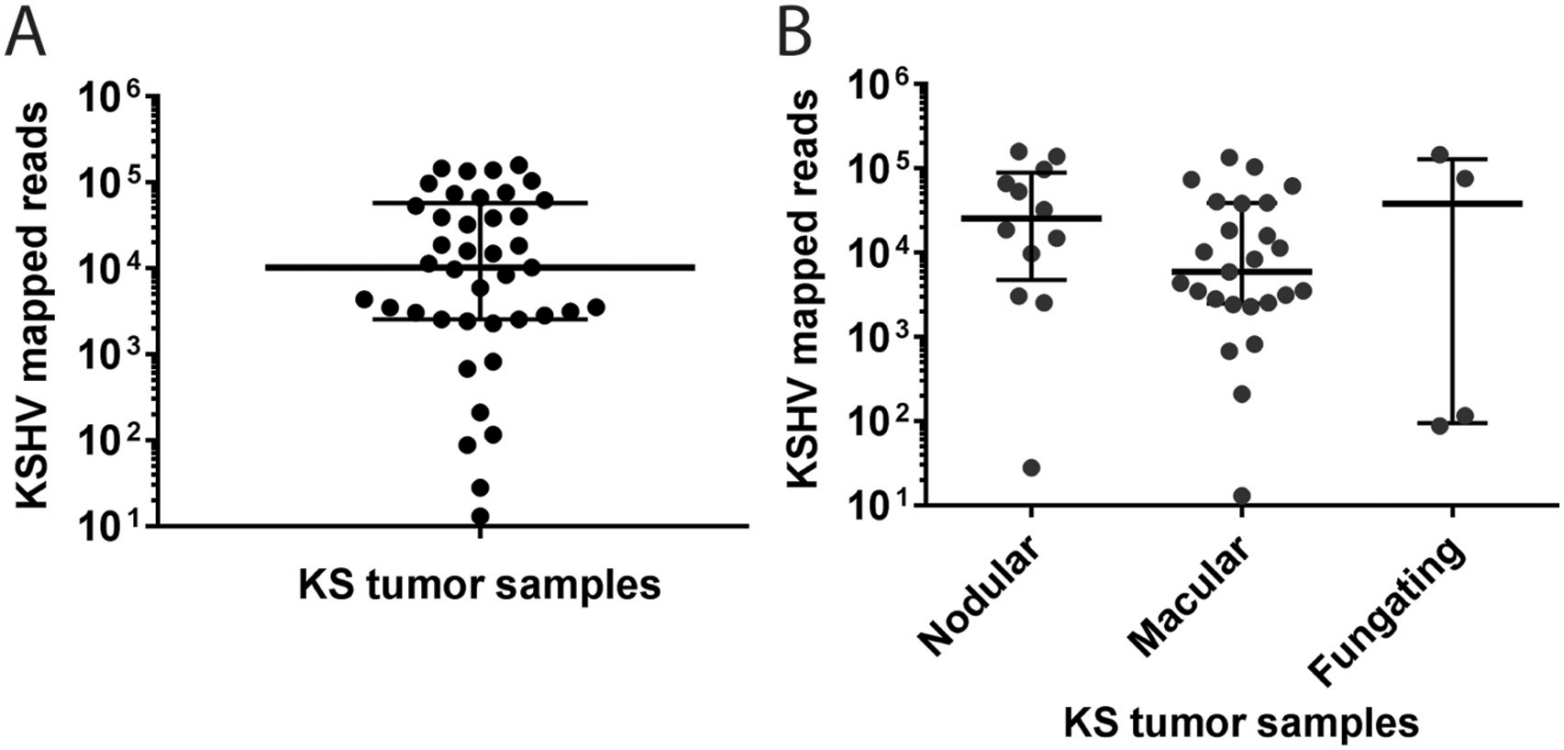
Comparison of reads mapping to the KSHV genome in the RNAseq libraries from different Ugandan KS tumor biopsies. A) RNA reads from the RNAseq libraries prepared from 41 KS biopsies (S1 Table) were initially mapped to the human genome (hg19) using TopHat2. The set of reads not matching with the human genome were further mapped to the KSHV reference sequence NC_009333 for the KSHV GK18 strain and the reads from the different KS biopsies were compared. B) The RNA read data were grouped according to the tumor morphology classification.

Thirty Ugandan participants contributed 41 cutaneous KS tumors for RNA-Seq analysis, with 11 participants contributing 2 samples (S1 Table). The majority of participants were men (21/26; 80%) with a median age of 34 years (range, 23–56 years) (Table 1). At the time of KS diagnosis, all had advanced T1 tumor stage per AIDS Clinical Trials Group staging criteria, which includes those with extensive oral KS, visceral KS or tumor-associated edema [27]. All patients were naïve to antiretroviral treatment. Median CD4 T-cell count was 183 cells/mm^3^ (IQR, 58,331 cells/mm^3^) and median HIV plasma RNA was 5.5 log10 copies/mL (IQR, 5.1, 5.6 log10 copies/mL). Tumor samples represented a range of morphotypes, including 24 macular (58%), 13 nodular (32%), and 4 fungating (10%) lesions. Total nucleic acids were extracted from the 41 KS biopsies and cDNA libraries were prepared from poly-A-selected RNA and subjected to RNA deep-sequencing on the Illumina platform (S1 Table). 37 independent KS samples were sequenced for 50 bp from paired-end non-stranded libraries. Four additional KS samples were sequenced from stranded libraries to distinguish sense and anti-sense RNA transcripts. Total reads ranged from 81–124 million for the paired-end libraries and 35–52 million for the stranded libraries, which were analyzed at a lower depth of sequencing. RNA reads mapping to the human genome HG19 were subtracted from the libraries and the remaining reads were mapped onto the KSHV NCBI reference sequence NC_009333, strain GK18, with mapped KSHV reads ranging from 13 to 158,924, with a median of 10,232 (Fig 1A). Five of the KS biopsies had greater than 100,000 KSHV-mapped reads. While seven of the KS tumors had less than 1,000 total KSHV-mapped reads, the level of reads mapping to the human genome in these samples was comparable to the other KS samples (S1 Table). A comparison of KSHV mRNA expression in the different tumor morphotypes showed no significant differences in total KSHV-mapped reads (Fig 1B). The KSHV genome copy number per cell was determined for four of the KS samples (001_C, 006_B, 026_B, and 029_B). A comparison of the Ct values obtained from qPCR assays targeting KSHV and the cellular gene oncostatin M, essentially as described in [28], revealed similar KSHV genome copy numbers ranging from 0.5–0.9 KSHV genomes/cell (mean = 0.6). A comparison of the number of mapped KSHV reads/KSHV genome copies showed a variance of 8.6% across the four KS biopsies.

**Table 1:** Participant Demographic and Clinical Characteristics.

Characteristics	Cohort (N = 30)
**Gender**	
Men	25 (83%)
Women	5 (17%)
**Age**	
(median, range)	34 yr; 18–60
**Tumor Stage**	
T1	30 (100%)
**Baseline CD4 T-cell count**	
(median, IQR)	183 (55, 288)
**Baseline HIV plasma RNA, log 10**	
(median, IQR)	5.5 (5.1, 6.0)
**Tumor morphotype**	**Samples (N = 41)**
Macular	25 (61%)
Nodular	12 (29%)
Fungating	4 (10%)

### Visualization of KSHV gene expression in KS tumors

**Fig 2:**
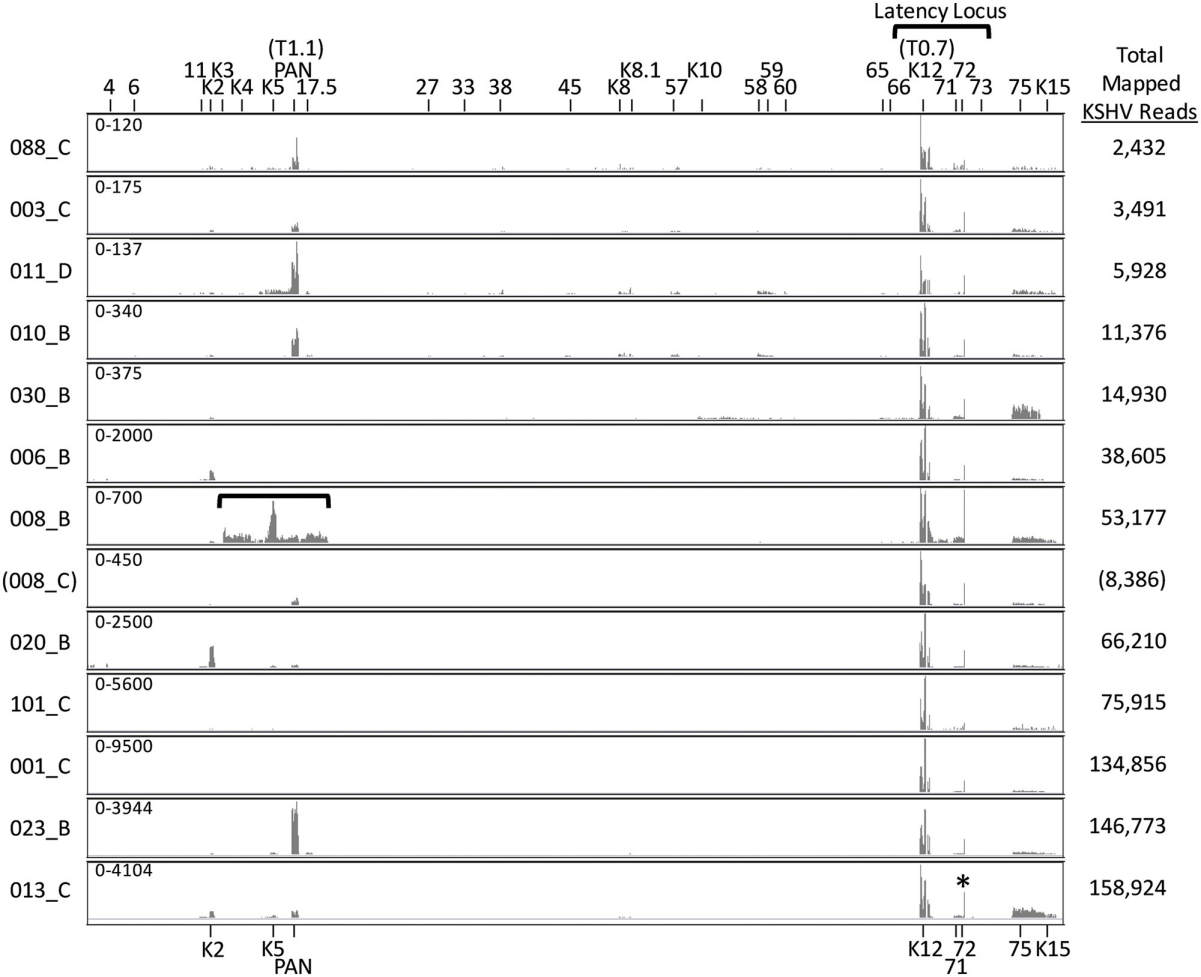
KSHV transcriptome analysis in Ugandan KS tumor biopsies—Linear scale. RNA-seq analysis was performed on 41 KS tumor biopsies and the RNA reads were aligned to the complete reference genomic sequence of KSHV, strain GK18, (NC_009333) and visualized using IGV from the left to the right end of the genome. The vertical axis represents the number of reads aligned to each nucleotide position of the KSHV reference genome (autoscaled) and the visible axis range is shown on the left. Data from 13 representative biopsies from 12 patients are shown in ascending order based on total KSHV mapped read levels. The positions of various KSHV genes across the KSHV genome are indicated at the top and highly expressed genes are indicated at the bottom. An asterisk in the bottom panel indicates the position of a highly expressed region of the KSHV genome flanking the ORF72 coding sequence (P3-exon1). Two different KS biopsies from the same individual (008) are shown. KS biopsy 008_C shows a transcript pattern that is similar to the other KS biopsies, while biopsy 008_B shows high level expression of a region of the left end of the KSHV genome flanking the region encoding PAN (brackets). As discussed in the text, the KSHV strain in KS biopsy 030_C contains the M-allele of K15 and no reads mapped to the P-allele of K15 present in the GK18 reference sequence used for mapping (B: 030_C lane, arrow).

The RNA-seq reads mapping to the complete KSHV genome were visualized with the Integrated Genome Viewer (IGV), using a linear scale to provide an overview of the highly expressed regions of the genome. Representative data from KS tumors with 2,432 to 158,924 total KSHV-mapped reads are shown (Fig 2), while data from the seven KS tumors with total KSHV-mapped reads less than 1,000 are provided in S1 Fig The highest level of RNA reads mapped to the region of the T0.7 RNA transcript within the latency locus at the right end of the KSHV genome (Fig 2). T0.7 encodes ORF K12 Kaposin A, a small transmembrane protein implicated in cell adhesion and transformation [29, 30]. High levels of reads mapping to the T0.7/K12 region were consistently detected in all the KS tumor samples, regardless of the level of total KSHV-mapped reads in each sample. High levels of RNA reads also mapped to a small region of the KSHV genome located immediately to the right of ORF72, observable as a single vertical line of mapped reads (Fig 2; indicated with an asterisk in the bottom graphic). A lower but consistent level of reads mapped to the adjacent region containing ORFs 71 and 72. ORFs 71 and 72 encode vFLIP, which functions to promote cell survival and inhibit KSHV lytic replication, and vCYC, which regulates cell-cycle progression, respectively [31]. Downstream of the latency locus at the right end of the genome, a moderate level of reads mapped consistently to the region encoding ORF75, a large tegument protein essential for viral lytic replication [32], and K15, a multiple-pass membrane protein that modulates cellular signaling pathways associated with KSHV-induced angiogenesis [33–35](Fig 2). At the left end of the genome, a moderate level of reads mapped inconsistently to the multifunctional regulatory polyadenylated nuclear (PAN; T1.1) RNA transcript, with higher levels in the tumors with lower total KSHV-mapped reads shown in the upper part of Fig 2. One fungating tumor 023_B showed high levels of both PAN and total KSHV-mapped reads. Some tumor samples contained moderate levels of reads mapping to ORFK2, the viral interleukin-6 (vIL-6) homolog, or ORFK5, the ubiquitin ligase modulator of immune response (MIR2). One tumor, 008_B, showed high level expression of a sharply delineated region of the KSHV genome extending from ORFK3 to ORF19, with very high level of reads mapping to ORFK5 (Fig 2, bracketed). This transcript pattern was unique to this tumor and was not seen in the paired tumor from the same individual (008_C) or any other KS tumor. This sample is further described below.

**Fig 3:**
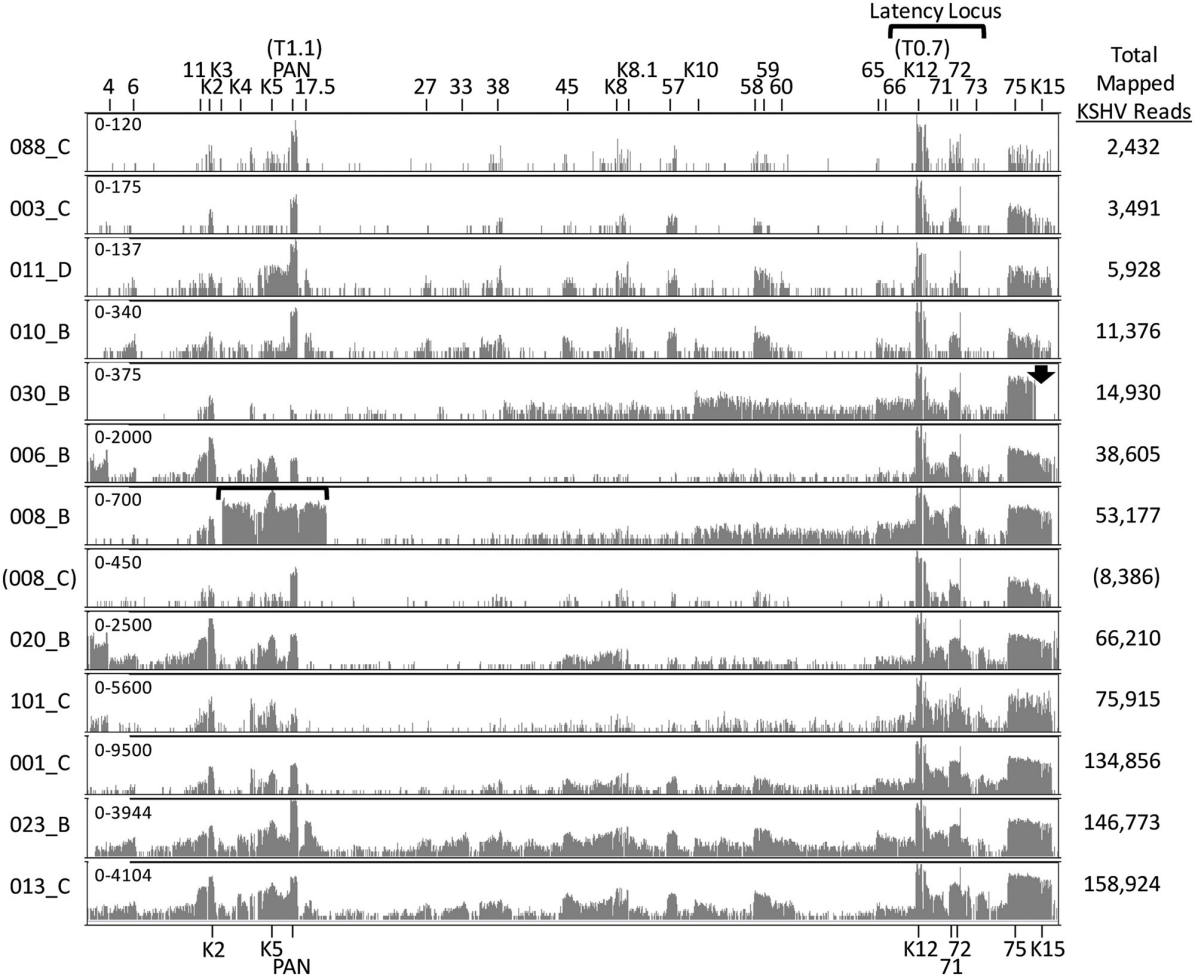
KSHV transcriptome analysis in Ugandan KS tumor biopsies—Log scale. RNA-seq analysis using a linear scale described in Fig 2 was also performed using a log scale to visualize the complete range of mapped reads. See legend to Fig 2.

To analyze the complete range of read depths across the KSHV genome, the mapped reads were also visualized using a log-based scale (Fig 3). This analysis revealed the presence of RNA reads mapping across the complete KSHV genome with concentrations in regions encoding specific genes associated with lytic replication (ORFs 6, 59, 60), gene regulation (ORFs K8, 57), virion structure (ORFs 11, 17.5, 27, 33, 38, K8.1, 65, 66) and immune modulation (ORFs 4, K2, K3, K4, K5, 45, K10) (Fig 3). Similar patterns of transcription were visually observed in the different KS tumors regardless of the total number of KSHV-mapped reads in each sample (see for example, tumors 11_D and 013_C, with 5,928 and 158,924 total KSHV-mapped reads, respectively). This similarity was also observed in the 7 KS tumors containing less than 1,000 total KSHV-mapped reads (S1 Fig), suggesting that overall the majority of KSHV-infected cells in the KS lesions expressed the same basic transcriptome pattern. Of note, occasional KS samples, such as 030_B, had minimal levels of reads mapping to ORFK15, even though high levels of reads mapped to the adjacent ORF75 (Fig 3, arrow). Different alleles of ORFK15 have large sequence variation extending into the ORF75 sequence [36, 37], which could have affected the ability of ORFK15 reads in the Ugandan tumors to map to the NC_009333 reference sequence.

### Phylogenetic analysis of ORF75 reveals a wide diversity of KSHV strains in Ugandan KS tumors

**Fig 4:**
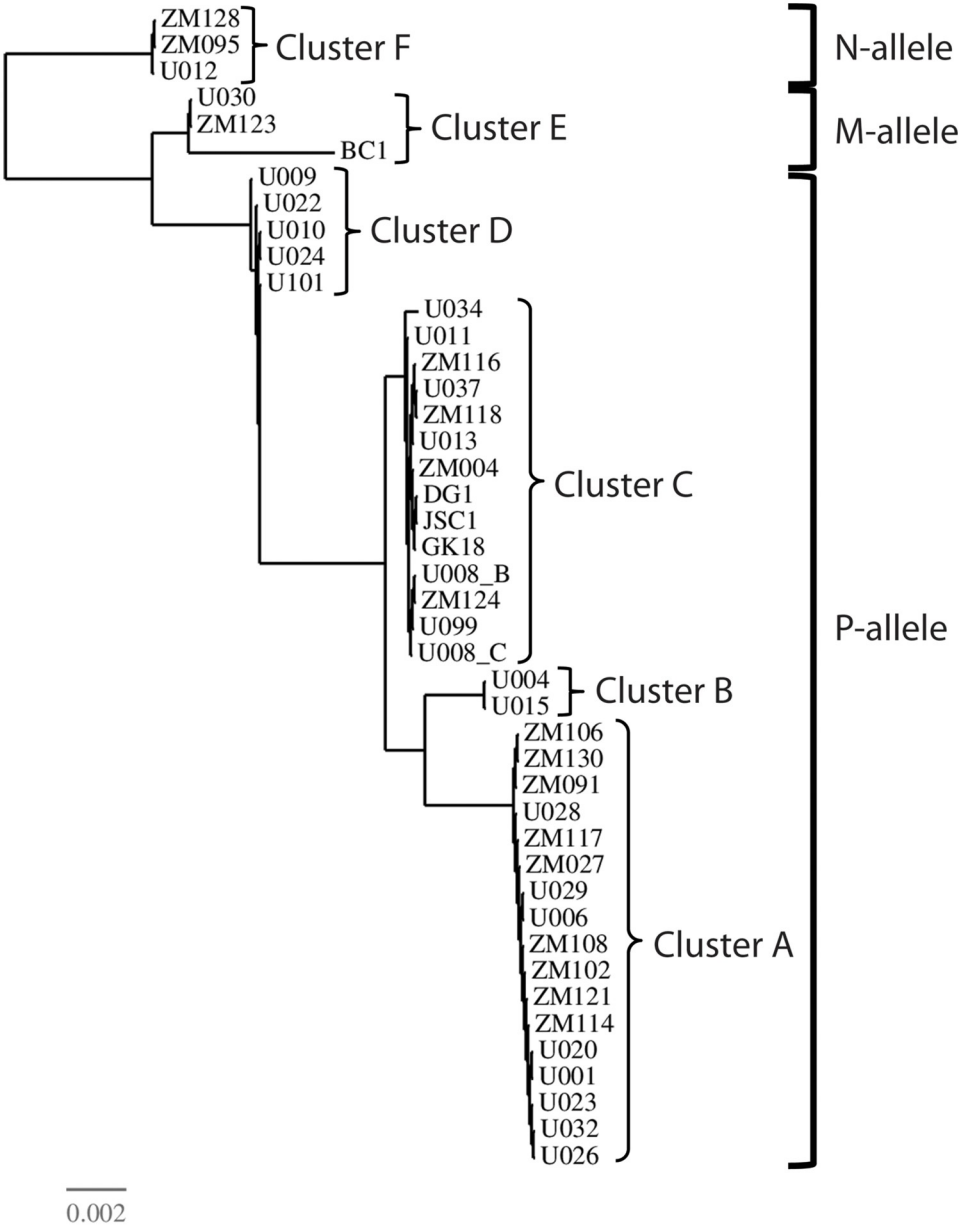
Phylogenetic analysis of the KSHV ORF75 sequences in 23 Ugandan KS tumor biopsies. The complete 3891 bp sequences of the KSHV ORF75 from the KSHV strains in 23 Ugandan KS tumor samples were assembled from overlapping 50 bp RNA reads and compared to the published ORF75 sequences of KSHV strains from 14 Zambian KS biopsies: ZM004 (KT271453), ZM027 (KT271454), ZM091 (KT271455), ZM095 (KT271456), ZM102 (KT271457), ZM106 (KT271458), ZM108 (KT271459), ZM114 (KT271460), ZM116 (KT271461), ZM117 (KT271462), ZM118 (KT271463), ZM121 (KT271464), ZM123 (KT271465), ZM124 (KT271466), ZM128 (KT271467), and ZM130 (KT271468). In addition, ORF75 sequences from the KSHV reference sequence strain GK18 from an HIV-negative individual with classical KS (NC_009333), the strain JSC1 from an HIV infected male with a lymphomatous peritoneal effusion (GQ994935), the strain DG1 from HIV infected Caucasian male with KS (JQ619843) and the strain BC-1 from a primary effusion lymphoma (U75698) are compared. The sequences were aligned using ClustalX and the phylogenetic relationship was determined by maximum likelihood. Separate clusters of sequences (A-F) were identified. Sequences associated with the M-, N-, and P-alleles of the adjacent K15 subtypes are shown. KS tumors 008_B and 008_C were obtained from the same patient. The scale of divergence is indicated.

The RNA-seq mapping studies were performed by aligning the RNA reads from the different Ugandan KS tumors to the GK18 NCBI Reference sequence (NC_009333) present in a patient with a case of the classic (HIV-negative) KS. The IGV view of the aligned reads from the Ugandan KS tumors revealed numerous mismatches indicating the presence of different KSHV strains in the KS tumor samples. To perform an initial phylogenetic analysis of these strains, we compared the RNA sequences of the large open reading frame encoding ORF75, which was highly expressed in nearly every KS tumor sample. Although most previous studies of KSHV phylogeny have used the widely divergent K1 and K15 sequences [36, 38], these genes, especially K1, were too minimally and inconsistently expressed in the KS tumors to obtain sufficient sequence for phylogenetic analysis. The complete coding sequences of ORF75 (3891 bp) were assembled from the 50 bp reads for 23 of the Ugandan KS samples. These sequences were aligned with published OR75 sequences from 16 unique KSHV genomes from Zambia [39] and ORF75 sequences in the NCBI database from KSHV strains in several KS biopsies and PEL cell lines obtained in Western countries. Maximum likelihood analysis revealed two major (A, C) and four minor ORF75 clusters (B, D-F) (Fig 4). A BLAST alignment with ORF75 partial sequences that have been previously typed [40] indicated that Cluster A corresponded to subtype [B], Clusters B and C corresponded to subtype A/[B], Cluster D corresponded to subtype R/A, Cluster E corresponded with subtype R/M and Cluster F corresponded with subtype N. The vast majority of Zambian sequences were distributed in the major clusters A and C. Previous studies have shown that ORF75 sequences are linked to the adjacent K15 subtypes, of which 3 distinct alleles P, M and N have been detected [36]. The majority of the ORF75 sequences, including those in Clusters A-D were linked to the K15 P-allele (Fig 4), which was present in the GK18 reference sequence. In contrast, the U030 and ZM123 sequences, like the BC1 sequence, were linked to the M-allele, while U012, ZM095 and ZM128 [39], were linked to the K15 N-allele. The sequence differences between the K15 P-allele of GK18 and the more distantly related M and N alleles resulted in essentially no RNA reads from the U012 and U030 tumor samples aligning with the K15 region of GK18 (see Fig 3). Although our phylogenetic analysis was based on a single gene, it is clear that the KSHV strains infecting the different Ugandan KS tumors show high variability, with strong similarity to KSHV strains identified previously in the Zambian KS samples [39]. Overall, the RNAseq analysis indicated the presence of a single KSHV strain in each tumor. In all cases, but subject 008 (discussed below), independent tumors from the same patient contained the same KSHV strain.

### Transcript mapping in the highly expressed KSHV latency locus

**Fig 5:**
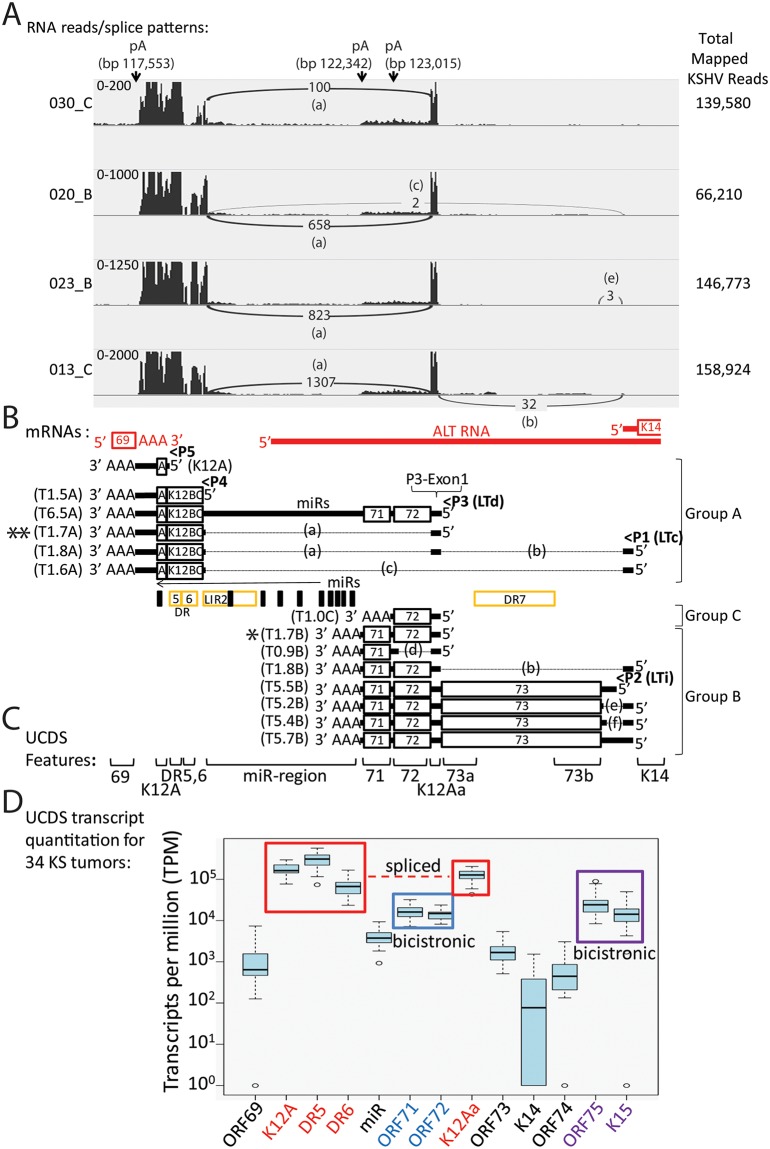
Quantitation of RNA reads mapping to the KSHV latency region. RNA reads from 34 Ugandan KS tumor biopsies were mapped to the KSHV genomic region from ORF69 to K15 using TopHat2. A) Representative spliced transcript patterns (Sashimi plots) for four KS tumors are shown for genes in the latency locus. The vertical axis represents the number of reads aligned to each nucleotide position of the KSHV reference genome (autoscaled for the height of reads mapping to the 5’exon downstream of promoter P3(LTd), described in the text; the visible axis range is shown on the left). The total number of reads mapping to the KSHV genome is indicated at the right. The number and position of split reads detected by TopHat2 indicating donor and acceptor sites for spliced transcripts are shown and the corresponding excised intron region is labeled. The positions of poly-A sites of transcript termination are shown: ORF69 (bp 117,509); T0.7 and related Group A transcripts (bp 117,553); ORF71 and related Group B transcripts (bp 122,342); ORF72 monocistronic Group C transcript (bp 123,015), as described in the text. B) The proposed transcription pattern for the latency locus is shown (black = sense strand; red = antisense strand). The proposed non-coding antisense ALT RNA and the positions of the direct repeats DR5, 6 and 7, the long inverted indirect repeat (LIR2) and the microRNA region (miRs) are indicated. The positions of the promoters for the latency transcripts are shown: P1 (LTc; bp 128,089), P2 (LTi; bp 127,789), P3 (LTd; bp 124,131), P4 (~bp 119,035), and P5 (~bp 118,234) promoters and the known latency transcripts are graphically represented. Coding sequences are boxed, adjacent 5’ and 3’ regions are thick lines and introns (a-f) are indicated as thin dotted lines (intron a: bp 123,842…119,048; intron b: bp 127,961…124025; intron c: bp 127,961…119,048; intron d: bp 123,842…123,108; intron e: bp 127,961…127,463; intron f: bp 127,961…127,619). The transcripts terminating at the poly-A site downstream of the Kaposin coding sequence (panel A; bp 117553) are grouped together (Group A), while those terminating at the poly-A site downstream of ORF71 (panel A; bp 122342) are grouped together (Group B). The monocistronic ORF72 Group C transcript terminates at the poly-A site bp 123,015. The major T1.7A spliced transcript in Group A encoding the K12 A/B/C complex is indicated with a double asterisk, while the major T1.7B bicistronic transcript in Group B encoding ORF71/72 is shown with a single asterisk. The first exon (P3-Exon1) in the transcripts derived from promoter P3(LTd) is indicated. The sizes of the transcripts are derived from the GK18 strain (NC_009333 reference sequence) (see S2 Table). The DR5, DR6 and DR7 repeat regions contain different numbers of repeats in different KSHV strains, yielding variation in lengths of the Group A and B transcripts [44]. C) The non-overlapping UCDS features for quantitating transcripts are shown. UCDS features for ORFs 69, 71, 72 and K14 correspond to the coding sequences for these ORFs. The K12A UCDS feature corresponds to the T0.7 transcript, while the K12Aa UCDS feature corresponds to the P3-Exon1 downstream of the P3 (LTd) promoter (bp 124,054‥123,843). The miR UCDS feature identifies reads from unspliced mRNAs, such as the 6.4Kb transcript, which map to the genomic region containing the right long inverted repeat (LIR2) encoding the microRNAs K1-9. The miR UCDS feature does not detect processed miRNAs, as these RNAs are not polyadenylated and thus are not present in the sequenced RNA-seq library. The ORF73a and ORF73b UCDS features target the non-repetitive regions of the ORF73 transcript, and reads are combined for quantitation. The DR6 UCDS feature targets an extremely GC-rich repetitive region. D) mRNA reads mapping to the latency region extending from the flanking ORF69 to the terminal K15 were quantitated for 34 KS tumors using the UCDS features and normalized (TPM), and the median and interquartile range was plotted using an R-based BoxPlot algorithm, see Materials and methods. The reads mapping to the K12Aa, DR6, DR5 and K12A UCDS features are boxed in red to indicate their relationship within the T1.7A spliced transcript, while the reads mapping to the ORF71/ORF72 and ORF75/K15 are boxed in blue and purple, respectively, to indicate their relationship within bicistronic transcripts T1.7B (single asterisk, Panel B) and T6.3, respectively.

To accurately correlate the RNA reads to specific mRNA transcripts in the latency region, we previously developed a map of transcripts that had been identified in the literature [25]. Since these transcripts were initially characterized in different KSHV strains with different sizes, their positions were mapped onto the sequence of the NCBI reference sequence for KSHV, strain GK18 (NC_009333), and the sizes of the GK18 transcripts were predicted from the transcription start and polyadenylation (poly-A) termination sites (S2 Table). A map of the spliced and unspliced latency region transcripts is shown in Fig 5B, with the transcripts grouped according to their use of the poly-A termination sites at bp 117,553 (Group A), bp 122,342 (Group B), and bp 123,015 (Group C). Due to heterogeneity in the DR5, DR6 and DR7 repeat regions, the sizes of the transcripts from the GK18 strain vary from the published transcript sizes determined for other KSHV strains, such as BCBL-1, as indicated in the corresponding references (summarized in S2 Table).

To analyze the read depth and splicing events in the latency region, Sashimi plots were produced by IGV from the TopHat2 analysis of the RNA reads for the KS tumor samples. Sashimi plots graphically present the read depth across a selected region and show the location and quantity of split RNA reads that define the presence of a splicing event within an mRNA transcript. Sashimi plots of four representative KS tumor samples (Fig 5A) were aligned with the different spliced and unspliced transcripts generated from the latency region (ORFs 69-K14) (Fig 5B). This alignment clearly showed a high level of RNA reads mapping to the T0.7 RNA (herein designated as T0.7A) from the P5 latency promoter, which encodes Kaposin A. In addition, a high level of reads mapped to the adjacent DR5 and DR6 repeat regions, even though the TopHat analysis limited reads to a single map position. A number of spliced and unspliced transcripts that terminate at a common poly-A site (bp 117,553) downstream ORF K12 contain the repeat regions. These transcripts are derived from different latency transcript (LT) promoters and include the spliced T1.6A and T1.8A transcripts from the P1/LTc (constitutive) promoter, the spliced and unspliced T1.7A and T6.5A transcripts from the P3/LTd, (downstream) promoter, and the unspliced T1.5A transcript from the P4 promoter (Fig 5B).

Analysis of the Sashimi plots across the latency region revealed a high level of split reads mapping between the highly expressed DR6 repeat region and the highly expressed genomic region downstream of the P3 (LTd) promoter, herein designated as P3-exon1 (Fig 5A and 5B), which was indicated in Fig 2 with an asterisk. Examination of the corresponding sequences in the KSHV genome revealed classical splice donor (AG|gt) and acceptor (ttacgcccccttcgcag|G) sites at bp 123,843 and bp 119,047, respectively. These sites define the presence of a 4,796 bp intron (Fig 5B; labeled “a”), which is spliced from the pre-mRNA for transcripts T1.7A and T1.8A, and encompasses the sequences encoding ORFs 72 and 71, the right origin of replication and flanking long inverted repeat (LIR2) and the microRNAs miR K1-9 and 11. A high level of reads split across intron “a” were detected in the vast majority of KS tumors (Table 2). The depth of the split reads across intron “a” (for ex. see Fig 5A, ranging from 100 reads (030_C) to 1,307 reads (013_C); Table 2) was similar to the depth of the reads in the flanking exons suggesting that the majority of the reads mapping to the K12/DR5/DR6 region were derived from the T1.7A spliced transcript (Fig 5B, double asterisk). This conclusion was confirmed by RT-PCR amplification of the spliced T1.7A transcript from four KS biopsies using primers derived from the K12 region and the exon junction spanning the spliced “a” intron (S2 Fig). While there was evidence for the splicing of intron “b” in some tumors (see for example Fig 5A: 013_C- “b” = 32 reads; Table 2), compatible with the processing of transcript T1.8A from the upstream P1 (LTc) latency promoter, there was no corresponding accumulation of RNA reads mapping to the 5’ exon of this transcript (Figs 3 and 5A). Thus, the splicing data indicates that the high level of split reads across the K12/DR5/DR6 region and P3-Exon1 are derived from the spliced T1.7A transcript initiating from the P3 (LTd) promoter, which is highly expressed in all the KS tumors.

**Table 2:** Quantitation of split reads defining spliced latency transcripts.

KS biopsy	Intron^1^					
	“a”	“b”	“c”	“d”	“e”	“f”
001_B	428					
001_C	1689					
003_C	45					
004_D	8					
006_B	481	8				
006_C	186					
007_B	19					
008_B	156					
008_C	130					
009_B	31					
010_B	90					
011_C	1					
011_D	1					
012_D	1011	3	1			
013_B	554	20	2			
013_C	1345	33				
015_B	515					
020_B	693		2			
022_B	45					
023_B	859				3	
024_B	1048	10	2			
026_B	181					
026_C	177					
028_B	50					
029_B	337					
029_C	99					
030_B	105					
030_C	1030				3	
032_B	7					
034_B	15					
037_B	153					
088_C	62					
099_C	2694					
101_C	2691		1			

^1^Reads split across splice junctions for introns “a-f” in transcripts from the latency region, as designated in Fig 5, were quantitated *ab initio* by TopHat2 analysis

### Quantitation of KSHV transcripts

Quantitation of transcripts from complex genomes, such as KSHV, has been difficult due to the compact nature of the genome and the presence of numerous overlapping transcripts that are differentially expressed. We developed a novel approach to quantitate RNAseq reads mapping to specific genes and gene features in the highly complex KSHV genome using unique coding sequence (UCDS) features specific for all known KSHV ORFs and transcriptional regions [25]. Published information and transcript data from RNAseq analysis of primary latent KSHV infections of several endothelial cell types and long-term latent infections in PEL cells were used to identify transcription start and termination sites and globally map mRNA transcripts. The UCDS features were devised to be non-overlapping, separated by the length of a read (50 bp) so that algorithms, such as HTSEQ, using the “intersection_nonempty” setting could specifically identify and distinguish reads mapping to the different targeted gene features on both DNA strands. RNA read depth was determined by quantitating the reads aligning to the UCDS features in a simplified gene feature file (KSHV NC_009333 UCDS ver 020116.gff; S1 File) based on the KSHV reference genome NC_009333 [25]. The read count was normalized to the size of the feature and number of total KSHV-mapped reads in each tumor sample, yielding a relative transcript level, in transcripts per million KSHV reads (TPM), as described in Materials and Methods. Read data and normalization for each KS sample is provided in S3 Table This normalization allowed the expression of each KSHV gene to be compared as a proportion of the total number of KSHV-mapped reads in each sample. In this and subsequent quantitative analyses of read depths across the KSHV genome, the analyses were limited to 34 of the 41 KS tumor samples, which had sufficient levels of RNA reads mapping to the KSHV genome (>1000) for objective comparisons (see Fig 1). The median normalized level of transcripts targeted by our set of UCDS features across the entire KSHV genome for the 34 samples is provided in S4 Table. To more accurately reflect the protein coding potential for genes in regions with overlapping polycistronic transcripts, we determined the level of the primary transcripts for each ORF derived from its associated promoter, in which the ORF would be the first in the transcript to be translated through 5’ CAP-dependent initiation. The contribution of overlapping transcripts derived from distal promoters was subtracted, yielding an estimation of the primary transcript levels for each ORF (S4 Table), as discussed previously Bruce et al [25].

In the latency and flanking regions, UCDS features were identified targeting the coding sequences for the ORFs K12A (T0.7), 72, 71, 73, K14, 74, 75 and K15, as well as additional areas of interest, including the direct repeats DR5 and DR6, the region encoding microRNAs miR-K1-9, 11 (miR-region), and the short P3-Exon1 downstream of the ORF72 P3(LTd) promoter (K12Aa) (Fig 5C). Quantitation of the reads mapping to the latency region from the 34 KS tumor samples revealed very high transcript levels for the K12/T0.7 region (UCDS K12A) and flanking DR5 (UCDS DR5) and DR6 (UCDS DR6) repeat regions (median = 162,467, 310,389 and 65,018 TPM, respectively) (Fig 5D, boxed in red; S4 Table). Although the known transcripts in this region span both the DR5 and DR6 region, transcribed right to left (Fig 5B), significantly less reads mapped to the DR6 UCDS feature, which would detect the 5’ region of such transcripts. The DR6 repeat and upstream flanking regions are known to have significant sequence heterogeneity between different KSHV strains [41], suggesting that the relatively lower level of reads mapping to the DR6 UCDS feature could be due to mismatches between the reads from the Ugandan KSHV strains and the NC_009333 KSHV reference sequence used for the mapping. Very high levels of transcripts (median = 123,915 TPM) were detected using the K12Aa UCDS feature (Fig 5D, boxed in red), which targets the small P3-Exon1 downstream of the P3(LTd) promoter (Fig 5B and 5C). The similarity in the transcript levels detected across the K12A, DR5, DR6 and K12Aa UCDS features (Fig 5D, boxed in red) correlates with the presence of the common spliced transcript (T1.7A) derived from the P3(LTd) promoter, which encodes the Kaposin A/B/C complex (Fig 5A, double asterisk), as described above. This was confirmed by RT-PCR of RNA from 4 KS tumor biopsies (S2 Fig).

Moderate levels of transcripts (median = 15,794 and 14,430 TPM) were detected using UCDS features targeting ORF71 and ORF72, respectively (Fig 5D; S4 Table). Previous studies have determined that the major transcripts encoding ORFs 71 and 72 are either bicistronic (5’ ORF72/ORF71 3’) initiating from the P3(LTd) promoter or tricistronic (5’ ORF73, ORF72, ORF71 3’) initiating from either the P1(LTc) or P2(LTi) promoters (Fig 5B; S2 Table). The similarity in the read depths detected using the ORF71 and ORF72 UCDS features suggests that the majority of these reads are derived from the major unspliced bicistronic transcript (T1.7B) from the P3(LTd) promoter (Fig 5A, single asterisk). While these reads could also be derived from the common spliced bicistronic transcript (T1.8B) from the P1(LTc) promoter (see Fig 5B), only a small number of reads mapped to the 5’ exon of this transcript near the P1(LTc) promoter (see Fig 5A). Only a low level of transcripts (3,300 TPM) were detected with the miR UCDS feature, which targets transcripts containing the miR region flanking the LIR2 (Fig 5C), suggesting that the majority of the transcripts encoding ORF71 terminated at the poly-A site (bp 122,342) downstream of ORF71 (Fig 5B). Quantitation of the reads mapping to the non-repetitive regions of ORF73 using the ORF73A and ORF73B UCDS features revealed low levels of the tricistronic transcripts T5.2B, T5.4B, T5.5B and T5.7B encoding ORFs 73, 72 and 71 (Fig 5D; S4 Table). This supports the conclusion that the majority of the ORF71 and ORF72 reads map to the unspliced bicistronic T1.7B transcript from the P3(LTd) promoter and not from transcripts from the P1(LTc) promoter.

To further analyze transcription across the ORF72/71 locus, we determined the ratio of transcripts detected using the ORF72 and ORF71 UCDS features. The ORF71/ORF72 ratios in the different tumors ranged from 0.7 to 2.1, suggesting differential expression of the two ORFs that was not strictly compatible with the single bicistronic T1.7B transcript (S3 Fig). Seven tumors showed higher levels of transcripts encoding ORF72 (ORF71/72 ratios from .7 to 0.9) (S3A Fig) indicating the presence of a ORF72 monocistronic transcript T1.0C (Fig 5B), identified previously by Sarid et al [42]. Twenty-one tumors showed higher levels of transcripts encoding ORF71 (ORF71/72 ratios from 1.1 to 2.1) (S3A Fig), indicating the presence of a ORF71 monocistronic transcript T0.9B (Fig 5B), identified previously by Grundhoff and Ganem [43].

### Conserved nucleotide changes in the Ugandan KSHV strains alter the protein coding potential of the highly expressed T1.7A transcript

**Fig 6:**
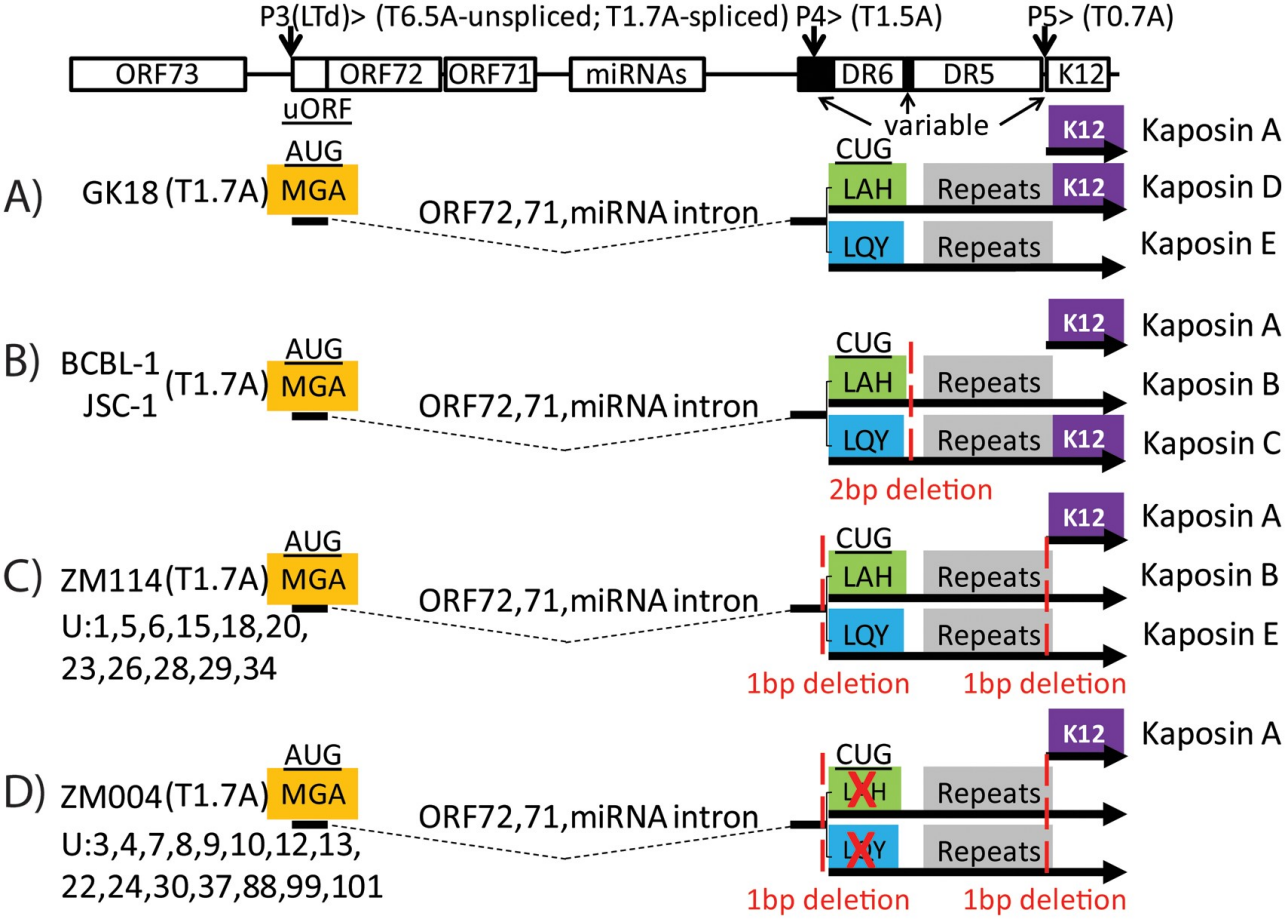
Sequence variants of the latency region transcripts. The structure of the KSHV genome across the latency region is shown in the direction of transcription, with the positions of the encoded major ORFs, small upstream ORF (uORF), microRNAs (miRNA), and direct repeats (DR) indicated. The positions of the known promoters and the regions of sequence variability in the repeat regions are also shown. The proposed spliced T1.7A transcripts from the P3 (LTd) promoter and non-spliced transcripts T1.5A and T0.7A from the P4 and P5 promoters, respectively, are shown. The AUG initiating codon for the upstream “MGA” encoding uORF sequence and the CUG initiating codons for the two different downstream repeat open reading frames “LAH” and “LQY” are indicated, as is ORFK12 encoding Kaposin A. A) Putative translation products from the GK18 reference strain are shown: Kaposin D containing the repeat sequence initiating with “LQY” that lacks the Kaposin A sequence and Kaposin E containing the repeat sequence initiating with “LAH” that is fused in tandem with Kaposin A. B) The previously described translation products from the BCBL-1 cell line are shown: Kaposin A alone, Kaposin B containing the repeat sequence initiating with “LAH” (major gene product) and Kaposin C containing the repeat sequence initiating with “LQY” fused in tandem with Kaposin A (minor gene product)[44]. The BCBL-1 genome contains a 2 bp deletion between the DR5 and DR6 repeat regions compared to GK18 (red dotted line). C, D) Putative translation products from the Zambian KSHV strains, ZM114 and ZM004, are shown, which contain 1 bp deletions upstream of the “CUG” initiation region and 1 bp deletions between the DR5 repeat and the ORFK12 coding sequence (red dotted lines). D) Putative translation products from the Zambian KSHV strain, ZM004 are shown, which contains additional nucleotide changes eliminating the “LAH” and “LQY” ORFs, as described in the text. The Ugandan KS tumors with the same sequence changes as ZM114 and ZM004 are indicated.

Kaposin A is part of a complex translational program that generates multiple novel proteins from the K12 locus. The Kaposin A sequence is downstream of the DR5 and DR6 repeat sequences, which encode variant translation products initiating at alternate CUG initiation codons that have been detected in cultured PEL cells [41, 44]. The CUG initiation codons occur in the sequence region upstream of the DR6 repeat region, which is highly variable in different KSHV strains [41]. Previous studies identified a non-spliced transcript from a promoter, herein referred to as P4, corresponding to T1.5A (see Fig 5B), whose major translation product in the KSHV strains present in BCBL-1 and JSC-1 cells was a CUG-initiated open reading frame encoding a highly repetitive protein sequence, with an “LAH” N-terminal sequence (Kaposin B)[44](Fig 6B). A minor translation product, Kaposin C, initiated from an alternate downstream CUG initiation codon with a “LQY” N-terminal sequence. Kaposin C contained a similar repetitive protein sequence as Kaposin B but was fused to Kaposin A [44] (Fig 6B). Functional studies have shown that the BCBL-1 Kaposin B can modulate mRNA turnover by stabilizing cytokine mRNAs [45]. However, the functions of Kaposins A and C are as yet unclear, as functions attributed to Kaposin A, such as transforming potential [30], have been further attributed to the microRNA miR-K10, which is embedded within the Kaposin A coding sequence [46].

Comparison of the K12 locus of the BCBL-1/JSC KSHV strain with the GK18 reference strain revealed a 2 bp deletion in the BCBL-1/JSC-1 sequence between the DR6 and DR5 repeat regions (Fig 6A and 6B). This insertion alters the open reading frames such that the “LAH” initiated open reading frame from the upstream CUG codon in GK18 is now fused with Kaposin A, suggesting that the major translational product of the GK18 K12 locus would be a novel protein, herein termed “Kaposin D” (Fig 6A). This indicates that the minor translation product of the GK18 K12 locus from the downstream CUG initiation codon would be a novel protein “Kaposin E”, with the N-terminal sequence “LQY”. Unlike Kaposin C in the BCBL-1 strain, Kaposin E would lack the downstream Kaposin A fusion. The putative DNA and encoded protein sequences in this region are provided in S4 Fig.

The RNA sequence data in our study revealed several nucleotide differences between the Ugandan KSHV strains and both the GK18 and BCBL-1/JSC-1 strains. A single nucleotide deletion was detected downstream of the P4 promoter before the CUG initiation codons in all the Ugandan KS samples (Fig 6C and S4 Fig). All Ugandan KSHV strains also had an additional nucleotide deletion between the DR5 repeat and the Kaposin A reading frame (“A” bp 118,228, NC_009333). The deletion of this base, which is also detected in all the published Zambian KSHV strains [39], changes the open reading frame through the repeat region that is contiguous with the Kaposin A sequence downstream. In a subset of Ugandan KSHV strains, from KS tumors 001, 005, 006, 015, 018, 020, 023, 026, 028, 029 and 034, this deletion results in neither open reading frame from the two CUG initiation codons creating a fusion with Kaposin A, as exemplified by the Zambian KSHV strain, ZM114 (Fig 6C). Thus, these KSHV strains would encode Kaposin B as the major CUG-initiated translation product and Kaposin E, as the minor-CUG initiated product, with Kaposin A as a downstream AUG-initiated product. The remaining Ugandan KSHV strains, from KS tumors 003, 004, 007, 008, 009, 010, 012, 013, 022, 024, 030, 037, 088, 099, and 101 had additional sequence differences that altered the possible protein products expressed from this locus. In these strains, the major CUG-initiated ORF, Kaposin B, was eliminated by a change in the putative “CUG” initiation codon to “CGC” (Fig 6D and S4 Fig). In addition, the minor CUG-initiated ORF, Kaposin C, was eliminated by a change in the codon flanking the “CUG” initiator from “CGA” to the stop codon “TGA”, thus immediately terminating translation. A similar situation was seen in the Zambian KSHV strain, ZM004 (Fig 6D), and was confirmed by PCR amplification and sequencing.

**Fig 7:**
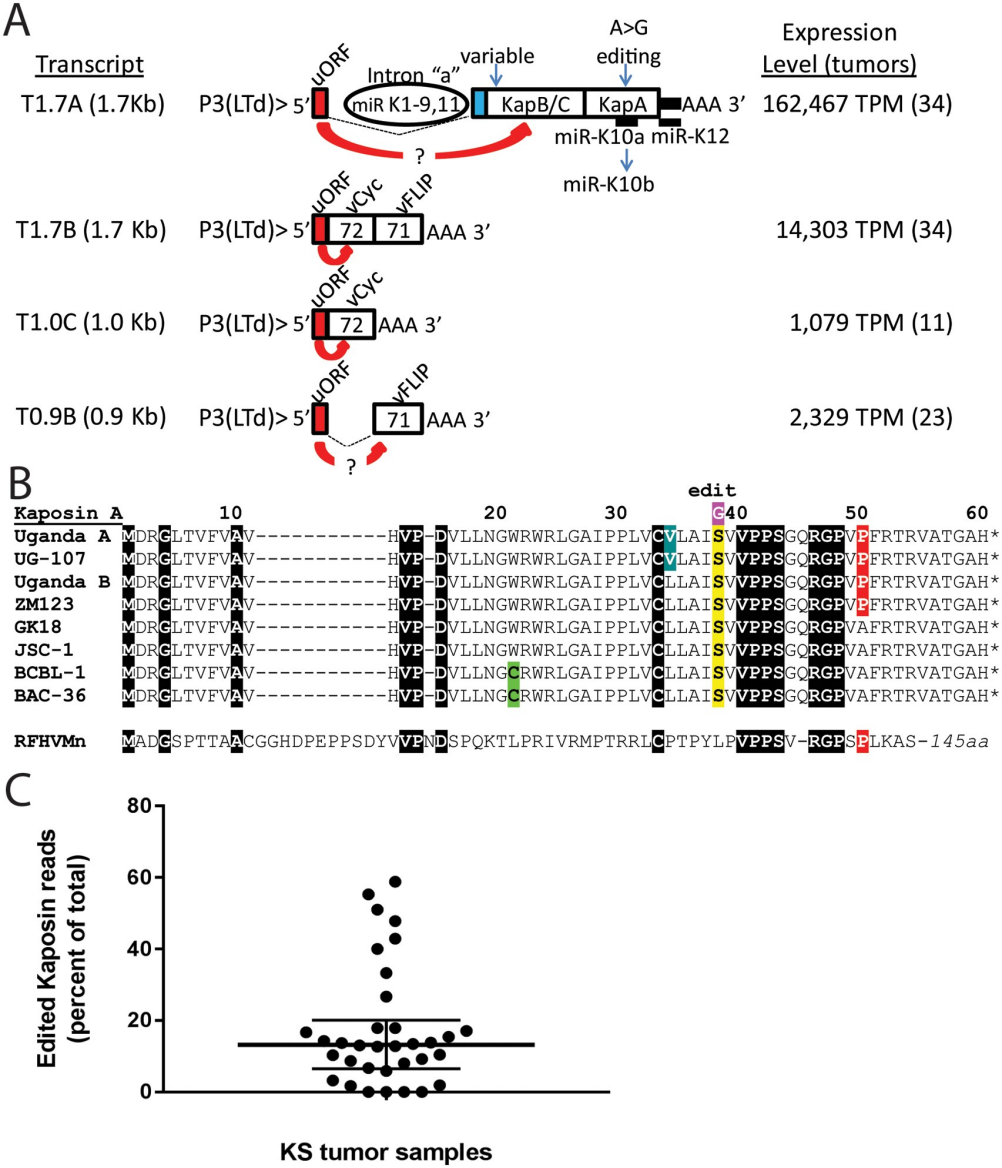
Analysis of highly expressed transcripts from the P3(LTd) latency promoter. A) A diagram of the coding potential of the spliced and unspliced transcripts derived from the P3(LTd) latency promoter is shown. The presence of a regulatory uORF, introns and coding ORFs are shown. The regulatory uORF has been shown to downregulate ORF72 expression in the ORF71/72 bicistronic transcript, as discussed in the text, and is predicted to downregulate KapB/C in T1.7A and vFLIP in T0.9B. The variable region upstream of the Kaposin B/C (KapB/C) coding sequence and the position of RNA editing affecting the coding sequence of the Kaposin A (KapA) and miR-K10a seed sequence are indicated, as are the median expression levels for the different transcripts in the KS tumor samples. B) Alignment of the Kaposin A variants. Published sequences: (UG-107: ACZ92060; ZM123: KT271465; GK18: NC_009333; BAC-36: HQ404500; Retroperitoneal fibromatosis herpesvirus *Macaca nemestrina* (RFHVMn): AGY30757); Sequences this study: (Uganda A group: 003, 007, 009, 010, 013, 022, 024, 030, 032, 037, 099, 101; Uganda B group: 001, 004, 006, 008, 011, 012, 014, 015, 018, 020, 023, 026, 028, 029, 034, 088; BCBL-1). Additional unique variations from the Uganda group A and B sequences included: Q14-P14 (011), W20-G20 (023), P40-A40 (034) and unknown sequence variation (no mapped reads) after R54 (006). C) The percent of reads containing the edited base at position 118,096 of NC_009333, resulting in the change from miR-K10a to miR-K10b (panel A) and the change from Ser39 to Gly in Kaposin A (panel B) is shown for all 34 KS tumor samples, with median and interquartile range. Edited reads: median = 6.5, IQR = 0–89; Total reads: median = 79, IQR = 5–161.

Small 5’ upstream ORFs (uORFs) are known to regulate expression of downstream ORFs by capturing or slowing the progression of ribosomes scanning the transcript for favorable initiation codons [47]. Previous studies have shown that a small 24 aa uORF is encoded upstream of ORF72 in the initial P3-exon1 of the T1.7B bicistronic ORF72/71 transcript from the P3(LTd) promoter (Fig 7A). This uORF attenuates the expression of the ORF72 10–20 fold in the T1.7B bicistronic transcript [43], and would be expected to attenuate ORF72 expression in the T1.0C monocistronic transcript and ORF71 expression in the T0.9B monocistronic transcript (Fig 7A). This uORF is also encoded in the initial 5’ exon of the highly expressed T1.7A spliced transcript from the same promoter and is positioned upstream of the ORFs encoding the Kaposin A/B/C complex (Fig 7A). Thus, this uORF would be expected to also attenuate translation of the Kaposin B/C complex proteins, especially since these proteins initiate from CUG codons.

### The highly expressed T1.7A transcript undergoes RNA editing and splicing to liberate an intron encoding KSHV microRNAs

The processing of the pre-mRNA for the T1.7A spliced latency transcript generates an RNA intron that is believed to be the source of the major KSHV microRNA species, miRs-K1-9 and 11 [48–50] (Figs 5B and 7A). Therefore, the high levels of the T1.7A spliced transcript could generate elevated levels of these microRNAs in the KS tumors. Since the other KSHV microRNAs, miR-K10 and miR-K12, are located within the retained exon in the T1.7A spliced transcript, such transcripts could also be processed to produce these microRNAs. Processed microRNAs are not detected in our RNAseq protocol, since they do not contain the 3’ poly-A region used for RNA purification. Thus, purification of small RNA species in the KS tumor samples would be needed to confirm the presence of KSHV microRNAs in the KS tumors.

RNA editing of bp 118,096 (NC_009333) (Fig 7A) has been shown to convert the transformation-associated miR-K10a containing an adenine in its seed sequence into the non-transforming miR-K10b containing an inosine [46]. We examined the RNA reads from the KS tumors for the presence of an edited base in the RNA reads. The fraction of ORFK12 transcripts containing an edited base ranged from 0 to 59% across the 34 KS tumors with a median of 13% (Fig 7C), suggesting that the transforming miR-K10a would be the major microRNA form produced in most of the KS tumors. Since miR-K10 is embedded within the mRNA transcripts encoding Kaposin A, RNA editing also affects the encoded Kaposin A protein sequence, changing a serine to glycine [51](Fig 7A and 7B). Our analysis revealed that only a fraction of the ORFK12 transcripts in the majority of KS tumors would encode the altered Kaposin A.

### Transcripts encoding ORF73 LANA are minimally expressed in KS tumors

The spliced and non-spliced Group B latency transcripts transcribed from the P1(LTc) promoter and P2(LTi) promoter terminate at the poly-A site downstream of ORF71 (Fig 5B, S2 Table). Although previous in vitro studies indicated that the P1 and P2 transcripts were the major latency-associated transcripts in PEL cells, there was evidence for only minimal expression in the KS tumors (Figs 3 and 5A). Only two tumors had evidence for splicing of intron “e” within the major latency-associated spliced ORF73/72/71 tricistronic transcript (T5.2B), with only 3 reads detected (Table 2). No evidence was detected for splicing of intron “f” (T5.4B), which is similar to intron “e” but from an adjacent splice acceptor site (“f” acceptor: bp 127,626 [6] compared to “e” acceptor: bp 127,462 [8]). Transcripts initiating from the P1(LTc) promoter upstream of ORF73 LANA were quantitated using the ORF73A and ORF73B UCDS features (Fig 5C). These features flank the large DR7 repeat region within the ORF73 coding sequence, which was not used for quantitation due to its repetitive nature. Consistent low levels of LANA reads (median = 2,195 TPM) were detected in the KS tumors (Fig 5D; S4 Table), indicating low levels of the T5.2B, T5.4B, T5.5B, and T5.7B ORF73/72/71 tricistronic spliced and unspliced transcripts derived from the P1(LTc) or P2(LTi) promoter. The low level of transcription from the P1(LTc) promoter correlates with the very low levels of split reads detected for introns “e” and “f” in the Group B transcripts (Fig 5A and Table 2). RT-PCR analysis of RNA from four of the KS tumor samples with the highest levels of KSHV-mapped reads failed to detect any of the tricistronic transcripts from the P1(LTc) or P2(LTi) promoters, confirming the RNAseq data (S2 Fig).

### ORF75 and K15 are consistently expressed at moderately high levels in KS tumors

Additional genes adjacent to the latency locus showed consistent and high-level expression in the KS tumors. These included ORF75 and K15 (Figs 2 and 3), which are expressed in the same orientation as the latency genes described above. While the vast majority of the KS samples showed comparable levels of reads mapping to both ORF75 and K15 (Fig 3), tumors from two individuals, 012 and 030, had high levels of reads mapping to ORF75 with no reads mapping to K15 (Fig 3, arrow). The different alleles of K15 have significant sequence variation extending into the ORF75 sequence. The phylogenetic analysis of the ORF75 sequences indicated that the KSHV strains in tumors 012 and 030 contain the K15 alleles N and M, respectively, thus reads from these strains do not map to the P-allele containing GK18 reference sequence. UCDS features were developed targeting the large ORF75 open reading frame (3,891 bp) (ORF75 UCDS) and the largest exon coding the C-terminal domain of K15 (464 bp) (K15a UCDS) from the heavily spliced K15 gene, which appears to be present in all K15 transcripts. Quantitation of the reads mapping to the ORF75 and K15a UCDS features revealed moderately high transcript levels across 32 KS samples (median = 21,490 and 15,866 TPM respectively) (Fig 5D; S4 Table). The KS samples from 012 and 030 were excluded, as their true read counts were not captured in this analysis. A single poly-A transcription termination signal has been identified for these transcripts downstream of ORF75 [52], suggesting that all of the transcripts detected by the K15a UCDS feature would be bicistronic, encoding K15 at the 5’ end and ORF75 at the 3’ end. Thirty of thirty-two tumor samples showed higher levels of ORF75 transcripts compared to K15 (Fig 5D; S4 Table; S3B Fig), indicating the presence of an ORF75 monocistronic transcript initiating upstream of the AUG initiator of ORF75. Quantitative analysis revealed that approximately two-thirds of the transcripts encoding ORF75 would be bicistronic, with K15 as the primary CAP-dependent translation product. The remaining transcripts would be monocistronic, with ORF75 as the primary translation product. It is not known whether ORF75 translation could occur through IRES-mediated initiation in the bicistronic transcripts, as was observed for ORF71 [43].

### Genomic translocation into the latency region induces high level expression of lytic genes

**Fig 8:**
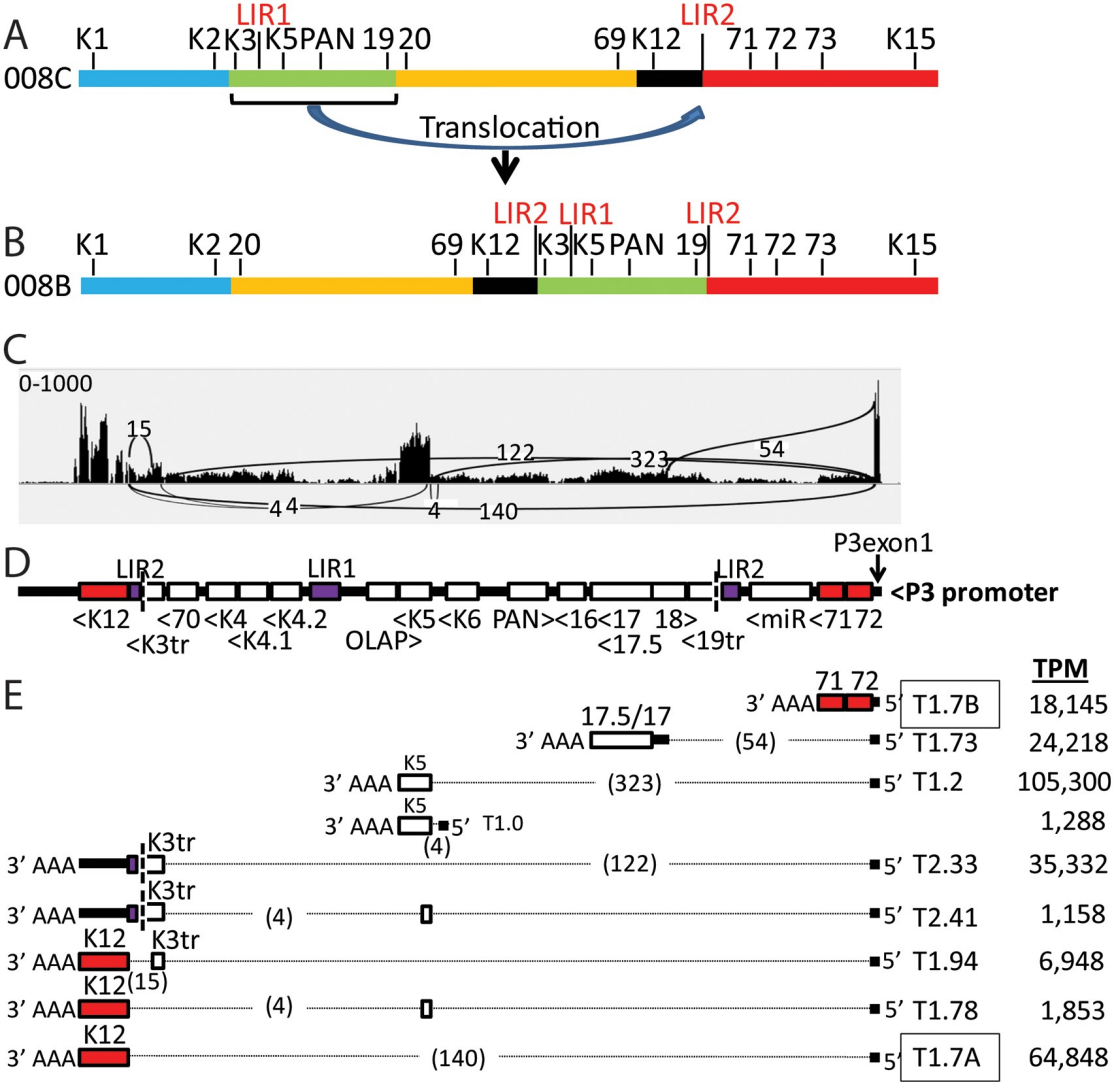
High level expression of a genomic fragment flanking LIR1, which was translocated into the latency region within LIR2 in KS tumor 008_B. A) Structure of the KSHV genome in KS tumor 008_C showing the position of a genomic fragment (ORFK3 to ORF19; green) that was translocated into the long-inverted repeat (LIR2) within the latency region in the KS tumor 008_B isolated from the same individual. B) Proposed structure of the KSHV genome in KS tumor 008_B with the ORFK3-ORF19 translocation. While the exact junction of the ORFK3-ORF19 translocated fragment was determined from the sequence of RNA reads mapping across the junction in LIR2, the junction of the genomic sequence across the excision point between ORFs K2 and 20 was not determined due to the absence of RNA reads in this region. C) Sashimi plot showing RNA read levels of genes between ORFs K12 and 72 mapping to a model genome of the KSHV reference sequence NC_009333 containing the proposed translocation. (Vertical axis: Range of RNA reads 0–1000). The position and quantity of split reads detected by TopHat2, indicative of transcript splicing, as discussed in Fig 5, is shown. D) Proposed position and orientation of genes translocated into LIR2 in the 008_B KSHV genome is indicated. The 3’ end of ORF-K3 (K3tr) and the 5’ end of ORF19 (19tr) are truncated at the LIR2 insertion point. The positions of the highly expressed P3(LTd) latency promoter and the initial 5’ exon (P3exon1), described in Fig 5, are indicated. E) Proposed transcripts derived from the P3 promoter with novel splicing patterns are shown. The highly expressed T1.7A and T1.7B transcripts detected in all the KS tumors, as indicated in Fig 5, are boxed. The numbers of reads split across each intron are shown in parentheses, and the transcript levels quantitated using specific UCDS features are indicated as transcripts per million (TPM) KSHV reads.

One KS tumor, 008_B, exhibited an unusual high level of reads mapping to the genomic region from ORFK3 to ORF19, as indicated above (Figs 2 and 3; bracketed in black) and in more detail (Fig 8A). The RNA reads at the borders of this region abruptly stopped in the middle of ORFK3 at one end and ORF19 at the other end, with no correspondence to possible RNA termination sites. High level expression of this region was not detected in KS tumor 008_C (Figs 2, 3 and 8A), which was isolated from a different location in the same patient, suggesting the presence of a genomic rearrangement in the KSHV strain in the 008_B tumor. By decreasing the alignment threshold in the initial Bowtie2 alignments, we identified reads containing partial sequences aligning to the ORFK3 and ORF19 regions flanking this highly expressed region. Sequence analysis of these reads revealed that a 14,813 bp ORFK3-ORF19 genomic region had been translocated from its original position at the left end of the genome (bp 19,168 to 33,980) to the right end of the genome within the long-inverted repeat (LIR2) (bp 119,504, numbering from the NC_009333 sequence) (Fig 8). A model genomic sequence of NC_009333 with this translocation was created and used to map the 008_B reads (as indicated in Fig 8). This analysis revealed high levels of spliced transcripts derived from the highly expressed latency promoter P3(LTd) upstream containing the 5’ P3-Exon1 (Fig 8D and 8E). Novel transcripts were detected in which the P3-exon1 was spliced to various acceptor sites in downstream genes within the translocated genome fragment. A spliced transcript encoding ORFK5 was expressed at the highest level. The majority of other genes contained in the translocated region were also highly expressed with non-spliced transcripts driven by their original promoters. Since high level expression of these genes was not observed in the paired 008_C tumor, it appears that the translocation of this genomic region to the highly expressed latency region was responsible for the increased gene expression.

### Variable gene expression is detected outside of the latency region

**Fig 9:**
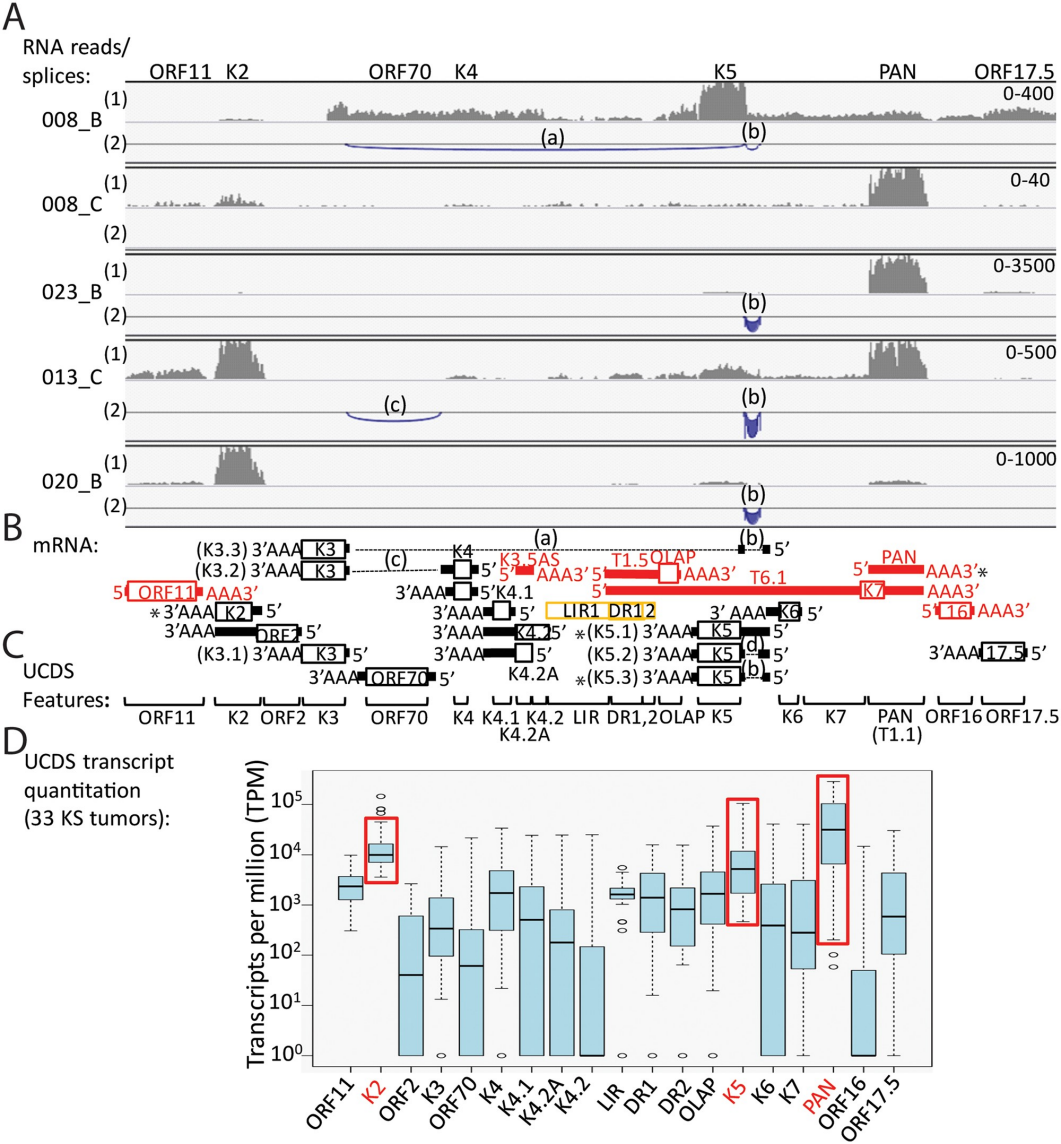
Analysis of the expression of PAN and flanking genes at the left end of the KSHV genome. A) The IGV view of reads mapping to spliced and non-spliced transcripts across the region encoding PAN and flanking genes is shown for 5 representative KS tumor samples. Tumors 008_B and 008_C were obtained from the same individual. The position and depth of mapped reads (linear scale) are shown in line (1) with the range indicated at the far right. The position of split reads mapping to splice junctions is indicated in line (2), where the height and thickness of the arc are proportional to the read depth up to 50 reads. The labeled introns are indicated on the transcript map compiled from published literature and predicted transcripts in (B). ORFs on the sense strand are shown in red, while ORFs on the complementary strand are shown in black. C) Position of UCDS features used for read quantitation. D) Total reads mapping to the UCDS features from 33 KS tumors (without 008_B) were quantitated and normalized (TPM). ORFs with high consistent levels of expression are indicated and boxed in red.

At the left end of the KSHV genome, high levels of RNA reads mapped to the PAN (T1.1) mRNA transcript in some KS tumors (Figs 2 and 3; ex. KS tumors 011_D and 023_B). A UCDS feature targeting the PAN RNA was used to quantitate the RNA reads mapping to PAN. While transcript levels reached nearly 300,000 TPM in several KS samples, with a median of 37,413 TPM, significant variation was observed across the KS tumors (Fig 9A and 9D; S4 Table). The PAN transcript overlaps the longer T6.1 transcript encoding ORFK7 (Fig 9B). A UCDS feature was developed to target the ORFK7 transcript upstream of PAN (Fig 9C) to avoid overlap issues in which high levels of ORFK7 expression were observed in many previous studies that were actually due to PAN. While minimal expression of ORFK7 was detected, with a median of 267 TPM, a strong correlation was observed between the expression levels of ORFK7 and PAN in the KS tumors (R = 0.6401, P<0.0001). Using UCDS features, moderate levels of reads mapping to ORFK2 and ORFK5 were consistently detected in the vast majority of the KS tumors (median = 10,138 and 4,373 TPM, respectively) (Fig 9D; bracketed in red). Quantitative analysis of RNA reads mapping to the other KSHV genes in this region revealed 10 to 100-fold lower levels of transcripts with high variability across the 34 KS tumors (Fig 9D and S4 Table).

### Hierarchical clustering analysis reveals three distinct groups of KS tumors with common patterns of gene expression

**Fig 10:**
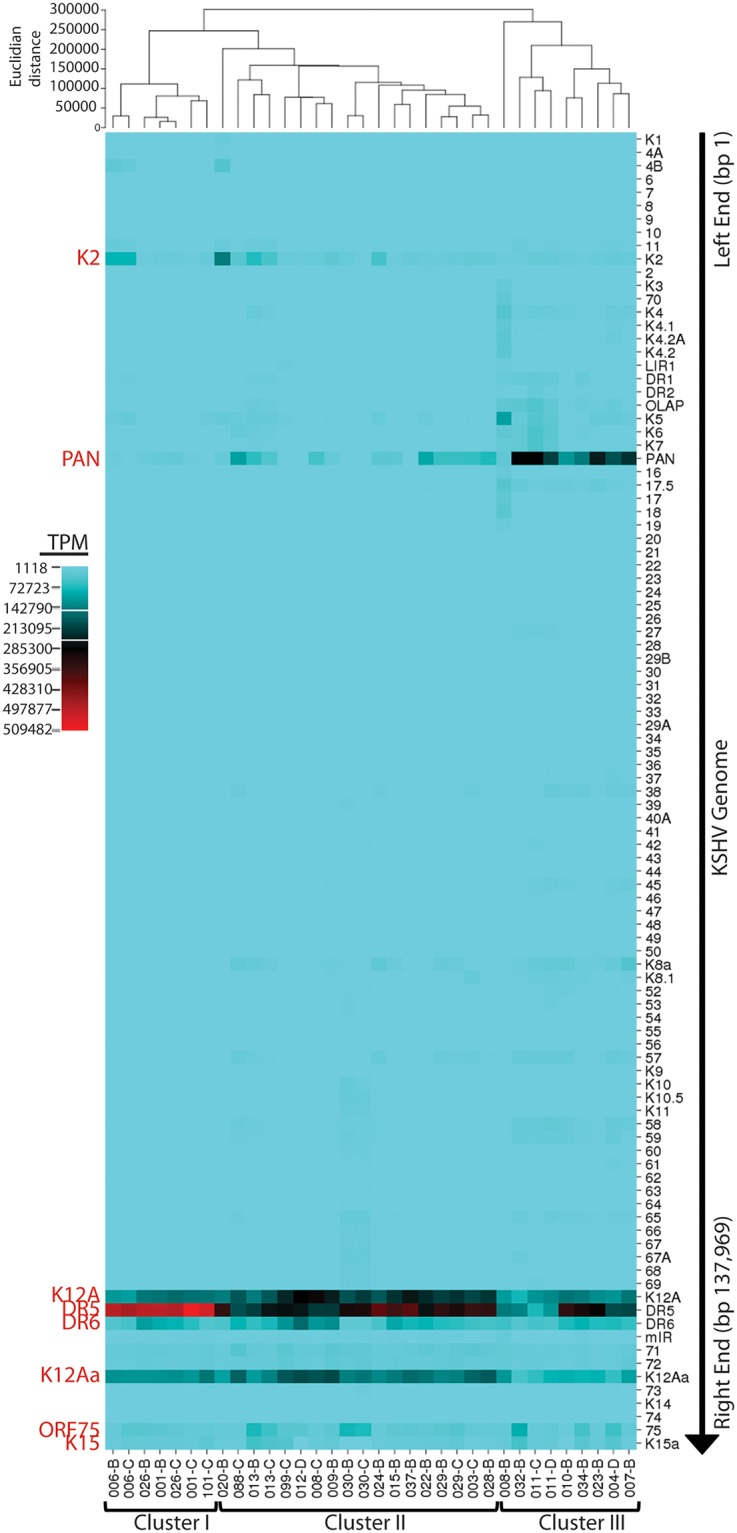
Hierarchical clustering of KSHV transcripts—“Equal width” binning. Hierarchical clustering using CIMminer was applied to KSHV gene expression data obtained by mapping RNA reads to UCDS gene features from 34 KS tumors with total KSHV-mapped reads greater than 1,000. Reads mapping to each UCDS feature were quantitated, normalized (TPM) and the expression values were color-coded for display in the cluster maps using the “equal width” binning method, as described in the text. The unsupervised clustering is shown at the top and the different KS tumor samples are indicated at the bottom of the map. The UCDS features are listed at the right side in the linear order of the KSHV genome from left (top) to right (bottom).

The transcript levels corresponding to each UCDS feature across the KSHV genome (described previously [25]) were compared for 34 of the KS tumor samples using a hierarchical clustering algorithm implemented in CIMminer [53]. This analysis was limited to the KS tumor samples with more than 1,000 total KSHV-mapped reads. The initial analysis showed expression of the genes in their order within the KSHV genome using the “equal width” binning method to map the TPM expression. This method divides the weightrange of data values into equal width intervals and each interval is mapped to one color for display in the clustered image map (Fig 10; left end genome-top, right end genome-bottom). Obvious high levels of transcripts were detected at the right end of the genome in the latency region, mapping to UCDS features for ORFK12 (UCDS-K12A), the adjacent direct repeat regions DR5 (UCDS-DR5) and DR6 (UCDS-DR6), the P3-Exon1 flanking ORF72 (UCDS-K12Aa), and the terminal ORF75 (UCDS-75) and ORFK15 (UCDS-K15a) (Fig 10). Elevated levels of PAN and ORFK2 transcripts were detected in some of the tumors at the left end of the genome. The hierarchical clustering analysis identified three clusters of KS tumors. Cluster I tumors displayed high level expression of transcripts containing the DR6 repeat region and minimal expression of PAN, while Cluster III tumors displayed high level expression of PAN and lower levels of expression of DR6 (Fig 10). Cluster II tumors were intermediate.

**Fig 11:**
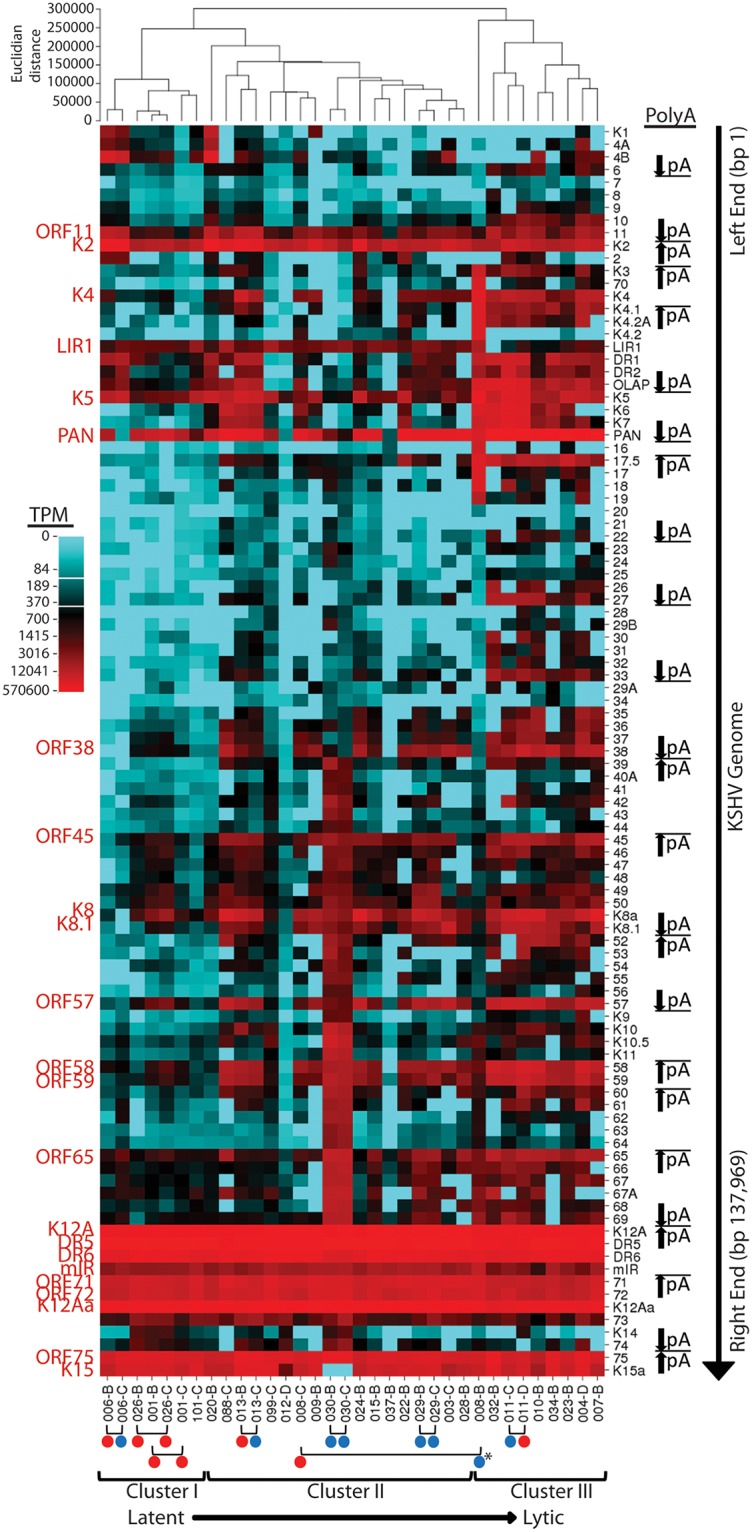
Hierarchical clustering of KSHV transcripts—“Quantile” binning. The hierarchical clustering of KSHV gene expression using CIMminer shown in Fig 10 was also performed using the “quantile” binning method, as described in the text. The position of poly-A transcription termination sites for polycistronic ORFS and the direction of transcription is indicated. The pairs of tumor biopsies from the same individual are shown at the bottom (red dot—macular tumor; blue dot—nodular tumor). The 008_B tumor sample with the unusual genomic rearrangement is indicated with a (*). The gradient of latent to lytic gene expression observed in these tumors is shown at the bottom.

A second analysis was performed using the “quantile” binning method which divides the weightrange of expression data values into intervals each with approximately the same number of data points, spreading out the color differences in the image map. The KS tumors in Cluster I displayed low levels of gene expression throughout the KSHV genome outside of the latency region (Fig 11). In contrast, tumors in Cluster III showed moderate levels of lytic gene expression throughout the KSHV genome. Abnormally high expression of the genomic region ORFK3 to ORF19 was clearly observed in the KS tumor 008_B in this cluster. This is the region that was translocated downstream of the P3(LTd) latency promoter in this tumor. An obvious block of expressed genes was observed in the Cluster III tumors between the long-inverted repeat (LIR1) and PAN at the left end of the genome (Fig 11, top). The Cluster II tumors were intermediate with elevated gene expression across the majority of the KSHV genome. Both of the KS tumors from patient 030 showed unusually high expression of the genomic region from ORF39 through the right end of the genome. This patient was the only one to carry a KSHV strain with a K15 M-allele (Fig 4), as shown by the lack of reads mapping to the K15 UCDS.

### Quantitative analysis reveals variable gene expression across the KSHV genome with higher levels of reads mapping to ORFs adjacent to poly-A sites in polycistronic loci

The hierarchical clustering analysis with quantile binning showed an obvious pattern of transcription including expression of transcripts detected by UCDS features for ORFs K11, K2, K4, LIR1, K5, PAN, 38, 45, K8, K8.1, 57, 58, 59, and 65, in addition to latency transcripts K12A, DR5, DR6, miR, 71, 72 and K12Aa (Fig 11, labeled with red text). Other genes, such as ORFs 17.5, 27, 33 and 69 were expressed in a subset of tumors. Higher levels of reads mapped to genes that flanked poly-A transcription termination sites (Fig 11, indicated at right). Most of these genes are present in loci with bicistronic or polycistronic transcripts that terminate at the same site. Thus, the high level of reads mapping to the ORFs adjacent to the poly-A site, in most cases, is due to overlapping polycistronic transcripts that contain the sequences encoding the poly-A-flanking ORF, as we have shown previously [25]. Thus, the high levels of reads mapping to these poly-A-flanking ORFs would not correlate directly with their CAP-dependent protein expression.

### No association is observed between gene expression levels and KS tumor morphotypes

The cutaneous KS tumor biopsies had been collected to represent different morphologies, sometimes from the same patient, including macular tumors with a flat appearance, nodular tumors with a distinct 3-dimensional structure and fungating tumors showing ulcerations and necrosis [54]. No correlations were observed between the morphological state and the level of total KSHV-mapped reads (Fig 1B), and the hierarchical clustering analysis revealed no obvious correlations between the morphological state and the patterns of gene expression (Fig 11). Cluster I was composed of 7 different KS tumors (5 macular; 1 nodular; 1 fungating) from 4 different individuals with a median read count of 38,605 (range 15,978–134,856). Cluster II was composed of 18 KS tumors (10 macular; 8 nodular) from 15 different individuals with a median read count of 25,538 (range 2,303–158,924). Cluster III was composed of 9 KS tumors (5 macular; 3 nodular; 1 fungating) from 8 different individuals with a median read count of 3,136 (range 2,548–146,773). Surprisingly, we observed a strong correlation between the gene expression patterns of KS tumor samples from the same patient, regardless of morphotype (Fig 11, bottom; red dot = macular, blue dot = nodular). Seven of 8 paired samples from the same individuals showed clustered gene expression profiles. The only paired sample that did not cluster was from individual 008. The 008_B sample in this pair (Fig 11, indicated with an asterisk at the bottom) exhibited the unusual high-level expression of the region between ORFK3 and ORF19, which was due to the translocation not present in the paired 008_C sample. Gene expression in the translocated region was mainly driven by the P3(LTd) promoter in the latency region, rather than by the normal gene specific promoters.

**Fig 12:**
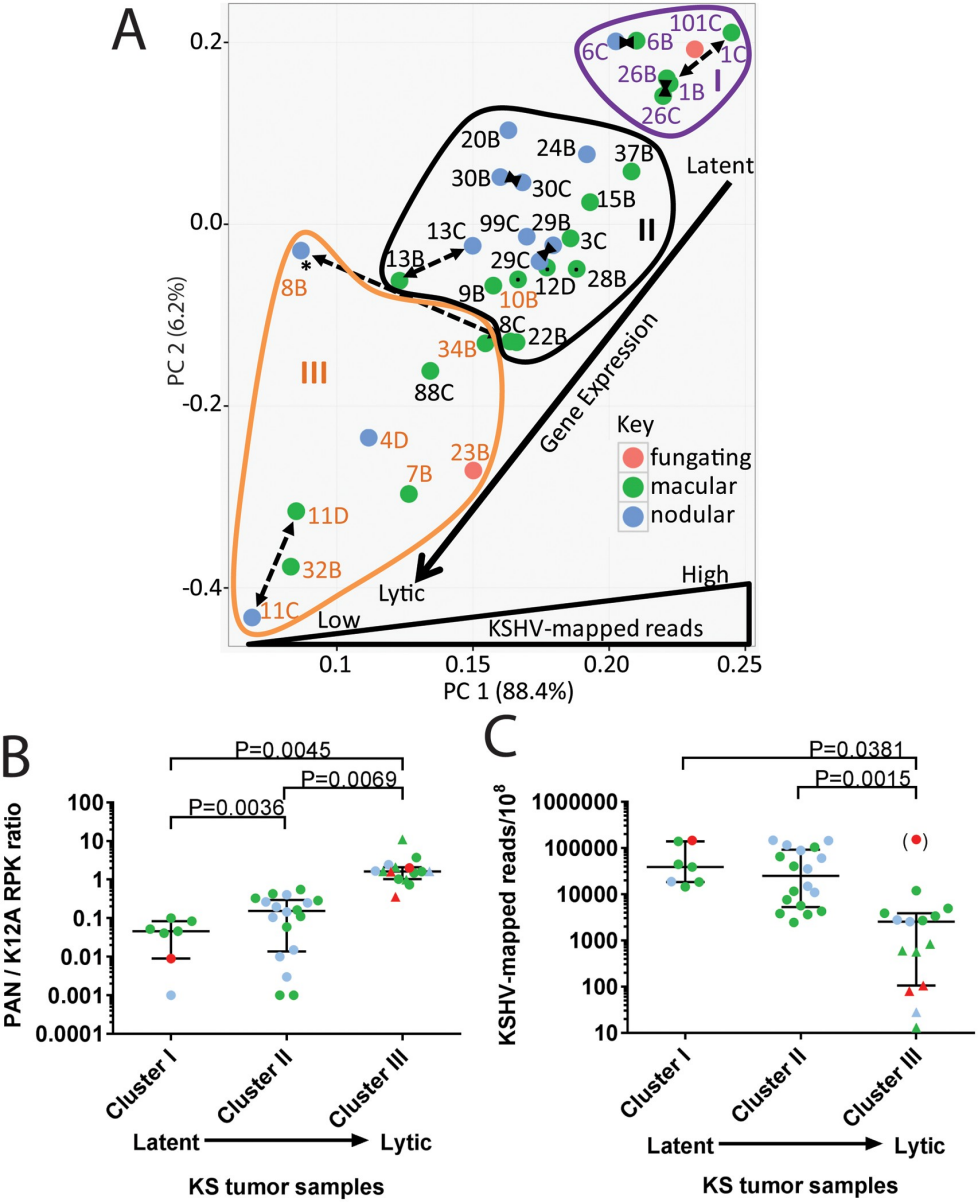
PCA analysis of normalized KSHV gene expression. A) the normalized KSHV transcript levels from the 34 KS tumors analyzed in Fig 11 were analyzed by principal component analysis (PCA). The individual KS tumor samples are color coded for morphological type. The gene expression clusters identified in Fig 11 are shown (Group I-blue text and outline; Group II-black text and outline; Group III-orange text and outline). Two tumors 10B and 88C fall outside the PCA clusters, as indicated. The paired KS tumors from the same individual are shown with a black dashed line. B) the RNA reads mapping to the PAN and K12A UCDS features targeting transcripts containing PAN and the K12 Kaposin latency region transcripts, respectively, were normalized to the length of the UCDS feature, expressed as reads per kilobase (RPK), and the ratio of PAN to K12A was plotted for the 34 KS tumors with greater than 1,000 total KSHV-mapped reads (circular dots) and 7 KS tumors with KSHV-mapped reads less than 1,000 (triangles). C) the total KSHV-mapped reads detected in the RNAseq libraries were normalized (KSHV-mapped reads/100 million total reads) and compared between the KS tumors in the gene expression clusters identified in Fig 11: (34 KS tumors, reads > 1,000; circular dots) and the 7 KS tumors with less than 1,000 mapped reads (triangles), which were grouped within the lytic Cluster III due to the high PAN/K12A ratio identified in Panel B. The morphological types are color coded: red-fungating, green-macular, blue-nodular. The median and interquartile range are shown and the gradient from latent to lytic gene expression determined from Fig 11 is indicated. Significance was determined using the unpaired T-test. The 023_B KS sample contained a very high level of KSHV-mapped reads, and due to the unusual fungating morphology of this tumor, the data was plotted (in parenthesis) but not included in the statistical calculations.

Principal component analysis (PCA) was used to reduce the complexity of the expression data from the 34 KS tumors. Principal components 1 (PC1) and 2 (PC2) captured 88.4% and 6.2% of the variation in the gene expression data, respectively. This analysis confirmed the grouping of gene expression detected by hierarchical clustering, where the tumor samples are indicated in blue text (Cluster I), black text (Cluster II) and orange text (Cluster III) (Fig 12A). As seen in the hierarchical clustering analysis, no similarities were detected between the patterns of gene expression and tumor morphotype in the PCA analysis (Fig 12A: morphotype of each tumor is color-coded). The PCA analysis revealed a gradient of KSHV gene expression from the more latent phenotype in Cluster I (upper right- hand quadrant) to the more lytic phenotype in Cluster III (lower left-hand quadrant). The similarity in the pattern of gene expression between the paired tumor samples from the same individual was confirmed, with the exception of sample 008_B (labeled with *), as discussed above (Fig 12A).

Since PAN expression is considered a marker of lytic activation, we compared PAN expression with the expression of the major T1.7A latency transcript. The RNA reads mapping to the PAN UCDS feature (size = 1.126 Kb) were compared to those mapping to the K12A UCDS feature (size = 0.183 Kb), which detects the Kaposin region in the major T1.7A latency transcript and overlapping transcripts. The reads were normalized to compare transcript levels using reads per kilobase (RPK), plotted as circular dots using the color code for morphotypes (Fig 12B). Because read data for these two features were present in all 41 of the KS tumor samples, we also compared the PAN/K12A ratio of the 7 KS tumor samples with less than 1,000 total KSHV mapped reads that were excluded in earlier expression analysis (plotted as triangles). Although the total number of KSHV-mapped reads was low in these samples, they showed an expression pattern that mirrored those seen in the lytic Cluster III (S1 Fig). The tumor sample 008_B with the altered genome was excluded. The three expression clusters showed statistically significant differences in the PAN/K12A ratio with median ratios of 0.046 (Cluster I; IQR = 0.009:0.084), 0.1545 (Cluster II; IQR = 0.0138:0.297) and 1.635 (Cluster III; IQR = 1.026:2.092) (Fig 12B), indicating that the PAN/K12A ratio could be used to distinguish the different tumor clusters. Notably, three of the four fungating samples showed high PAN/K12A ratios, compatible with a lytic phenotype.

The number of KSHV-mapped RNA reads present in each tumor sample was compared between tumors in the different expression clusters. KSHV-mapped reads were normalized to the total read depth of each tumor library, indicated as KSHV-mapped reads per 100 million library reads. The 7 KS tumors with less than 1,000 total KSHV-mapped reds were included in lytic Cluster III (triangles) due to the similarities in the PAN/K12A RPK ratios with the Cluster III tumors and the presence of lytic gene expression (S1 Fig). Both the latency Cluster I and intermediate Cluster II tumors showed high levels of KSHV-mapped reads in the tumor libraries (Cluster I median = 38,995, IQR = 18,393:139,027; Cluster II median = 25,059, IQR = 5,307:92,593). The lytic Cluster III tumors showed 23 and 10-fold lower levels of KSHV-mapped reads, respectively (Cluster III median = 1,694, IQR = 99:3,490) (Fig 12C). While the extremely high level of KSHV-mapped reads for the fungating tumor 023B (red dot) is shown in parentheses, it was excluded from the column analysis. The presence of high levels of KSHV-mapped reads in the KS tumors with latent phenotypes and the absence of KSHV-mapped reads in the KS tumors with lytic phenotypes is summarized graphically in Fig 12A.

### Correlation of gene-gene expression

**Fig 13:**
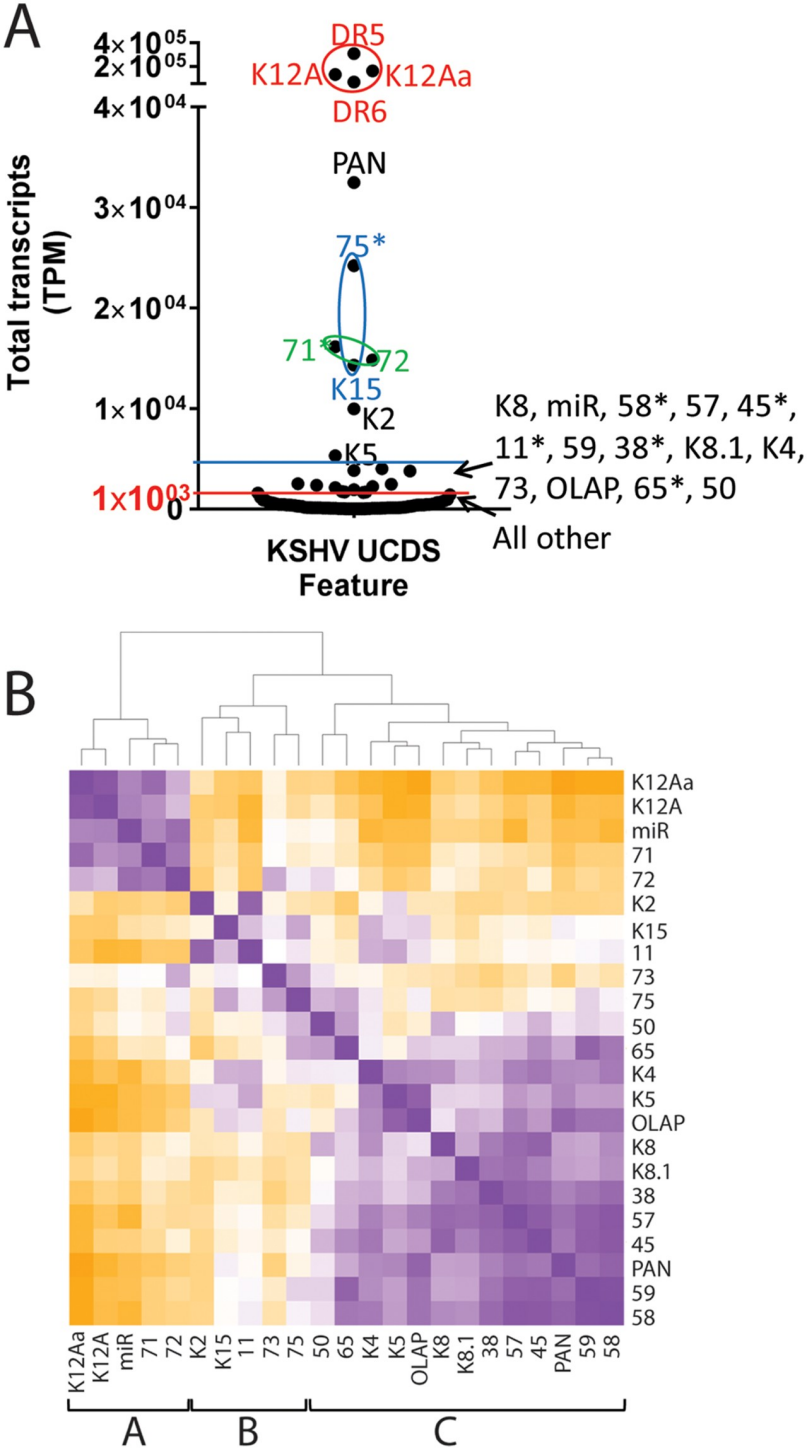
Analysis of the highly expressed genes in the KS tumors. A) The median expression levels of the most highly expressed KSHV genes were compared, with each dot representing the normalized transcript level (TPM) detected by each KSHV UCDS feature. Genes with transcript levels above 5,000 TPM (blue line) are specifically indicated. Genes with transcript levels between 1,000 (red line) and 5,000 TPM are listed together. Genes with transcript levels below 1,000 are grouped as “all other”. Clusters of UCDS features detecting common transcripts are indicated: Red—major T1.7A transcript in the latency region; Blue—K15/ORF75 bicistronic transcript; Green—ORF71/72 bicistronic transcript. An asterisk marks the genes proximal to poly-A sites. B) hierarchical clustering of gene-gene expression correlations for those genes with median transcript levels above 1,000 TPM. Pearson correlation coefficients (R) were calculated for pairs of genes using normalized transcript levels. The negative correlation (R = -1.0) is shown in orange and the positive correlation (R = 1.0) in purple, with the absence of correlation (R = 0) in white. The unsupervised hierarchical clustering is indicated at the top and the major clusters of genes (A-C) are indicated.

A set of genes that were highly expressed across the set of 34 KS tumors was identified. Median transcript levels above 5,000 TPM were observed for 11 UCDS features targeting the latency region T1.7A (Kaposin A/B/C) spliced transcript and the ORF72/71 and ORF-K15/75 bicistronic transcripts, as well as the lytic region transcripts for PAN, K2 and K5 (Fig 13A). Median transcript levels of 1–5,000 TPM were observed for 14 other UCDS features targeting ORFs 11, OLAP, K4, 38, 45, 50, K8, K81, 57, 58, 59, 63, miR and 73 (Fig 13A). To identify biologically relevant KSHV gene regulation modules, the Pearson correlation was determined for each pair of highly expressed genes. The analysis was limited to the highly expressed genes as the read level across most of the other genes was low and variable. The 008_B tumor, which showed the unusual genomic translocation, was not included. Hierarchical clustering revealed groups of co-expressed genes that showed similar expression correlation profiles across the 33 KS tumor, indicated by cohesive purple squares along the diagonal (Fig 13B). These clusters of genes represent modules undergoing similar regulation of gene expression in the tumors. The orange areas showed clusters of genes that were negatively correlated. A strong correlated expression of the different transcripts derived from the latency P3(LTd) promoter was observed in Cluster A, including the spliced Kaposin A/B/C T1.7A transcript (detected with the K12A and K12Aa UCDS features), the unspliced bicistronic ORF71/72 T1.7B transcript (detected with the ORF 71 and 72 UCDS features), and the unspliced T6.5A transcript (detected with the miR UCDS feature (see Fig 5 for transcript details). The large Cluster C contains a number of co-regulated genes implicated in the lytic replication cycle, including genes involved in regulation (ORFs 45, 50, K8, 57, and PAN), immune modulation (ORFs K4, K5), replication (ORF59) and virion structure (ORFs 38, K8.1, 65). Other genes in Cluster C with unknown function include ORF58 and OLAP [55]. The expression of these genes is positively correlated with the expression of other genes in Cluster C, but negatively correlated with the expression of the other highly expressed genes. Cluster B contained a number of genes showing variable co-regulation, including K2 (vIL-6), ORF11, K15, ORF73 and ORF75. The expression of these genes negatively correlated with the expression of the P3 (LTd) transcripts in Cluster A and the lytic replication-associated transcripts in Cluster C.

## Discussion

Our study is the first to use RNAseq analysis to quantitatively evaluate KSHV transcription at a detailed individual gene level in *in vivo* KS tumors. Our analysis showed that the most highly and consistently expressed transcripts in the Ugandan KS tumors were derived from the downstream latency transcript promoter LTd flanking ORF72 [41, 49], herein designated as the third latency promoter—P3(LTd). Very high levels of reads mapped to each of the UCDS features for K12A(Kaposin), K12Aa(P3-Exon1) and the direct repeats DR5 and DR6 within the latency region of the genome. These features target different regions of a 1.7 Kb spliced transcript from the P3(LTd) promoter (herein referred to as T1.7A)[41, 49]. The read depths of the K12Aa (P3-Exon1) and K12A(Kaposin) features, which target the 5’ and 3’ ends of the T1.7A transcript, respectively, are quite similar, while the read depths of the DR5 and DR6 features were more variable, due to length of the UCDS feature, GC-content, sequence repeats in the DR5 region, sequence variability in the DR6 region and mismatches between RNA reads of the KS tumors and the KSHV GK18 reference sequence used for mapping. The presence of a single set of closely linked poly-A transcription termination signals in the region downstream of ORFK12 [52, 56], the strong correlation between the expression levels of the different regions of the T1.7A transcript in the different KS tumors, and the correspondingly high level of split reads mapping across the splice junction between upstream P3-exon1 splice donor and the downstream DR6 splice acceptor together support the conclusion that the P3(LTd) spliced T1.7A transcript is the most highly expressed transcript in the Ugandan KS tumors and was confirmed by RT-PCR analysis.

Early in situ studies of KS tumor lesions detected hybridization of a probe derived from the 0.7 Kb transcript encoding ORFK12 in every KS tumor examined [13, 14, 57–59]. Subsequent PCR and Northern analyses in cultured PEL cells indicated that transcripts hybridizing to the 0.7 Kb probe were almost always larger than 0.7 Kb, initiating from a promoter (P4) upstream of the GC-rich DR6 repeat region [44] (herein referred to as transcript T1.5A) or from the P3(LTd) promoter upstream of ORF72 (Transcript T1.7A) [41, 49]. The T0.7, T1.5A and T1.7A transcripts all terminate within a set of closely spaced poly-A termination signals (Group A). Our quantitative RNAseq and RT-PCR data provided strong evidence that the vast majority of transcripts hybridizing to the 0.7 Kb probe in KS tumors correspond to the spliced T1.7A transcript from the P3(LTd) promoter.

Previous In situ hybridization studies also detected strong signals with probes specific for the ORF71 and ORF72 latency genes in the majority of spindle cells in KS tumors [59, 60]. Subsequent analysis of latently-infected PEL cells identified three transcripts encoding ORFs 71 and 72, which are derived from the constitutive P1(LTc) promoter upstream of ORF73 and co-terminate at the poly-A site downstream of ORF71 (Group B transcripts)[6]. These transcripts include a full length 5.7 Kb unspliced tricistronic transcript (T5.7B in this study) encoding ORFs 73, 72 and 71, and three spliced transcripts, each containing the same short 5’ exon downstream of the P1(LTc) promoter that is alternatively spliced to either ORF73 producing large 5.2 and 5.4 Kb tricistronic ORF73/72/71 transcripts (T5.2B and T5.4B in this study) or ORF72 producing a 1.8 Kb bicistronic ORF72/71 transcript (T1.8B in this study). Although the tricistronic ORF73/72/71 transcripts have been detected in PEL cells in vitro using an ORF73 probe, in situ hybridization signals have not been observed in KS lesions in vivo, indicating the low abundance of tricistronic ORF73/72/71 transcripts encoding ORF73 LANA [61]. Our RNAseq analysis confirmed this observation as very few RNAseq reads from the KS tumors mapped to any of the tricistronic ORF73/72/71 transcripts and very few split RNA reads identified the splicing events downstream of the P1(LTc) promoter. Furthermore, no triscistronic transcripts were detected by RT-PCR analysis of the KS tumor RNAs. As the tricistronic ORF73/72/71 transcripts are the only transcripts known to encode the latency associated nuclear antigen ORF73/LANA, which is ubiquitously present in KSHV-infected KS spindle cells, the LANA protein appears to be more stable than its mRNA transcript. Thus, while the LTc promoter is constitutive in KSHV-infected PEL cells, its activity in the KS tumors appears to be quite low, regardless of morphotype or activation state.

We detected moderate and consistent levels of reads mapping to the ORF71 and ORF72 UCDS features in all the KS tumors, confirming the previous in situ hybridization studies. The RNA mapping and RT-PCR data indicate that the majority of these reads were derived from an unspliced bicistronic transcript encoding ORF72 at the 5’ end and ORF71 at the 3’ end (herein referred to as T1.7B), which has been previously characterized in cultured PEL cells [49, 50]. Like the spliced T1.7A transcript described above, the unspliced T1.7B transcript is transcribed from the P3(LTd) promoter but terminates at the poly-A site downstream of ORF71. A small upstream uORF in the initial 5’ region of this transcript has been shown to downregulate ORF72 expression, and a downstream IRES site facilitates expression of ORF71, resulting in coordinated expression of both ORF71 and ORF72 in PEL cells [43, 62]. As the unspliced T1.7B and spliced T1.7A transcripts are produced from the same P3(LTd) promoter, they both contain the same 5’ region encoding the small uORF upstream of ORF72, herein called P3exon1. A very high level of RNA reads mapped to the K12Aa UCDS feature that targets the P3exon1 region of both transcripts. The expression level of the bicistronic unspliced T1.7B transcript detected with the ORF71 and ORF72 UCDS features was only 10% of the expression level of the shared 5’ P3exon1, indicating that 90% of the P3(LTd) transcripts bypass the termination signal after ORF71 producing long initial pre-mRNA species, which terminate downstream of ORFK12 (the unspliced transcript is referred to herein as T6.5A). The majority of this mRNA is processed to remove the intron containing ORFs 72, 71 and microRNAs by splicing, generating the spliced T1.7A transcript described above. Read data mapping to the miR UCDS feature targeting the intronic region indicated that < 5% of the P3(LTd) transcripts correspond to the unspliced T6.5A precursor transcript.

A previous study suggested that the transcripts hybridizing to ORF71 and ORF72 probes in KS lesions were bicistronic spliced transcripts derived from the upstream P1 (LTc) promoter [6], as has been observed in PEL cells. TopHat2 analysis of RNA reads in our study detected little evidence for splicing of transcripts from the P1(LTc) promoter, and essentially no expression of the 5’ exon downstream of the P1(LTc) promoter. Thus, our RNA-seq data indicates that the transcripts encoding ORF72 and ORF71 in KS lesions are derived from the P3(LTd) promoter, not the P1(LTc) promoter. Note that the spliced T1.8B bicistronic ORF72/71 transcript from the P1(LTc) promoter and the unspliced T1.7B bicistronic ORF72/71 transcripts from the downstream P3(LTd) promoter are of similar size and could have been mistaken for each other by Northern analysis in previous studies. While the majority of transcripts detected using the ORF71 and ORF72 UCDS features appeared to be bicistronic, confirming previous data from PEL cells, our analysis provided evidence for expression in some tumors of monocistronic ORF71 (T0.9B) and ORF72 (T1.0C) transcripts from the P3(LTd) promoter that have been detected previously [42, 43].

Variable numbers of repeats and sequence heterogeneity in DR5/DR6 repeat region have been detected previously in other KSHV strains, which alter protein translation from the T1.7A mRNA [41]. Our RNAseq analysis revealed two nucleotide deletions in the overlapping T1.7A and T1.5 transcripts that are conserved in the Ugandan KSHV strains, both of which would alter the open reading frames in these transcripts from those described previously in BCBL-1 and other KSHV strains. In eleven Uganda KSHV strains typified by the Zambian KSHV strain ZM114, the putative protein products of the T1.5/T1.7A transcripts would be a BCBL-1 Kaposin B homolog and a novel protein, herein called Kaposin E, that would initiate with the same CUG codon as BCBL-1 Kaposin C but lack the fusion with Kaposin A. In fifteen other Uganda KSHV strains, typified by the Zambian KSHV strain ZM004, additional nucleotide changes in the T1.5A/T1.7A transcripts eliminate the CUG-initiated Kaposin B and C ORFs leaving Kaposin A as the only predicted translational product of this transcript. While other non-AUG translation initiation could be utilized in these KSHV strains, it is not clear what protein products would be translated. Due to the limited amount of RNA template in our biopsies and the repetitive nature of the sequence and the high GC content (in some regions >95%), we were unable to PCR amplify T1.7A transcripts from these or other KSHV strains to determine the exact nucleotide sequence. Since translation products from this mRNA could be the major KSHV-encoded proteins in the KS tumors, identification and functional characterization of the T1.7A-encoded proteins will be critical for understanding the role of KSHV in the KS tumors.

While the exact coding potential for the T1.7A transcripts in the Ugandan KSHV strains awaits functional analysis, it is clear that splicing of this transcript liberates a 4.8 Kb intron, which is the substrate for processing of the majority of the KSHV microRNAs [49]. Thus, a major outcome of the expression of the T1.7A transcript would be the production of this set of KSHV microRNAs [49]. Additional transcripts from the P1(LTc) promoter upstream of ORF73, including T1.8A and T1.6A, also generate the region encoding these microRNAs as an intron during splicing. However, we observed very low and variable expression of the P1(LTc) transcripts in the KS tumors, indicating that splicing of the T1.7A transcript from the P3(LTd) would generate the vast majority of processed microRNAs in the tumors. In addition to encoding the Kaposin A ORF downstream of the DR5/DR6 repetitive sequences, the T1.7A transcript is a precursor of the miR-K10a and miR-K12 microRNAs, which are embedded within the 3’ end of the transcript [46]. Thus, the RNAseq data indicates that a major functional role of P3(LTd) promoter in the establishment and progression of KS tumors would be to generate the precursor substrates for all the KSHV microRNAs. The P3(LTd) promoter has been shown to be constitutively active in PEL cells, and it is not clear whether its activity is increased by reactivation using RTA [41, 49, 50].

Unexpectedly, we identified one KS tumor in a sampling of paired tumors from individual 008, which contained a translocation of a 14 Kb region of the left end of the KSHV genome flanking the long-inverted repeat LIR1 into the LIR2 repeat within the latency region at the right end of the genome. This translocation is located downstream of the highly active P3(LTd) latency promoter, resulting in numerous novel transcripts from the P3(LTd) promoter containing the initial P3-Exon1 spliced to acceptor sites in the translocated K3, K5, and ORF17 genes. This translocation did not appear to inhibit the splicing of the highly expressed T1.7A transcript encoding the Kaposin A/B/C complex, even though the excised intron was 14 Kb longer. A spliced transcript containing the P3-Exon1 spliced to ORFK5 was the most highly expressed transcript in the 008_B tumor. Since the P3-Exon1 encodes the regulatory uORF, described above, the translation of protein products from the highly expressed novel spliced transcripts in this tumor could be downregulated. The majority of highly expressed genes in the translocated region were transcribed right to left in the same orientation as the transcripts from the P3(LTd) promoter from the same DNA strand. While elevated levels of reads also mapped to other genes in the translocated region, such as PAN, OLAP, and ORF18, these genes would have been transcribed from their proper promoters on the other strand in the opposite direction. As this tumor was not analyzed using a stranded RNA library, it was unclear whether the RNA reads mapped to PAN, OLAP and ORF18 transcripts from the opposite strand. No other KS tumor exhibited this translocation suggesting that the translocation was a unique event in the 008_B tumor. However, the existence of this translocation and the resulting altered transcription pattern indicates the plasticity of the KSHV genome and the strong role of the P3(LTd) promoter in the development of KS.

Using a PAN-specific UCDS gene feature, we detected expression of the PAN RNA in most of the 34 Ugandan KS tumors. Although the median expression level for PAN was high, it was 5-fold less than the expression level of the K12A (T0.7) region. The range of PAN expression levels was large, ranging from barely detectable in some tumors to extremely high in other tumors, with expression levels higher than K12A(T0.7) in 7/34 KS tumors. While previous in situ hybridization studies identified specific cells expressing PAN, our RNAseq analysis only detected the average expression level within the biopsied lesion. The expression of PAN correlated positively with the expression of a large number of lytic cycle genes, including K6 (vMIP-1), ORF59, K8 (bZIP), ORF25 (MCP), ORF26 (VP23), and ORF65 (SCP), which were previously detected by in situ hybridization in limited numbers of KS spindle cells. Only a weak correlation was detected between the expression of these lytic cycle genes and ORF50 (RTA). There was no correlation between the expression of these lytic genes and genes, including K2(vIL-6), ORF16(vBCL-2), K10 (vIRF-4), or K11 (vIRF-2), whose expression has also been observed infrequently in KS spindle cells. Surprisingly, the RNA-seq data showed a strong correlation between the expression of K2 (vIL-6) and the genes for the ORF11 tegument and the ORF4 complement control (KCP) proteins, indicating a common regulation of gene expression that was not shared with PAN or the other lytic genes. This data suggests that the expression of discrete subgroups of genes in the lytic pathway can be differentially regulated in the tumors.

A recent study by Tso et al. used RNAseq to analyze KS lesions from four HIV-infected individuals from Zambia and Tanzania that were undergoing anti-retroviral therapy [26]. These RNA samples were sequenced to a depth of 10–13 million total reads obtaining 718, 1,650, 3,441 and 17,202 reads (median = 2,545) mapping to the NC_009333 KSHV reference sequence. No obvious pattern of KSHV expression was reported across the four samples, presumably due to the limited sample size and variable KSHV read depths of the different biopsies. High levels of latency gene transcripts were reported, including ORF73 LANA, as well as an increased expression of viral immune modulation genes, including ORF-K2 (viral interleukin-6), ORF-K5 (modulator of immune recognition), ORF-K7 (viral inhibitor of apoptosis) and ORF75 (degradation of ND10 protein). In contrast, we analyzed RNA libraries from 41 KS biopsies at a 10-fold greater depth with a 5-fold greater depth of KSHV-mapped reads (median = 10,232; maximum = 158,924). The increased sample size and depth of sequencing allowed for clear patterns of KSHV expression to be detected in the panel of KS tumors from anti-retroviral therapy-naïve Ugandans in our study. We detected high levels of ORF75 and ORFK15 transcripts in all the KS biopsies, but only variable levels of ORFK2 and ORFK5 transcripts. We determined that 76% of the RNA reads mapping to ORF75 are derived from K15/ORF75 bicistronic transcripts, as K15 transcripts terminate downstream of ORF75 [52]. Since it is not known whether ORF75 protein is translated from the bicistronic transcripts through alternate initiation pathways, the biological relevance of the bicistronic transcripts to ORF75 function is unclear. While ORFK15 protein is abundantly expressed in KS tumor biopsies where it is believed contribute to the invasiveness and angiogenic properties of the tumor cells [34, 35], we have identified no reports of ORF75 tumor expression. Even though transcripts of both ORFK15 and ORF75 are highly expressed in the most latent KS tumors, both genes are required for viral lytic replication in vitro [32, 63], suggesting important pleiotrophic effects in the virus life cycle.

As we have indicated previously, mapping of RNAseq reads to the regions of the KSHV genome encoding entire ORFs, as performed in the Tso et al study, is problematic due to the compact size of the KSHV genome and overlapping nature of the KSHV transcripts [25]. While Tso et al detected high-level expression of ORF73 LANA by mapping reads to the entire ORF, we detected only minimal expression of ORF73 using UCDS features that avoided the large repetitive sequence within LANA. This raises the question whether the ORF73 expression in the Tso study could have been due to multiple mapping of reads to repetitive sequences. Our mapping protocol limited reads to a single mapping event to avoid this problem. As discussed above, a large T6.1 transcript derived from the positive strand of the KSHV genome encoding ORFK7 overlaps with the PAN RNA derived from the same DNA strand and ORFK5 transcripts from the opposite strand (see Fig 9B). Using the UCDS approach with non-overlapping PAN and ORFK7 UCDS features, we determined that the high-level expression of ORFK7 detected using the typical mapping to the NC_009333 ORFs is due to high levels of PAN transcripts, not ORFK7. With the UCDS approach, we detected high levels of PAN in some tumors with very low levels of ORFK7 transcripts in all tumors. Tso et al did not report PAN transcript levels as PAN is not an ORF in the NC_009333 accession record and therefore was not quantitated.

Using deep sequencing of RNA transcripts, our study quantitated the expression level of genes across the entire KSHV genome using UCDS features, providing a complete global analysis of the KSHV transcriptome in the KS lesions from HIV-infected Ugandans that were anti-retroviral therapy naive. Unsupervised hierarchical clustering and PCA analysis of gene expression revealed three distinct tumor clusters. Cluster I consisted of 7 tumors from four individuals with a mixture of morphotypes. These tumors showed a predominant latency phenotype with low levels of gene expression outside of the latency locus with 22 ORFK12 transcripts per PAN transcript. Cluster III consisted of seventeen tumors from fourteen individuals with a mixture of morphotypes, which showed a predominant lytic phenotype with gene expression detected across the entire KSHV genome. In contrast, these tumors expressed 1.6 PAN transcripts per ORFK12 transcript. Cluster II consisted of eighteen tumors from fifteen individuals with a mixture of morphotypes, which showed elevated gene expression across the right end of the genome and limited gene expression across the left end of the genome with 6.0 ORFK12 transcripts per PAN transcript. Surprisingly, the Cluster III KS tumors with the most lytic phenotypes contained the fewest number of RNA reads mapping to the KSHV genome, while the Cluster I tumors with the most latent phenotypes contained the highest number of KSHV-mapped RNA reads. This is in direct contrast to what was expected from previous studies on KSHV infection in vitro, where reactivation of latent virus was shown to initiate new lytic gene transcription but not alter constitutive latent transcription [64].

Of the 41 KS tumor samples sequenced, 7 had fewer than 1,000 KSHV-mapped reads, and 3 had fewer than 100 KSHV reads although all the tumors were advanced T1 tumor stage. We found that KS tumors with 15,000 to 150,000 total KSHV-mapped reads had similar viral loads of ~0.5 KSHV genomes/cell, suggesting that KSHV gene expression did not correlate with the level of KSHV infection in cells in the lesion. While the KS samples with less than 1,000 KSHV-mapped reads were excluded from hierarchical cluster analysis of the RNAseq data due to problematic normalization and clustering, we found that these tumors had a high PAN/K12 read ratio, consistent with a lytic phenotype. Thus, all the KS tumors except for one fungating sample (23B) showed a significant correlation between lytic gene expression across the entire KSHV genome and low levels of total KSHV transcription in the KS lesion. Interestingly, previous studies using double staining of KS lesions showed that vIL6- or vGPCR-positive KS spindle cells lacked punctate nuclear LANA spots indicating a decreased expression of LANA and possibly other latency proteins during lytic reactivation [21]. In a previous PCR profiling study of KSHV expression in KS tumors from Malawi, 10 of 61 tumors were excluded from analysis due to the lack of detectable mRNA for KSHV latent genes and three additional samples were excluded because they had low levels of KSHV latent mRNA [24]. Since these samples were excluded from analysis in this study, it is not known whether they displayed a lytic phenotype, as was seen for comparable tumor types using RNAseq analysis in our study.

It was previously reported that qPCR profiling of Malawian KS tumors detected abundant transcription within the viral latency locus including ORF73(LANA), ORF72(vCyc), ORF71(vFLIP), ORFK12(Kaposin) and microRNAs [24]. This study detected a poly-A site position effect in which the PCR signal for ORF71, which is proximal to the poly-A site, was higher than the distal ORF72 or ORF73. Since these signals were believed to originate from the same tricistronic mRNA transcript, it was concluded that poly-A purification of the mRNA templates had artifactually increased the transcript levels for the genes adjacent to the poly-A site. Using our RNAseq approach, we were able to characterize the latency region transcription in KS tumors in a more granular detail. We detected minimal numbers of RNA reads mapping to LANA on the tricistronic ORF73/72/71 latency transcript of the P1 promoter and showed that the vast majority of reads mapping to ORFs 71 and 72 were actually derived from the T1.7B bicistronic transcript of the P3/LTd promoter. These findings explain the results of the Hosseinipour study and indicate that the increased expression of ORFs 71 and 72 is not an artifact of poly-A purification.

An important consideration in our study was the use of the KSHV-GK18 reference sequence (NC_009333) to map the RNA reads from the different KSHV strains in the Ugandan KS tumors. We previously developed a detailed map of the KSHV transcriptome annotated to the NC_009333 reference sequence. We also developed a detailed gene feature file (GFF) for the NC_009333 sequence with UCDS features allowing quantitation of overlapping genes in the KSHV genome [25]. Since our phylogenetic analysis revealed the presence of multiple KSHV strains in the 34 Ugandan KS tumors, we decided to use the NC_009333 sequence as a common alignment target for consistency. We observed instances of sequence mismatches between KS tumor RNA reads and the aligned NC_009333 sequence throughout the KSHV genome, however, there were only a few obvious regions where the variations in read sequence affected transcript quantitation, as seen for K15, as described above. We observed some mapping issues within the K1 gene, which is known to exhibit high levels of variation in specific regions of the gene. However, there was sufficient homology between the reads and the GK18 sequence outside of the K1 variant regions to determine K1 expression. We also observed problems mapping reads to the DR5 and DR6 repeat regions, which have shown high levels of sequence variation. To compare sequences across problematic regions, we performed additional mapping studies using published KSHV strains with variant sequences as references.

In summary, quantitative RNAseq analysis using a unique set of UCDS gene features has provided an in-depth analysis of the KSHV transcriptome in 41 T1 stage KS lesions from 30 HIV-infected ART-naïve Ugandans, yielding a unique resource for subsequent analysis of specific transcripts by other approaches. Hierarchical clustering and PCA analysis of KSHV transcripts revealed three clusters of tumors displaying a gradient of KSHV gene expression ranging from minimal gene expression outside of the latency locus (latent expression) to wide-spread gene expression across the viral genome (lytic expression). Paradoxically, the tumors with the latent phenotype had high levels of total KSHV transcription while the tumors with the lytic phenotype had low levels of total KSHV transcription. Morphologically distinct KS tumors from the same individual showed similar KSHV gene expression profiles suggesting that the tumor microenvironment and host response played a determining role in the activation level of KSHV within the infected tumor cells.

## Materials and methods

### Clinical samples

HIV infected adults with KS were recruited from the Uganda Cancer Institute (UCI)/Hutchinson Center Cancer Alliance in Kampala, Uganda. Eligible participants had to be at least 18 years of age and have late stage (T1) KS by AIDS Clinical Trials Group staging criteria and be naïve for antiretroviral therapy (ART). All KS samples were obtained with written informed consent.

### RNA-sequencing

Total nucleic acids were extracted using RLT buffer (Qiagen) and RNA was isolated using the RNeasy mini kit with DNAse treatment step. Total RNA integrity was checked using an Agilent 2200 TapeStation (Agilent Technologies, Inc., Santa Clara, CA) and quantified using a Trinean DropSense96 spectrophotometer (Caliper Life Sciences, Hopkinton, MA). Unstranded RNA-seq libraries were prepared from 300 ng of total RNA using the TruSeq RNA Sample Prep Kit v2 (Illumina, Inc., San Diego, CA, USA). Four KS tumors libraries were prepared using the TruSeq Stranded mRNA Library Kit (Illumina) from 100 ng of total RNA. Library size distributions were validated using an Agilent 2200 TapeStation (Agilent Technologies, Santa Clara, CA, USA). Additional library QC, pooling of indexed libraries, and cluster optimization was performed using Life Technologies’ Invitrogen Qubit 2.0 Fluorometer (Life Technologies-Invitrogen, Carlsbad, CA, USA). The unstranded RNA-seq libraries were pooled (5-plex) and the stranded libraries were pooled (4-plex) and each pool was clustered onto a flow cell lane. Sequencing was performed using an Illumina HiSeq 2500 in “High Output” mode with a paired-end, 50 base reads (PE50) sequencing strategy for the unstranded libraries and non-paired end for the stranded libraries. Image analysis and base calling was performed using Illumina’s Real Time Analysis v1.18 software, followed by ‘demultiplexing’ of indexed reads and generation of FASTQ files, using Illumina’s bcl2fastq Conversion Software v1.8.2.

### RT-PCR analysis

RNA from four KS tumors was analyzed by RT-PCR to detect different spliced and unspliced transcripts from the latency region of KSHV (S2 Fig). Forward PCR primers from the 3’ end of the ORF73 sequence (P1/2F, _bp124121_ 5’ CCCTGCCATTAACCCAGCCAG 3’ _bp124101_) and the P3-exon1 sequence downstream of the P3 promoter (P3F, _bp123950_ 5’ ACCCATCTACCTCAACTGAAC 3’ _bp123930_) were develop to use in conjunction with a reverse primer from the 3’ end of the ORF72 sequence (72R, _bp123757_ 5’ CGATCCTCACATAGCGTGGGA 3’ _bp123777_) to amplify a 406 bp fragment of the tricistronic ORF73/72/71 (T5.2–5.7B) transcript and a 194 bp fragment of the bicistronic ORF72/71 (T1.7B) transcript, respectively. Reverse PCR primers from the 3’ end of the K12 sequence (KapR1, _bp118037_ 5’ CGTTGCAACTCGTGTCCTGAATG 3’ _bp118059_) and the sequence spanning the exon junction of transcript T1.7A (P4R, _bp119036_ 5’ TTATAGCGTTTC 3’ _bp119047_: _bp123843_ 5’ CTGTAGAGCCTG 3’ _bp123854_), were developed to amplify 1,042 and 120 bp fragments of the T1.7A spliced transcript encoding the K12 Kaposin A/B/C region, respectively, using the P3F forward primer. A forward PCR primer from the sequence downstream of the P4 promoter flanking the DR6 repeat region (P4F, _bp 118973_ 5’ TTGGATTTACACGTATCGAGG 3’ _bp 118953_) was developed to amplify a 186 bp fragment of K12 using the KapR1 reverse primer. PCR reaction conditions were developed using BCBL-1 DNA as template. cDNA was prepared from KS tumor RNA using Tetro reverse transcriptase (Bioline) and random decamers at 45 °C in the presence of RiboSafe (Bioline), and PCR amplification was performed using MyTaq HS polymerase (Bioline) and forward and reverse primers (5.0 μM), after denaturing at 95° 2.5 minutes with 45 cycles of 95° 15 sec/60° 30 sec/ 72° 60 sec). The PCR products were visualized by electrophoresis using ethidium bromide. Reactions without reverse transcriptase were performed to control for DNA contamination.

### Data analysis

RNA reads mapping to the human genome (hg19) were removed using the Bowtie2 program (version 2.2.6) [65]. The remaining RNA reads were aligned to the KSHV reference sequence NC_009333 for the KSHV GK18 strain using TopHat2 (version 2.0.14) [66] in a local instance of Galaxy [67]. Mapped reads were visualized using the Integrated Genome Viewer (IGV; version 2.3.75) [68]. For quantitation purposes, the reads from paired-end libraries (non-stranded) were analyzed as unpaired (single-end data) to allow each read of the pair to map unambiguously to a single gene feature. The reads from both strands of the stranded libraries (non-paired end) were either concatenated and analyzed together (librarytype = unstranded) for comparison to the non-stranded paired end libraries or were analyzed separately (librarytype = FR) to show strand specificity. TopHat2 was used to detect splicing events ab initio. The default presets were used except that the maximum intron length was decreased to 10,000 and the maximum number of alignments allowed was decreased from 20 to 1, to avoid overcounting reads to repetitive regions. HTSeq (version 0.6)[69] was used to quantitate the reads mapping to the unique set of UCDS gene features within the novel revised gene feature file “KSHV NC_009333 UCDS ver 020116.GFF“(S1 File) [25]. The “intersection (non-empty)” setting in HTSeq was used to count all reads mapping completely or partially to a UCDS feature to maximize read count (featuretype = UCDS; IDattribute = gene). No reads were eliminated by ambiguity since the UCDS features were 50 bp apart, the length of a read. The read count was expressed as transcripts per million (TPM) by first normalizing the read count to reads per kilobase (RPK) by dividing the read counts by the length of the UCDS gene feature, in kilobases. The “per million” scaling factor was determined by summing all of the RPK values in a sample and dividing by 1,000,000. Each RPK value was then divided by the “per million” scaling factor to give TPM of mapped KSHV reads. Hierarchical clustering of TPM normalized expression levels was performed using the algorithm implemented in CIMminer [53]. Hierarchical clustering of the gene correlation matrix was performed by calculating the Pearson correlation between the normalized transcript levels (TPM) associated with each pair of UCDS gene features, using a script in R to create and output the correlation matrix. Shiny web applications were developed for R-based principal component analysis in Fig 12A (available at https://efg-ds.shinyapps.io/pcaApp/) and the boxplot analysis of gene expression levels in Fig 5C (available at https://efg-ds.shinyapps.io/boxplotApp/).

### Phylogenetic analysis

Phylogenetic analysis of the KSHV strains in the KS tumors was performed on the complete coding sequences of ORF75 (3,891 bp), which were assembled from the 50 bp RNA reads for 23 of the KS tumors with IGV, using maximum likelihood analysis. The sequences have been deposited in Genbank.

### Ethics statement

The biological samples in the study were obtained specifically for the study. All participants provided written informed consent. The protocol was approved by the institutional review boards at the Fred Hutchinson Cancer Research Center, the Makerere University School of Medicine, and the Uganda National Council for Science and Technology.

## Supporting information

S1 Fig KSHV transcriptome analysis in Ugandan KS tumors with <1,000 total KSHV mapped reads. (PDF)

S2 Fig Characterization of latency transcripts by RT-PCR. (PDF)

S3 Fig Gene expression in specific bicistronic loci in Ugandan KS tumors. (PDF)

S4 Fig Kaposin T1.7A transcripts and encoding potential of Ugandan KSHV strains. (PDF)

S1 Table Summary of Ugandan KS tumor samples and RNAseq libraries. (PDF)

S2 Table Summary of latency region transcripts. (PDF)

S3 Table Read counts and normalization for 41 Ugandan KS tumors. (XLS)

S4 Table Comparison of total and primary transcripts in 33 Ugandan KS tumors. (PDF)

S1 File KSHV NC_009333 UCDS ver 020116.GFF. All BAM and HTSEQ read count files for the 41 KS tumors are available at the GEO website (NIH), accession #GSE116160. ORF75 sequences were deposited at Genbank with accession numbers MH997408-MH997431.
(GFF)

## References

[1] Mariggio G, Koch S, Schulz TF. Kaposi sarcoma herpesvirus pathogenesis. Philosophical transactions of the Royal Society of London Series B, Biological sciences. 2017;372(1732). 10.1098/rstb.2016.0275 28893942 PMC5597742

[2] Russo JJ, Bohenzky RA, Chien MC, Chen J, Yan M, Maddalena D, et al Nucleotide sequence of the Kaposi sarcoma-associated herpesvirus (HHV8). Proc Natl Acad Sci U S A. 1996;93(25):14862–7. 8962146 10.1073/pnas.93.25.14862 PMC26227

[3] Nicholas J, Zong JC, Alcendor DJ, Ciufo DM, Poole LJ, Sarisky RT, et al Novel organizational features, captured cellular genes, and strain variability within the genome of KSHV/HHV8. J Natl Cancer Inst Monogr. 1998;(23):79–88. 9709308 10.1093/oxfordjournals.jncimonographs.a024179

[4] Bechtel JT, Liang Y, Hvidding J, Ganem D. Host range of Kaposi’s sarcoma-associated herpesvirus in cultured cells. J Virol. 2003;77(11):6474–81. 10.1128/JVI.77.11.6474-6481.2003 . 12743304 PMC155009

[5] Kedes DH, Lagunoff M, Renne R, Ganem D. Identification of the gene encoding the major latency-associated nuclear antigen of the Kaposi’s sarcoma-associated herpesvirus. The Journal of clinical investigation. 1997;100(10):2606–10. 10.1172/JCI119804 9366576 PMC508462

[6] Dittmer D, Lagunoff M, Renne R, Staskus K, Haase A, Ganem D. A cluster of latently expressed genes in Kaposi’s sarcoma-associated herpesvirus. J Virol. 1998;72(10):8309–15. 9733875 10.1128/jvi.72.10.8309-8315.1998 PMC110196

[7] Kellam P, Boshoff C, Whitby D, Matthews S, Weiss RA, Talbot SJ. Identification of a major latent nuclear antigen, LNA-1, in the human herpesvirus 8 genome. Journal of human virology. 1997;1(1):19–29. 10195227

[8] Talbot SJ, Weiss RA, Kellam P, Boshoff C. Transcriptional analysis of human herpesvirus-8 open reading frames 71, 72, 73, K14, and 74 in a primary effusion lymphoma cell line. Virology. 1999;257(1):84–94. 10.1006/viro.1999.9672 10208923

[9] Ciufo DM, Cannon JS, Poole LJ, Wu FY, Murray P, Ambinder RF, et al Spindle cell conversion by Kaposi’s sarcoma-associated herpesvirus: formation of colonies and plaques with mixed lytic and latent gene expression in infected primary dermal microvascular endothelial cell cultures. J Virol. 2001;75(12):5614–26. Epub 2001/05/18. 10.1128/JVI.75.12.5614-5626.2001 11356969 PMC114274

[10] Cannon JS, Ciufo D, Hawkins AL, Griffin CA, Borowitz MJ, Hayward GS, et al A new primary effusion lymphoma-derived cell line yields a highly infectious Kaposi’s sarcoma herpesvirus-containing supernatant [In Process Citation]. J Virol 2000;74(21):10187–93. 11024147 10.1128/jvi.74.21.10187-10193.2000 PMC102057

[11] Lagunoff M, Bechtel J, Venetsanakos E, Roy AM, Abbey N, Herndier B, et al De novo infection and serial transmission of Kaposi’s sarcoma-associated herpesvirus in cultured endothelial cells. J Virol. 2002;76(5):2440–8. 11836422 10.1128/jvi.76.5.2440-2448.2002 PMC153827

[12] Renne R, Zhong W, Herndier B, McGrath M, Abbey N, Kedes D, et al Lytic growth of Kaposi’s sarcoma-associated herpesvirus (human herpesvirus 8) in culture. Nature medicine. 1996;2(3):342–6. 8612236 10.1038/nm0396-342

[13] Zhong W, Wang H, Herndier B, Ganem D. Restricted expression of Kaposi sarcoma-associated herpesvirus (human herpesvirus 8) genes in Kaposi sarcoma. Proc Natl Acad Sci U S A. 1996;93(13):6641–6. 8692871 10.1073/pnas.93.13.6641 PMC39079

[14] Staskus KA, Zhong W, Gebhard K, Herndier B, Wang H, Renne R, et al Kaposi’s sarcoma-associated herpesvirus gene expression in endothelial (spindle) tumor cells. J Virol. 1997;71(1):715–9. 8985403 10.1128/jvi.71.1.715-719.1997 PMC191104

[15] Kellam P, Bourboulia D, Dupin N, Shotton C, Fisher C, Talbot S, et al Characterization of monoclonal antibodies raised against the latent nuclear antigen of human herpesvirus 8. J Virol. 1999;73(6):5149–55. 10233979 10.1128/jvi.73.6.5149-5155.1999 PMC112561

[16] Simpson GR, Schulz TF, Whitby D, Cook PM, Boshoff C, Rainbow L, et al Prevalence of Kaposi’s sarcoma associated herpesvirus infection measured by antibodies to recombinant capsid protein and latent immunofluorescence antigen [see comments]. Lancet. 1996;348(9035):1133–8. 10.1016/S0140-6736(96)07560-5 8888167

[17] Parravicini C, Chandran B, Corbellino M, Berti E, Paulli M, Moore PS, et al Differential viral protein expression in Kaposi’s sarcoma-associated herpesvirus-infected diseases: Kaposi’s sarcoma, primary effusion lymphoma, and multicentric Castleman’s disease. Am. J Pathol 2000 3;156(3):743–9. 10.1016/S0002-9440(10)64940-1 10702388 PMC1876837

[18] Katano H, Sato Y, Kurata T, Mori S, Sata T. Expression and localization of human herpesvirus 8-encoded proteins in primary effusion lymphoma, Kaposi’s sarcoma, and multicentric Castleman’s disease. Virology. 2000 4 10;269(2):335–44. 10.1006/viro.2000.0196 10753712

[19] Katano H, Sato Y, Itoh H, Sata T. Expression of human herpesvirus 8 (HHV-8)-encoded immediate early protein, open reading frame 50, in HHV-8-associated diseases. Journal of human virology. 2001;4(2):96–102. . 11437319

[20] Brousset P, Cesarman E, Meggetto F, Lamant L, Delsol G. Colocalization of the viral interleukin-6 with latent nuclear antigen-1 of human herpesvirus-8 in endothelial spindle cells of Kaposi’s sarcoma and lymphoid cells of multicentric Castleman’s disease. Human pathology. 2001;32(1):95–100. 10.1053/hupa.2001.21131 . 11172301

[21] Chiou CJ, Poole LJ, Kim PS, Ciufo DM, Cannon JS, ap Rhys CM, et al Patterns of gene expression and a transactivation function exhibited by the vGCR (ORF74) chemokine receptor protein of Kaposi’s sarcoma-associated herpesvirus. J Virol. 2002;76(7):3421–39. Epub 2002/03/09. 10.1128/JVI.76.7.3421-3439.2002 11884567 PMC136009

[22] Abe Y, Matsubara D, Gatanaga H, Oka S, Kimura S, Sasao Y, et al Distinct expression of Kaposi’s sarcoma-associated herpesvirus-encoded proteins in Kaposi’s sarcoma and multicentric Castleman’s disease. Pathol Int. 2006;56(10):617–24. 10.1111/j.1440-1827.2006.02017.x . 16984619

[23] Dittmer DP. Transcription profile of Kaposi’s sarcoma-associated herpesvirus in primary Kaposi’s sarcoma lesions as determined by real-time PCR arrays. Cancer research. 2003;63(9):2010–5. . 12727810

[24] Hosseinipour MC, Sweet KM, Xiong J, Namarika D, Mwafongo A, Nyirenda M, et al Viral profiling identifies multiple subtypes of Kaposi’s sarcoma. mBio. 2014;5(5):e01633–14. 10.1128/mBio.01633-14 25249280 PMC4173763

[25] Bruce AG, Barcy S, DiMaio T, Gan E, Garrigues HJ, Lagunoff M, et al Quantitative Analysis of the KSHV Transcriptome Following Primary Infection of Blood and Lymphatic Endothelial Cells. Pathogens. 2017;6(1). 10.3390/pathogens6010011 28335496 PMC5371899

[26] Tso FY, Kossenkov AV, Lidenge SJ, Ngalamika O, Ngowi JR, Mwaiselage J, et al RNA-Seq of Kaposi’s sarcoma reveals alterations in glucose and lipid metabolism. PLoS pathogens. 2018;14(1):e1006844 10.1371/journal.ppat.1006844 29352292 PMC5792027

[27] Krown SE, Metroka C, Wernz JC. Kaposi’s sarcoma in the acquired immune deficiency syndrome: a proposal for uniform evaluation, response, and staging criteria. AIDS Clinical Trials Group Oncology Committee. Journal of clinical oncology: official journal of the American Society of Clinical Oncology. 1989;7(9):1201–7. 10.1200/JCO.1989.7.9.1201 . 2671281

[28] Bruce AG, Bakke AM, Bielefeldt-Ohmann H, Ryan JT, Thouless ME, Tsai CC, et al High levels of retroperitoneal fibromatosis (RF)-associated herpesvirus in RF lesions in macaques are associated with ORF73 LANA expression in spindleoid tumour cells. The Journal of general virology. 2006;87(Pt 12):3529–38. 10.1099/vir.0.82339-0 . 17098967

[29] Kliche S, Nagel W, Kremmer E, Atzler C, Ege A, Knorr T, et al Signaling by human herpesvirus 8 kaposin A through direct membrane recruitment of cytohesin-1. Molecular cell. 2001;7(4):833–43. . 11336706 10.1016/s1097-2765(01)00227-1

[30] Muralidhar S, Pumfery AM, Hassani M, Sadaie MR, Kishishita M, Brady JN, et al Identification of kaposin (open reading frame K12) as a human herpesvirus 8 (Kaposi’s sarcoma-associated herpesvirus) transforming gene [published erratum appears in J Virol 1999 Mar;73(3):2568]. J Virol. 1998;72(6):4980–8. 9573267 10.1128/jvi.72.6.4980-4988.1998 PMC110060

[31] Ye F, Lei X, Gao SJ. Mechanisms of Kaposi’s Sarcoma-Associated Herpesvirus Latency and Reactivation. Advances in virology. 2011;2011 10.1155/2011/193860 21625290 PMC3103228

[32] Full F, Jungnickl D, Reuter N, Bogner E, Brulois K, Scholz B, et al Kaposi’s sarcoma associated herpesvirus tegument protein ORF75 is essential for viral lytic replication and plays a critical role in the antagonization of ND10-instituted intrinsic immunity. PLoS pathogens. 2014;10(1):e1003863 10.1371/journal.ppat.1003863 24453968 PMC3894210

[33] Pietrek M, Brinkmann MM, Glowacka I, Enlund A, Havemeier A, Dittrich-Breiholz O, et al Role of the Kaposi’s sarcoma-associated herpesvirus K15 SH3 binding site in inflammatory signaling and B-cell activation. J Virol. 2010;84(16):8231–40. 10.1128/JVI.01696-09 20534855 PMC2916533

[34] Bala K, Bosco R, Gramolelli S, Haas DA, Kati S, Pietrek M, et al Kaposi’s sarcoma herpesvirus K15 protein contributes to virus-induced angiogenesis by recruiting PLCgamma1 and activating NFAT1-dependent RCAN1 expression. PLoS pathogens. 2012;8(9):e1002927 Epub 2012/10/03. 10.1371/journal.ppat.1002927 23028325 PMC3460623

[35] Gramolelli S, Weidner-Glunde M, Abere B, Viejo-Borbolla A, Bala K, Ruckert J, et al Inhibiting the Recruitment of PLCgamma1 to Kaposi’s Sarcoma Herpesvirus K15 Protein Reduces the Invasiveness and Angiogenesis of Infected Endothelial Cells. PLoS pathogens. 2015;11(8):e1005105 Epub 2015/08/22. 10.1371/journal.ppat.1005105 26295810 PMC4546648

[36] Poole LJ, Zong JC, Ciufo DM, Alcendor DJ, Cannon JS, Ambinder R, et al Comparison of genetic variability at multiple loci across the genomes of the major subtypes of Kaposi’s sarcoma-associated herpesvirus reveals evidence for recombination and for two distinct types of open reading frame K15 alleles at the right-hand end. J Virol. 1999;73(8):6646–60. 10400762 10.1128/jvi.73.8.6646-6660.1999 PMC112749

[37] Kakoola DN, Sheldon J, Byabazaire N, Bowden RJ, Katongole-Mbidde E, Schulz TF, et al Recombination in human herpesvirus-8 strains from Uganda and evolution of the K15 gene. The Journal of general virology. 2001;82(Pt 10):2393–404. 10.1099/0022-1317-82-10-2393 . 11562533

[38] Meng YX, Spira TJ, Bhat GJ, Birch CJ, Druce JD, Edlin BR, et al Individuals from North America, Australasia, and Africa are infected with four different genotypes of human herpesvirus 8. Virology. 1999;261(1):106–19. 10.1006/viro.1999.9853 10441559

[39] Olp LN, Jeanniard A, Marimo C, West JT, Wood C. Whole-Genome Sequencing of Kaposi’s Sarcoma-Associated Herpesvirus from Zambian Kaposi’s Sarcoma Biopsy Specimens Reveals Unique Viral Diversity. J Virol. 2015;89(24):12299–308. 10.1128/JVI.01712-15 26423952 PMC4665246

[40] Hayward GS, Zong JC. Modern evolutionary history of the human KSHV genome. Curr Top Microbiol Immunol. 2007;312:1–42. Epub 2006/11/09. . 17089792 10.1007/978-3-540-34344-8_1

[41] Li H, Komatsu T, Dezube BJ, Kaye KM. The Kaposi’s sarcoma-associated herpesvirus K12 transcript from a primary effusion lymphoma contains complex repeat elements, is spliced, and initiates from a novel promoter. J Virol. 2002;76(23):11880–8. 10.1128/JVI.76.23.11880-11888.2002 12414930 PMC136876

[42] Sarid R, Wiezorek JS, Moore PS, Chang Y. Characterization and cell cycle regulation of the major Kaposi’s sarcoma-associated herpesvirus (human herpesvirus 8) latent genes and their promoter. J Virol. 1999;73(2):1438–46. 9882349 10.1128/jvi.73.2.1438-1446.1999 PMC103968

[43] Grundhoff A, Ganem D. Mechanisms governing expression of the v-FLIP gene of Kaposi’s sarcoma-associated herpesvirus. J Virol. 2001;75(4):1857–63. 10.1128/JVI.75.4.1857-1863.2001 11160684 PMC114095

[44] Sadler R, Wu L, Forghani B, Renne R, Zhong W, Herndier B, et al A complex translational program generates multiple novel proteins from the latently expressed kaposin (K12) locus of Kaposi’s sarcoma-associated herpesvirus. J Virol. 1999;73(7):5722–30. 10364323 10.1128/jvi.73.7.5722-5730.1999 PMC112632

[45] McCormick C, Ganem D. The kaposin B protein of KSHV activates the p38/MK2 pathway and stabilizes cytokine mRNAs. Science. 2005;307(5710):739–41. 10.1126/science.1105779 . 15692053

[46] Forte E, Raja AN, Shamulailatpam P, Manzano M, Schipma MJ, Casey JL, et al MicroRNA-mediated transformation by the Kaposi’s sarcoma-associated herpesvirus Kaposin locus. J Virol. 2015;89(4):2333–41. 10.1128/JVI.03317-14 25505059 PMC4338870

[47] Morris DR, Geballe AP. Upstream open reading frames as regulators of mRNA translation. Molecular and cellular biology. 2000;20(23):8635–42. 11073965 10.1128/mcb.20.23.8635-8642.2000 PMC86464

[48] Gottwein E. Kaposi’s Sarcoma-Associated Herpesvirus microRNAs. Frontiers in microbiology. 2012;3:165 10.3389/fmicb.2012.00165 22563327 PMC3342587

[49] Pearce M, Matsumura S, Wilson AC. Transcripts encoding K12, v-FLIP, v-cyclin, and the microRNA cluster of Kaposi’s sarcoma-associated herpesvirus originate from a common promoter. J Virol. 2005;79(22):14457–64. 10.1128/JVI.79.22.14457-14464.2005 16254382 PMC1280212

[50] Cai X, Cullen BR. Transcriptional origin of Kaposi’s sarcoma-associated herpesvirus microRNAs. J Virol. 2006;80(5):2234–42. 10.1128/JVI.80.5.2234-2242.2006 16474131 PMC1395403

[51] Gandy SZ, Linnstaedt SD, Muralidhar S, Cashman KA, Rosenthal LJ, Casey JL. RNA editing of the human herpesvirus 8 kaposin transcript eliminates its transforming activity and is induced during lytic replication. J Virol. 2007;81(24):13544–51. 10.1128/JVI.01521-07 17913828 PMC2168827

[52] Majerciak V, Ni T, Yang W, Meng B, Zhu J, Zheng ZM. A viral genome landscape of RNA polyadenylation from KSHV latent to lytic infection. PLoS pathogens. 2013;9(11):e1003749 10.1371/journal.ppat.1003749 24244170 PMC3828183

[53] Weinstein JN, Myers T, Buolamwini J, Raghavan K, van Osdol W, Licht J, et al Predictive statistics and artificial intelligence in the U.S. National Cancer Institute’s Drug Discovery Program for Cancer and AIDS. Stem cells. 1994;12(1):13–22. 10.1002/stem.5530120106 . 8142917

[54] Grayson W, Pantanowitz L. Histological variants of cutaneous Kaposi sarcoma. Diagnostic pathology. 2008;3:31 10.1186/1746-1596-3-31 18655700 PMC2526984

[55] Wang Y, Li H, Chan MY, Zhu FX, Lukac DM, Yuan Y. Kaposi’s sarcoma-associated herpesvirus ori-Lyt-dependent DNA replication: cis-acting requirements for replication and ori-Lyt-associated RNA transcription. J Virol. 2004;78(16):8615–29. 10.1128/JVI.78.16.8615-8629.2004 15280471 PMC479094

[56] McClure LV, Kincaid RP, Burke JM, Grundhoff A, Sullivan CS. Comprehensive mapping and analysis of Kaposi’s sarcoma-associated herpesvirus 3' UTRs identify differential posttranscriptional control of gene expression in lytic versus latent infection. J Virol. 2013;87(23):12838–49. 10.1128/JVI.02374-13 24067953 PMC3838127

[57] Sun R, Lin SF, Staskus K, Gradoville L, Grogan E, Haase A, et al Kinetics of Kaposi’s sarcoma-associated herpesvirus gene expression. J Virol. 1999;73(3):2232–42. 9971806 10.1128/jvi.73.3.2232-2242.1999 PMC104468

[58] Sturzl M, Blasig C, Schreier A, Neipel F, Hohenadl C, Cornali E, et al Expression of HHV-8 latency-associated T0.7 RNA in spindle cells and endothelial cells of AIDS-associated, classical and African Kaposi’s sarcoma. International journal of cancer. 1997;72(1):68–71. 9212225 10.1002/(sici)1097-0215(19970703)72:1<68::aid-ijc10>3.0.co;2-6

[59] Sturzl M, Wunderlich A, Ascherl G, Hohenadl C, Monini P, Zietz C, et al Human herpesvirus-8 (HHV-8) gene expression in Kaposi’s sarcoma (KS) primary lesions: an in situ hybridization study. Leukemia. 1999;13 Suppl 1:S110–2. 10232382 10.1038/sj.leu.2401323

[60] Reed JA, Nador RG, Spaulding D, Tani Y, Cesarman E, Knowles DM. Demonstration of Kaposi’s sarcoma-associated herpes virus cyclin D homolog in cutaneous Kaposi’s sarcoma by colorimetric in situ hybridization using a catalyzed signal amplification system. Blood. 1998;91(10):3825–32. 9573020

[61] Sturzl M, Hohenadl C, Zietz C, Castanos-Velez E, Wunderlich A, Ascherl G, et al Expression of K13/v-FLIP gene of human herpesvirus 8 and apoptosis in Kaposi’s sarcoma spindle cells. J Natl Cancer Inst. 1999;91(20):1725–33. 10528022 10.1093/jnci/91.20.1725

[62] Bieleski L, Talbot SJ. Kaposi’s sarcoma-associated herpesvirus vCyclin open reading frame contains an internal ribosome entry site. J Virol. 2001;75(4):1864–9. 10.1128/JVI.75.4.1864-1869.2001 11160685 PMC114096

[63] Abere B, Mamo TM, Hartmann S, Samarina N, Hage E, Ruckert J, et al The Kaposi’s sarcoma-associated herpesvirus (KSHV) non-structural membrane protein K15 is required for viral lytic replication and may represent a therapeutic target. PLoS pathogens. 2017;13(9):e1006639 10.1371/journal.ppat.1006639 28938025 PMC5627962

[64] Fakhari FD, Dittmer DP. Charting latency transcripts in Kaposi’s sarcoma-associated herpesvirus by whole-genome real-time quantitative PCR. J Virol. 2002;76(12):6213–23. 10.1128/JVI.76.12.6213-6223.2002 12021355 PMC136228

[65] Langmead B, Salzberg SL. Fast gapped-read alignment with Bowtie 2. Nature methods. 2012;9(4):357–9. 10.1038/nmeth.1923 22388286 PMC3322381

[66] Kim D, Pertea G, Trapnell C, Pimentel H, Kelley R, Salzberg SL. TopHat2: accurate alignment of transcriptomes in the presence of insertions, deletions and gene fusions. Genome biology. 2013;14(4):R36 10.1186/gb-2013-14-4-r36 23618408 PMC4053844

[67] Afgan E, Baker D, van den Beek M, Blankenberg D, Bouvier D, Cech M, et al The Galaxy platform for accessible, reproducible and collaborative biomedical analyses: 2016 update. Nucleic acids research. 2016;44(W1):W3–W10. 10.1093/nar/gkw343 . 27137889 PMC4987906

[68] Robinson JT, Thorvaldsdottir H, Winckler W, Guttman M, Lander ES, Getz G, et al Integrative genomics viewer. Nature biotechnology. 2011;29(1):24–6. 10.1038/nbt.1754 21221095 PMC3346182

[69] Anders S, Pyl PT, Huber W. HTSeq—a Python framework to work with high-throughput sequencing data. Bioinformatics. 2015;31(2):166–9. Epub 2014/09/28. 10.1093/bioinformatics/btu638 25260700 PMC4287950

