# Supplementary material for: Quantitative RNAseq analysis of Ugandan KS tumors reveals KSHV gene expression dominated by transcription from the LTd downstream latency promoter
Source: PLoS Pathog. 2018 Dec 17;14(12):e1007441. doi: 10.1371/journal.ppat.1007441 (PMC6312348; doi:10.1371/journal.ppat.1007441)
Supplement: S1 Fig — (PDF) [file ppat.1007441.s001.pdf]

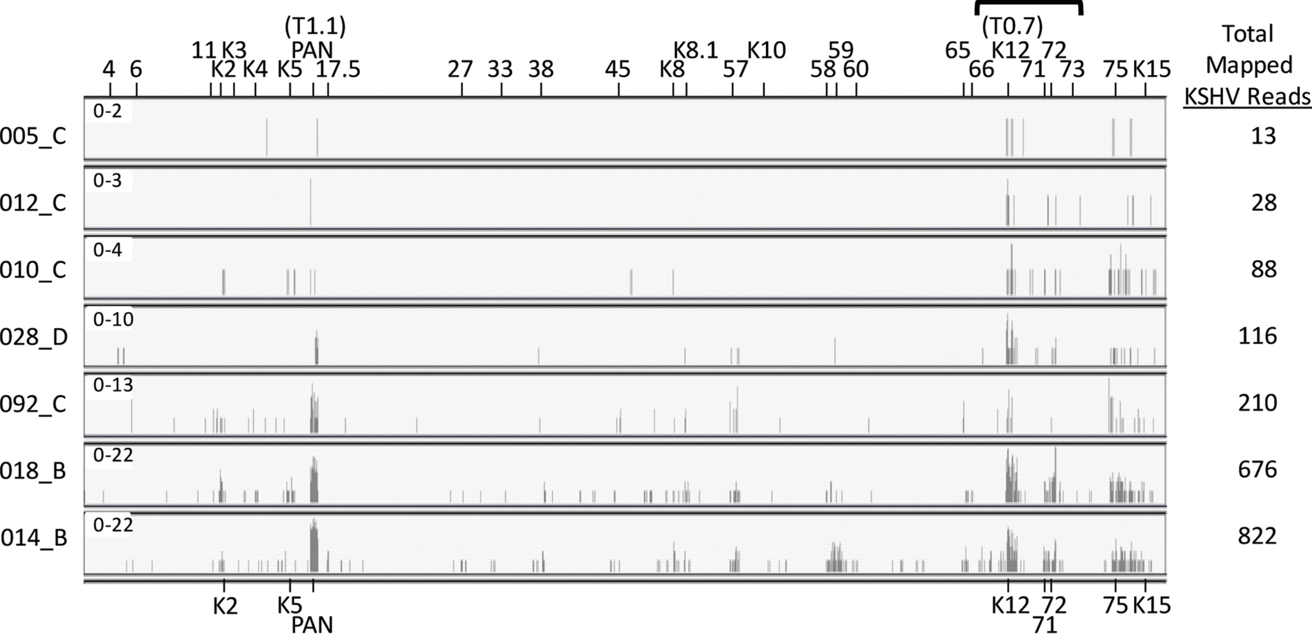

S1 Fig. KSHV transcriptome analysis of Ugandan KS tumor biopsies with <1,000 total KSHV mapped reads. RNA reads from KS tumors containing less than 1,000 total KSHV-mapped reads were aligned to the complete reference genomic sequence of KSHV strain GK18 (NC_009333) and visualized using IGV from the left to the right end of the genome. The vertical axis represents the number of reads aligned to each nucleotide position of the KSHV reference genome (autoscaled) and the visible axis range is shown on the left (log scale). Data from 7 KS tumors are shown in ascending order based on total KSHV mapped read levels, ranging from 13 (005_C) to 822 (014_B). The total number of reads mapping to both human and KSHV genomes in these samples ranged from 3.5×10^7 (092_C) to 1.2×10^8 (018_B) (S1 Table). The positions of various KSHV genes across the KSHV genome are indicated at the top and highly expressed genes are indicated at the bottom.
