# Supplementary material for: Quantitative RNAseq analysis of Ugandan KS tumors reveals KSHV gene expression dominated by transcription from the LTd downstream latency promoter
Source: PLoS Pathog. 2018 Dec 17;14(12):e1007441. doi: 10.1371/journal.ppat.1007441 (PMC6312348; doi:10.1371/journal.ppat.1007441)
Supplement: S2 Fig — (PDF) [file ppat.1007441.s002.pdf]

S2 Figure. Characterization of latency transcripts by RT-PCR

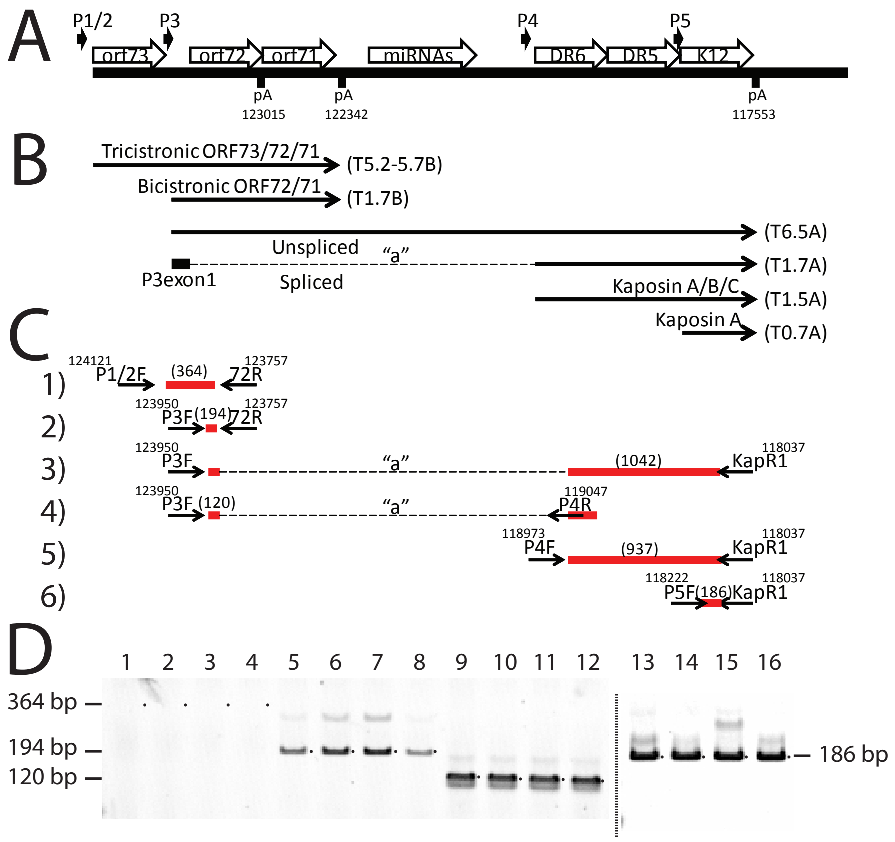

S2 Figure. Characterization of latency transcripts by RT-PCR. A) map of the latency region showing positions of ORFs, promoters (P) and transcription termination sites (pA), positions based on the NC_009333 sequence. B) Major spliced and unspliced transcripts in the latency region, as indicated in Figure 5. C) PCR assays to identify transcripts, showing primers (arrows) and position within NC_009333 sequence, PCR products (red line) and size, and splicing (dashed line). The PCR assays C1, C2, C5 and C6 were initially tested on BCBL-1 DNA. PCR fragments of the expected size were obtained in assays C1, C2 and C6. Due to the length of the PCR product spanning the DR5 and DR6 repetitive regions and the high GC content, the C5 PCR assay of BCBL-1 DNA was negative. The C3 and C4 PCR assays were not tested on BCBL-1 DNA due to the large size of the DNA fragment containing the intron region. D) Results of RT-PCR analysis. RNA from KS tumors 001_C (lanes 1, 5, 9, 13), 006_B (lanes 2, 6, 10, 14), 023_B (lanes 3 7, 11, 15), 029_B (lanes 4, 8, 12, 16) were analyzed by RT-PCR, as indicated in Materials and Methods. Assay C1 (P1/2F:72R) – This RT-PCR assay targets the junction region between ORFs 73 and 72 within the tricistronic ORF73/72/71 transcripts T5.2B, T5.4B, T5.5B, T5.7B derived from promoters P1(LTc) or P2(LTi), yielding a 406 bp fragment (see Fig 5). None of the tricistronic transcripts were detected using this RT-PCR assay in any of the KS tumors tested (lanes 1-4), confirming the lack of RNAseq reads mapping to the ORF73 UCDS feature. Assay C2 (P3F:72R) – This RT-PCR assay targets the region at the 5' end of ORF72 that is present in all of the tricistronic ORF73/72/71 transcripts, the bicistronic ORF72/71 T1.7B transcript and the monocistronic ORF72 T1.0C transcript, yielding a 194 bp fragment. Low to moderate levels of the 194 bp product were detected in all of the tumors (lanes 5-8), indicating the presence of either the bicistronic ORF72/71 T1.7B transcript or the unspliced T6.5A transcript, both from the P3(LTd) promoter. Since minimal numbers of RNAseq reads mapped to the miRNA UCDS feature targeting the T6.5A transcript, the RT-PCR data confirms the moderate levels of the bicistronic ORF72/71 T1.7B transcript detected by RNAseq. Assay C4 (P3F:P4R) – This RT-PCR assay targets the spliced T1.7A transcript with a forward primer in P3exon1 (P3F) and a reverse primer (P4R), which spans the excised intron yielding a 120 bp fragment. The entire DR5 and DR6 region is repetitive with no possible unique primer sequences. High levels of the 120 bp fragment were detected in all the tumor samples (lanes 9-12), confirming the high levels of RNAseq reads mapping to the K12Aa and DR6 UCDS features and the high levels of reads split across intron "a" (Fig 5 and Table 2). Assay C6 (P5F:KapR1) – This RT-PCR assay targets the K12 sequence, which is present in the unspliced T6.5A, T1.5A and T0.7A transcripts, as well as the spliced T1.7A transcript, yielding a 186 bp fragment. High levels of the 186 bp product were detected in all of the tumor samples (lanes 13-16), confirming the high levels of RNAseq reads mapping to the K12A UCDS feature (Fig 5). Dots adjacent to each lane show the expected position of the RT-PCR fragment. The RT-PCR assays C4 (P3F:KapR1) and C5 (P4F:KapR1) were not used to test the tumor RNAs due to the large product size spanning the DR5 and DR6 repeat region and the inability to efficiently PCR across this region of high GC content.
