# Supplementary material for: Quantitative RNAseq analysis of Ugandan KS tumors reveals KSHV gene expression dominated by transcription from the LTd downstream latency promoter
Source: PLoS Pathog. 2018 Dec 17;14(12):e1007441. doi: 10.1371/journal.ppat.1007441 (PMC6312348; doi:10.1371/journal.ppat.1007441)
Supplement: S3 Fig — (PDF) [file ppat.1007441.s003.pdf]

S3 Figure: Comparison of gene expression in bicistronic loci

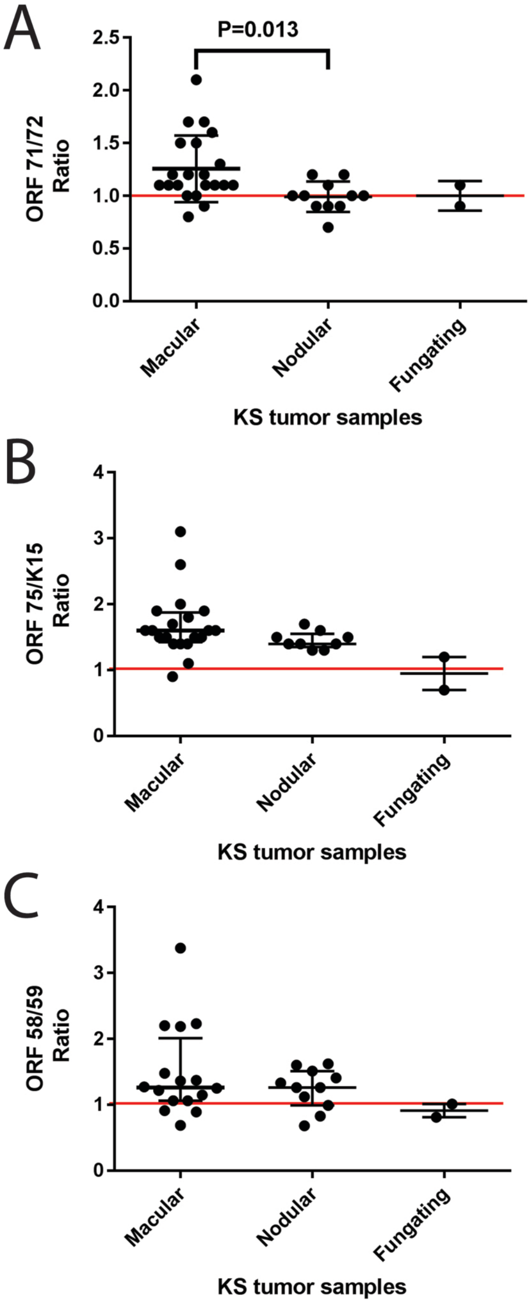

S3 Figure: Comparison of gene expression in bicistronic loci. The transcript levels detected by UCDS features targeting ORFs in the A) 5’ ORF72/ORF71 3’, B) 5’ ORF-K15/ORF75 3’, and C) 5’ ORF59/ORF58 3’ bicistronic loci were compared across 34 KS tumors and plotted according to the associated tumor morphotype. The ratio was determined between the transcripts mapping to the 3’ downstream ORF (PolyA flanking) and those mapping to the 5’ upstream ORF (5’ CAP flanking) in loci with known bicistronic transcripts. A ratio of 1, indicative of a bicistronic transcript, is indicated in red. The median and intraquartile range is shown. Ratios above 1 indicate the presence of a monocistronic transcript encoding the 3’ downstream PolyA-flanking ORF: A) ORF71 monocistronic transcript T0.9B (see Fig 5B; S2 Table): P3(LTd) promoter, TSS = bp 124,009, splice donor = bp 123,843, splice acceptor = bp 123,107, polyA termination signal = bp 122,342; B) ORF58 monocistronic transcript T1.3: TSS = bp 95,930, polyA termination signal = bp 94,594 [1]; C) ORF75 monocistronic transcript T4.2: TSS bp 134,891 [2], polyA termination signal = bp 130,660 [1]. Ratios below 1 indicate the presence of a monocistronic transcript encoding the 5’ upstream ORF which terminates prior to the position of the downstream ORF: A) ORF72 monocistronic transcript T1.0C (see Fig 5B; S2 Table): P3(LTd) promoter, TSS = bp 124,009/124,090, polyA termination signal = bp 123,015; B) ORF59 monocistronic transcript = unknown; C) K15 monocistronic transcript = unknown.

References

1. Majerciak V, Yamanegi K, Zheng ZM. Gene structure and expression of Kaposi's sarcoma-associated herpesvirus ORF56, ORF57, ORF58, and ORF59. J Virol. 2006;80(24):11968-81. doi: 10.1128/JVI.01394-06. PubMed PMID: 17020939; PubMed Central PMCID: PMC1676266.
2. Bruce AG, Barcy S, DiMaio T, Gan E, Garrigues HJ, Lagunoff M, et al. Quantitative Analysis of the KSHV Transcriptome Following Primary Infection of Blood and Lymphatic Endothelial Cells. Pathogens. 2017;6(1). doi: 10.3390/pathogens6010011. PubMed PMID: 28335496; PubMed Central PMCID: PMC5371899.
