# Supplementary material for: Quantitative RNAseq analysis of Ugandan KS tumors reveals KSHV gene expression dominated by transcription from the LTd downstream latency promoter
Source: PLoS Pathog. 2018 Dec 17;14(12):e1007441. doi: 10.1371/journal.ppat.1007441 (PMC6312348; doi:10.1371/journal.ppat.1007441)
Supplement: S4 Fig — (PDF) [file ppat.1007441.s004.pdf]

S4 Fig. Kaposin T1.7A transcripts and encoding potential. The nucleotide sequences of the T1.7A spliced transcript are shown for a number of KSHV strains. Upstream and downstream flanking sequences are italicized and the three possible reading frames are in upper case. Open reading frames are colored and annotated and the position of promoters, transcript start stites (TSS) and poly-adenylation signals are shown. The nucleotides flanking the splice position are shown and possible CUG initiation codons are highlighted in red.

GK18 Kaposin T1.7A transcript and flanking sequences (NC_009333: bp 124,091-117,977)

```
                                     ORF73|     P3 promoter??>
      K  V  L  G  T  L  H  R  K  *  H  K  S  H  T  S  P  P  F  S     F1
       R  S  W  G  L  S  T  G  N  D  I  K  A  T  P  L  P  L  F  P    F2
        G  P  G  D  S  P  Q  E  M  T  *  K  P  H  L  S  P  F  F  L   F3
   1  aaggtcctggggactctccacaggaaatgacataaaagccacacctctcccccttttttcc 60
      ----:----|----:----|----:----|----:----|----:----|----:----|

                   TSS T1.7A>                uORF1>
      S  L  E  A  T  V  A  A  P  H  L  H  L  A  P  W  V  L  V  C     F1
       P  *  K  P  P  S  P  L  R  T  C  I  W  R  H  G  C  W  C  V    F2
        P  R  S  H  R  R  R  S  A  L  A  F  G  A  M  G  A  G  V  C   F3
  61  tccctagaagccaccgtcgccgctccgcacttgcatttggcgccatgggtgctggtgtgt 120
      ----:----|----:----|----:----|----:----|----:----|----:----|

      V  W  G  S  V  L  T  T  H  L  P  Q  L  N  T  R  T  T  A  S     F1
       C  G  A  V  F  S  R  P  I  Y  L  N  *  T  H  G  Q  R  L  A    F2
        V  G  Q  C  S  H  D  P  S  T  S  T  E  H  T  D  N  G  *  R   F3
 121  gtgtggggcagtgttctcacgacccatctacctcaactgaacacacggacaacggctagc 180
      ----:----|----:----|----:----|----:----|----:----|----:----|

                                             Potential CUG uORF3>
                                uORF2>    Potential CUG ORF>
      V  L  S  R  P  S  V  V  D  G  R  T  *  Q  S  T  L  K  L  Q     F1
       Y  S  R  G  P  A  S  S  M  G  E  P  D  R  A  P  *  N  S  R    F2
        T  L  A  A  Q  R  R  R  W  E  N  L  T  E  H  P  E  T  P  G   F3
 181  gtactctcgcggcccagcgtcgtcgatgggagaacctgacagagcaccctgaaactccag 240
      ----:----|----:----|----:----|----:----|----:----|----:----|

       Splice site  P4 promoter>               TSS (T1.5A)>
      A  L  Q  E  T  L  *  R  R  G  E  T  D  P  P  R  A  L  R  A     F1
       L  Y  R  K  R  Y  K  E  E  G  R  P  T  P  L  A  H  Y  A  R    F2
        S  T  G  N  A  I  K  K  R  G  D  R  P  P  S  R  I  T  R  A   F3
 241  gctctacaggaaacgctataaagaagaggggagaccgaccccctcgcgcattacgcgcg 300
      ----:----|----:----|----:----|----:----|----:----|----:----|

      R  R  H  G  G  R  I  S  W  I  Y  T  Y  R  G  A  V  A  P  Q     F1
       G  G  T  E  D  G  S  L  G  F  T  R  I  E  E  R  W  H  P  R    F2
        A  A  R  R  T  D  L  L  D  L  H  V  S  R  S  G  G  T  P  G   F3
 301  cggcggcacggaggacggatctcttggatttacacgtatcgaggagcggtggcaccccag 360
      ----:----|----:----|----:----|----:----|----:----|----:----|

       Major CUG ORF (BCBL)>                 Minor CUG ORF (BCBL)>
      E  P  V  L  A  H  P  R  N  Q  V  V  P  P  N  P  A  N  P  A     F1
       N  P  S  W  H  T  P  G  T  R  *  Y  P  R  T  L  R  T  L  Q    F2
        T  R  P  G  T  P  Q  E  P  G  S  T  P  E  P  C  E  P  C  S   F3
 361  gaacccgtcctggcacaccccaggaaccaggtagtacccccgaaccctgcgaaccctgca 420
      ----:----|----:----|----:----|----:----|----:----|----:----|
```

```
    V  P  R  R  G  P  P  P  R  T  P  H  P  R  N  P  A  R  R  T     F1
     Y  P  G  A  V  P  P  P  A  P  R  T  P  G  T  R  R  G  A  P    F2
      T  P  A  R  S  P  P  P  H  P  A  P  Q  E  P  G  A  A  H  P   F3
421 gtaccccggcgcggtcccccccccgcaccccgcaccccaggaacccggcgcggcgcacc 480
    ----:----|----:----|----:----|----:----|----:----|----:----|

    P  G  T  R  R  G  A  P  Q  E  P  G  A  A  H  P  R  N  P  A     F1
     Q  E  P  G  A  A  H  P  R  N  P  A  R  R  T  P  G  T  R  R    F2
      R  N  P  A  R  R  T  P  G  T  R  R  G  A  P  Q  E  P  G  A   F3
481 ccaggaacccggcgcggcgcaccccaggaacccggcgcggcgcaccccaggaacccggcg 540
    ----:----|----:----|----:----|----:----|----:----|----:----|

    R  R  T  P  G  T  R  R  G  A  P  Q  E  P  G  A  A  H  P  R     F1
     G  A  P  Q  E  P  G  A  A  H  P  R  N  P  A  R  R  T  P  G    F2
      A  H  P  R  N  P  A  R  R  T  P  G  T  R  R  G  A  P  Q  E   F3
541 cggcgcaccccaggaacccggcgcggcgcaccccaggaacccggcgcggcgcaccccagg 600
    ----:----|----:----|----:----|----:----|----:----|----:----|

    N  P  A  R  R  T  P  G  T  R  R  G  A  P  R  E  P  G  A  A     F1
     T  R  R  G  A  P  Q  E  P  G  A  A  H  P  G  N  P  A  R  R    F2
      P  G  A  A  H  P  R  N  P  A  R  R  T  P  G  T  R  R  G  A   F3
601 aacccggcgcggcgcaccccaggaacccggcgcggcgcaccccgggaacccggcgcggcg 660
    ----:----|----:----|----:----|----:----|----:----|----:----|

    H  P  G  N  P  A  R  R  T  P  G  T  R  R  G  A  P  Q  E  P     F1
     T  P  G  T  R  R  G  A  P  R  E  P  G  A  A  H  P  R  N  P    F2
      P  R  E  P  G  A  A  H  P  G  N  P  A  R  R  T  P  G  T  R   F3
661 caccccgggaacccggcgcggcgcaccccgggaacccggcgcggcgcaccccaggaaccc 720
    ----:----|----:----|----:----|----:----|----:----|----:----|

    G  A  A  H  P  R  N  P  A  R  R  T  P  G  T  R  R  G  A  P     F1
     A  R  R  T  P  G  T  R  R  G  A  P  R  E  P  G  A  A  H  P    F2
      R  G  A  P  Q  E  P  G  A  A  H  P  G  N  P  A  R  R  T  P   F3
721 ggcgcggcgcaccccaggaacccggcgcggcgcaccccgggaacccggcgcggcgcaccc 780
    ----:----|----:----|----:----|----:----|----:----|----:----|

    R  E  P  G  A  A  H  P  S  L  L  P  G  N  L  V  P  S  S  P     F1
     G  N  P  A  R  R  T  P  A  S  S  P  G  T  W  C  P  P  P  R    F2
      G  T  R  R  G  A  P  Q  P  P  P  R  E  P  G  A  L  L  P  G   F3
781 cgggaacccggcgcggcgcaccccagcctcctccccgggaacctggtgccctcctccccg 840
    ----:----|----:----|----:----|----:----|----:----|----:----|

    G  T  W  C  P  P  P  R  E  P  G  A  L  L  P  G  N  L  V  P     F1
     E  P  G  A  L  L  P  G  N  L  V  P  S  S  P  G  T  W  C  P    F2
      N  L  V  P  S  S  P  G  T  W  C  P  P  P  R  E  P  G  A  L   F3
841 ggaacctggtgccctcctccccgggaacctggtgccctcctccccgggaacctggtgccc 900
    ----:----|----:----|----:----|----:----|----:----|----:----|

    S  S  P  G  T  W  C  P  P  P  R  E  P  G  A  L  L  P  G  N     F1
     P  P  R  E  P  G  A  L  L  P  G  N  L  V  P  S  S  P  G  T    F2
      L  P  G  N  L  V  P  S  S  P  G  T  W  C  P  P  P  R  E  P   F3
901 tcctccccgggaacctggtgccctcctccccgggaacctggtgccctcctccccgggaac 960
    ----:----|----:----|----:----|----:----|----:----|----:----|

    L  V  P  S  S  P  G  T  W  C  P  P  P  R  E  P  G  A  L  L     F1
     W  C  P  P  P  R  E  P  G  A  L  L  P  G  N  L  V  P  S  S    F2
      G  A  L  L  P  G  N  L  V  P  S  S  P  G  T  W  C  P  P  P   F3
961 ctggtgccctcctccccgggaacctggtgccctcctccccgggaacctggtgccctcctc 1020
```

```
     ----:----|----:----|----:----|----:----|----:----|----:----|

     P  G  N  L  V  P  S  S  P  G  T  W  C  P  P  P  S  S  L  Q     F1
      P  G  T  W  C  P  P  P  R  E  P  G  A  L  L  P  P  H  S  N    F2
       R  E  P  G  A  L  L  P  G  N  L  V  P  S  S  L  L  T  P  I   F3
1021 cccgggaacctggtgccctcctccccgggaacctggtgccctcctccctcctcactccaa 1080
     ----:----|----:----|----:----|----:----|----:----|----:----|

              Kaposin A>
     S  Q  C  M  D  R  G  L  T  V  F  V  A  V  H  V  P  D  V  L     F1
      P  N  A  W  I  E  A  *  R  C  L  W  Q  F  M  S  R  M  C  Y    F2
       P  M  H  G  *  R  L  N  G  V  C  G  S  S  C  P  G  C  V  T   F3
1081 tcccaatgcatggatagaggcttaacggtgtttgtggcagttcatgtcccggatgtgtta 1140
     ----:----|----:----|----:----|----:----|----:----|----:----|

     L  N  G  W  R  W  R  L  G  A  I  P  P  L  V  C  L  L  A  I     F1
      *  M  G  G  A  G  G  L  G  R  Y  H  H  S  F  V  C  W  R  L    F2
       K  W  V  A  L  E  A  W  G  D  T  T  T  R  L  S  V  G  D  *   F3
1141 ctaaatgggtggcgctggaggcttggggcgataccaccactcgtttgtctgttggcgatt 1200
     ----:----|----:----|----:----|----:----|----:----|----:----|

     S  V  V  P  P  S  G  Q  R  G  P  V  A  F  R  T  R  V  A  T     F1
      V  L  S  P  R  V  A  S  V  A  P  *  H  S  G  H  E  L  Q  R    F2
       C  C  P  P  E  W  P  A  W  P  R  S  I  Q  D  T  S  C  N  G   F3
1201 agtgttgtccccccgagtggccagcgtggccccgtagcattcaggacacgagttgcaacg 1260
     ----:----|----:----|----:----|----:----|----:----|----:----|

                                              PolyA signal
     G  A  H  *  S  *  R  A  L  P  K  S  V  S  K  I  K  Y  K  S     F1
      A  R  T  E  A  S  V  P  S  Q  R  V  S  V  K  *  N  T  K  A    F2
       R  A  L  K  L  A  C  P  P  K  E  C  Q  *  N  K  I  Q  K  H   F3
1261 ggcgcgcactgaagctagcgtgccctcccaaagagtgtcagtaaaataaaatacaaaagc 1320
     ----:----|----:----|----:----|----:----|----:----|----:----|
```

JSC-1 Kaposin T1.7A transcript and flanking sequences (GQ994935: bp 124,285-117,690)

```
                                        ORF73|    P3 promoter??>
    R  S  W  G  L  S  T  G  N  D  I  K  A  T  P  L  P  L  F  P     F1
     G  P  G  D  S  P  Q  E  M  T  *  K  P  H  L  S  P  F  F  L    F2
      V  L  G  T  L  H  R  K  *  H  K  S  H  T  S  P  P  F  S  S   F3
  1 aggtcctggggactctccacaggaaatgacataaaagccacacctctccccctttttcct 60
    ----:----|----:----|----:----|----:----|----:----|----:----|

                     TSS T1.7A>                        uORF1>
    P  *  K  P  P  S  P  L  R  T  C  I  W  R  H  G  C  W  C  V     F1
     P  R  S  H  R  R  R  S  A  L  A  F  G  A  M  G  A  G  V  C    F2
      L  E  A  T  V  A  A  P  H  L  H  L  A  P  W  V  L  V  C  V   F3
 61 ccctagaagccaccgtcgccgctccgcacttgcatttggcgccatgggtgctggtgtgtg 120
    ----:----|----:----|----:----|----:----|----:----|----:----|

    C  G  A  V  F  S  R  P  I  Y  L  N  *  T  H  G  Q  R  L  A     F1
     V  G  Q  C  S  H  D  P  S  T  S  T  E  H  T  D  N  G  *  R    F2
      W  G  S  V  L  T  T  H  L  P  Q  L  N  T  R  T  T  A  S  V   F3
121 tgtgggcagtgttctcacgacccatctacctcaactgaacacacggacaacggctagcg 180
    ----:----|----:----|----:----|----:----|----:----|----:----|

                                                    Potential CUG uORF3>
                                uORF2>    Potential CUG ORF>
    Y  S  R  G  P  A  S  S  M  G  E  P  D  R  A  P  *  N  S  R     F1
     T  L  A  A  Q  R  R  R  W  E  N  L  T  E  H  P  E  T  P  G    F2
      L  S  R  P  S  V  V  D  G  R  T  *  Q  S  T  L  K  L  Q  A   F3
181 tactctcgcggcccagcgtcgtcgatgggagaacctgacagagcaccctgaaactccagg 240
    ----:----|----:----|----:----|----:----|----:----|----:----|

     Splice site   P4 promoter>                  TSS (T1.5A)>
    L  Y  R  K  R  Y  K  E  E  G  R  P  T  P  L  A  H  Y  A  R     F1
     S  T  G  N  A  I  K  K  R  G  D  R  P  P  S  R  I  T  R  A    F2
      L  Q  E  T  L  *  R  R  G  E  T  D  P  P  R  A  L  R  A  R   F3
241 ctctacaggaaacgctataaagaagaggggagaccgaccccctcgcgcattacgcgcgc 300
    ----:----|----:----|----:----|----:----|----:----|----:----|

    G  G  T  E  D  G  S  L  G  F  T  R  I  E  E  R  W  H  P  R     F1
     A  A  R  R  T  D  L  L  D  L  H  V  S  R  S  G  G  T  P  G    F2
      R  H  G  G  R  I  S  W  I  Y  T  Y  R  G  A  V  A  P  Q  E   F3
301 ggcggcacggaggacggatctcttggatttacacgtatcgaggagcggtggcaccccagg 360
    ----:----|----:----|----:----|----:----|----:----|----:----|

     Major CUG ORF (BCBL)>                        Minor CUG ORF (BCBL)>
    N  P  S  W  H  T  P  G  T  R  *  Y  P  R  T  L  R  T  L  Q     F1
     T  R  P  G  T  P  Q  E  P  G  S  T  P  E  P  C  E  P  C  S    F2
      P  V  L  A  H  P  R  N  Q  V  V  P  P  N  P  A  N  P  A  V   F3
361 aacccgtcctggcacaccccaggaaccaggtagtacccccgaaccctgcgaaccctgcag 420
    ----:----|----:----|----:----|----:----|----:----|----:----|

    Y  P  G  A  V  P  P  R  T  P  H  P  R  N  P  A  R  R  T  P     F1
     T  P  A  R  S  P  P  A  P  R  T  P  G  T  R  R  G  A  P  Q    F2
      P  R  R  G  P  P  P  H  P  A  P  Q  E  P  G  A  A  H  P  R   F3
421 taccccggcgcggtcccccccgcaccccgcaccccaggaacccggcgcggcgcacccca 480
    ----:----|----:----|----:----|----:----|----:----|----:----|

    G  T  R  R  G  A  P  Q  E  P  G  A  A  H  P  R  N  P  A  R     F1
     E  P  G  A  A  H  P  R  N  P  A  R  R  T  P  G  T  R  R  G    F2
```

```
       N  P  A  R  R  T  P  G  T  R  R  G  A  P  Q  E  P  G  A  A   F3
 481 ggaacccggcgcggcgcaccccaggaacccggcgcggcgcaccccaggaacccggcgcgg 540
     ----:----|----:----|----:----|----:----|----:----|----:----|

     R  T  P  G  T  R  R  G  A  P  Q  E  P  G  A  A  H  P  R  N     F1
      A  P  Q  E  P  G  A  A  H  P  R  N  P  A  R  R  T  P  G  T    F2
       H  P  R  N  P  A  R  R  T  P  G  T  R  R  G  A  P  Q  E  P   F3
 541 cgcaccccaggaacccggcgcggcgcaccccaggaacccggcgcggcgcaccccaggaac 600
     ----:----|----:----|----:----|----:----|----:----|----:----|

     P  A  R  R  T  P  G  T  R  R  G  A  P  Q  E  P  G  A  A  H     F1
      R  R  G  A  P  Q  E  P  G  A  A  H  P  R  N  P  A  R  R  T    F2
       G  A  A  H  P  R  N  P  A  R  R  T  P  G  T  R  R  G  A  P   F3
 601 ccggcgcggcgcaccccaggaacccggcgcggcgcaccccaggaacccggcgcggcgcac 660
     ----:----|----:----|----:----|----:----|----:----|----:----|

     P  R  N  P  A  R  R  T  P  G  T  R  R  G  A  P  Q  E  P  G     F1
      P  G  T  R  R  G  A  P  Q  E  P  G  A  A  H  P  R  N  P  A    F2
       Q  E  P  G  A  A  H  P  R  N  P  A  R  R  T  P  G  T  R  R   F3
 661 cccaggaacccggcgcggcgcaccccaggaacccggcgcggcgcaccccaggaacccggc 720
     ----:----|----:----|----:----|----:----|----:----|----:----|

     A  A  H  P  R  N  P  A  R  R  T  P  G  T  R  R  G  A  P  Q     F1
      R  R  T  P  G  T  R  R  G  A  P  Q  E  P  G  A  A  H  P  R    F2
       G  A  P  Q  E  P  G  A  A  H  P  R  N  P  A  R  R  T  P  G   F3
 721 gcggcgcaccccaggaacccggcgcggcgcaccccaggaacccggcgcggcgcaccccag 780
     ----:----|----:----|----:----|----:----|----:----|----:----|

     E  P  G  A  A  H  P  R  N  P  A  R  R  T  P  G  T  R  R  G     F1
      N  P  A  R  R  T  P  G  T  R  R  G  A  P  Q  E  P  G  A  A    F2
       T  R  R  G  A  P  Q  E  P  G  A  A  H  P  R  N  P  A  R  R   F3
 781 gaacccggcgcggcgcaccccaggaacccggcgcggcgcaccccaggaacccggcgcggc 840
     ----:----|----:----|----:----|----:----|----:----|----:----|

     A  P  Q  E  P  G  A  A  H  P  R  N  P  A  R  R  T  P  G  T     F1
      H  P  R  N  P  A  R  R  T  P  G  T  R  R  G  A  P  Q  E  P    F2
       T  P  G  T  R  R  G  A  P  Q  E  P  G  A  A  H  P  R  N  P   F3
 841 gcaccccaggaacccggcgcggcgcaccccaggaacccggcgcggcgcaccccaggaacc 900
     ----:----|----:----|----:----|----:----|----:----|----:----|

     R  R  G  A  P  Q  E  P  G  A  A  H  P  S  L  L  P  G  N  L     F1
      G  A  A  H  P  R  N  P  A  R  R  T  P  A  S  S  P  G  T  W    F2
       A  R  R  T  P  G  T  R  R  G  A  P  Q  P  P  P  R  E  P  G   F3
 901 cggcgcggcgcaccccaggaacccggcgcggcgcaccccagcctcctccccgggaacctg 960
     ----:----|----:----|----:----|----:----|----:----|----:----|

     V  P  S  S  P  G  T  W  C  P  P  P  R  E  P  G  A  L  L  P     F1
      C  P  P  P  R  E  P  G  A  L  L  P  G  N  L  V  P  S  S  P    F2
       A  L  L  P  G  N  L  V  P  S  S  P  G  T  W  C  P  P  P  R   F3
 961 gtgccctcctccccgggaacctggtgccctcctccccgggaacctggtgccctcctcccc 1020
     ----:----|----:----|----:----|----:----|----:----|----:----|

     G  N  L  V  P  S  S  P  G  T  W  C  P  P  P  R  E  P  G  A     F1
      G  T  W  C  P  P  P  R  E  P  G  A  L  L  P  G  N  L  V  P    F2
       E  P  G  A  L  L  P  G  N  L  V  P  S  S  P  G  T  W  C  P   F3
1021 gggaacctggtgccctcctccccgggaacctggtgccctcctccccgggaacctggtgcc 1080
     ----:----|----:----|----:----|----:----|----:----|----:----|
```

```
     L  L  P  G  N  L  V  P  S  S  P  G  T  W  C  P  P  P  R  E     F1
      S  S  P  G  T  W  C  P  P  P  R  E  P  G  A  L  L  P  G  N    F2
       P  P  R  E  P  G  A  L  L  P  G  N  L  V  P  S  S  P  G  T   F3
1081 ctcctccccgggaacctggtgccctcctccccgggaacctggtgccctcctccccgggaa 1140
     ----:----|----:----|----:----|----:----|----:----|----:----|

     P  G  A  L  L  P  G  N  L  V  P  S  S  P  G  T  W  C  P  P     F1
      L  V  P  S  S  P  G  T  W  C  P  P  P  R  E  P  G  A  L  L    F2
       W  C  P  P  P  R  E  P  G  A  L  L  P  G  N  L  V  P  S  S   F3
1141 cctggtgccctcctccccgggaacctggtgccctcctccccgggaacctggtgccctcct 1200
     ----:----|----:----|----:----|----:----|----:----|----:----|

     P  R  E  P  G  A  L  L  P  G  N  L  V  P  S  S  P  G  T  W     F1
      P  G  N  L  V  P  S  S  P  G  T  W  C  P  P  P  R  E  P  G    F2
       P  G  T  W  C  P  P  P  R  E  P  G  A  L  L  P  G  N  L  V   F3
1201 ccccgggaacctggtgccctcctccccgggaacctggtgccctcctccccgggaacctgg 1260
     ----:----|----:----|----:----|----:----|----:----|----:----|

     C  P  P  P  R  E  P  G  A  L  L  P  G  N  L  V  P  S  S  P     F1
      A  L  L  P  G  N  L  V  P  S  S  P  G  T  W  C  P  P  P  R    F2
       P  S  S  P  G  T  W  C  P  P  P  R  E  P  G  A  L  L  P  G   F3
1261 tgccctcctccccgggaacctggtgccctcctccccgggaacctggtgccctcctccccg 1320
     ----:----|----:----|----:----|----:----|----:----|----:----|

     G  T  W  C  P  P  P  R  E  P  G  A  L  L  P  G  N  L  V  P     F1
      E  P  G  A  L  L  P  G  N  L  V  P  S  S  P  G  T  W  C  P    F2
       N  L  V  P  S  S  P  G  T  W  C  P  P  P  R  E  P  G  A  L   F3
1321 ggaacctggtgccctcctccccgggaacctggtgccctcctccccgggaacctggtgccc 1380
     ----:----|----:----|----:----|----:----|----:----|----:----|

     S  S  P  G  T  W  C  P  P  P  R  E  P  G  A  L  L  P  G  N     F1
      P  P  R  E  P  G  A  L  L  P  G  N  L  V  P  S  S  P  G  T    F2
       L  P  G  N  L  V  P  S  S  P  G  T  W  C  P  P  P  R  E  P   F3
1381 tcctccccgggaacctggtgccctcctccccgggaacctggtgccctcctccccgggaac 1440
     ----:----|----:----|----:----|----:----|----:----|----:----|

     L  V  P  S  S  P  G  T  W  C  P  P  P  R  E  P  G  A  L  L     F1
      W  C  P  P  P  R  E  P  G  A  L  L  P  G  N  L  V  P  S  S    F2
       G  A  L  L  P  G  N  L  V  P  S  S  P  G  T  W  C  P  P  P   F3
1441 ctggtgccctcctccccgggaacctggtgccctcctccccgggaacctggtgccctcctc 1500
     ----:----|----:----|----:----|----:----|----:----|----:----|

     P  G  N  L  V  P  S  S  P  G  T  W  C  P  P  P  S  S  L  Q     F1
      P  G  T  W  C  P  P  P  R  E  P  G  A  L  L  P  P  H  S  N    F2
       R  E  P  G  A  L  L  P  G  N  L  V  P  S  S  L  L  T  P  I   F3
1501 cccgggaacctggtgccctcctccccgggaacctggtgccctcctccctcctcactccaa 1560
     ----:----|----:----|----:----|----:----|----:----|----:----|

              Kaposin A>
     S  Q  C  M  D  R  G  L  T  V  F  V  A  V  H  V  P  D  V  L     F1
      P  N  A  W  I  E  A  *  R  C  L  W  Q  F  M  S  R  M  C  Y    F2
       P  M  H  G  *  R  L  N  G  V  C  G  S  S  C  P  G  C  V  T   F3
1561 tcccaatgcatggatagaggcttaacggtgtttgtggcagttcatgtcccggatgtgtta 1620
     ----:----|----:----|----:----|----:----|----:----|----:----|

     L  N  G  W  R  W  R  L  G  A  I  P  P  L  V  C  L  L  A  I     F1
      *  M  G  G  A  G  G  L  G  R  Y  H  H  S  F  V  C  W  R  L    F2
       K  W  V  A  L  E  A  W  G  D  T  T  T  R  L  S  V  G  D  *   F3
```

```
1621 ctaaatgggtggcgctggaggcttggggcgataccaccactcgtttgtctgttggcgatt 1680
     ----:----|----:----|----:----|----:----|----:----|----:----|

     S  V  V  P  P  S  G  Q  R  G  P  V  A  F  R  T  R  V  A  T     F1
      V  L  S  P  R  V  A  S  V  A  P  *  H  S  G  H  E  L  Q  R    F2
       C  C  P  P  E  W  P  A  W  P  R  S  I  Q  D  T  S  C  N  G   F3
1681 agtgttgtcccccccgagtggccagcgtggccccgtagcattcaggacacgagttgcaacg 1740
     ----:----|----:----|----:----|----:----|----:----|----:----|

                                             PolyA signal
     G  A  H  *  S  *  R  A  L  P  K  S  V  S  K  I  K  Y  K  S     F1
      A  R  T  E  A  S  V  P  S  Q  R  V  S  V  K  *  N  T  K  A    F2
       R  A  L  K  L  A  C  P  P  K  E  C  Q  *  N  K  I  Q  K  H   F3
1741 ggcgcgcactgaagctagcgtgccctcccaaagagtgtcagtaaaataaaatacaaaagc 1800
     ----:----|----:----|----:----|----:----|----:----|----:----|
```

ZM114 KKaposin T1.7A spliced RNA transcript and flanking sequences (KT271460; bp 123,560-bp 117,457)

```
                                          ORF73|   P3 promoter??>
     R  S  W  G  L  S  T  G  N  D  I  K  A  T  P  L  P  L  F  P    F1
      G  P  G  D  S  P  Q  E  M  T  *  K  P  H  L  S  P  F  F  L   F2
       V  L  G  T  L  H  R  K  *  H  K  S  H  T  S  P  P  F  S  S  F3
  1 aggtcctggggactctccacaggaaatgacataaaagccacacctctccccctttttcct 60
    ----:----|----:----|----:----|----:----|----:----|----:----|

                      TSS T1.7A>                    uORF1>
     P  *  K  P  P  S  P  L  R  T  C  I  W  R  H  G  C  W  C  V    F1
      P  R  S  H  R  R  R  S  A  L  A  F  G  A  M  G  A  G  V  C   F2
       L  E  A  T  V  A  A  P  H  L  H  L  A  P  W  V  L  V  C  V  F3
 61 ccctagaagccaccgtcgccgctccgcacttgcatttggcgccatgggtgctggtgtgtg 120
    ----:----|----:----|----:----|----:----|----:----|----:----|

     C  G  A  V  F  S  R  P  I  Y  L  N  *  T  H  G  Q  R  L  A    F1
      V  G  Q  C  S  H  D  P  S  T  S  T  E  H  T  D  N  G  *  R   F2
       W  G  S  V  L  T  T  H  L  P  Q  L  N  T  R  T  T  A  S  V  F3
121 tgtggggcagtgttctcacgacccatctacctcaactgaacacacggacaacggctagcg 180
    ----:----|----:----|----:----|----:----|----:----|----:----|

                                                 Potential CUG uORF3>
                             uORF2>    Potential CUG ORF>
     Y  S  R  G  P  A  S  S  M  G  E  P  D  R  A  P  *  N  S  R    F1
      T  L  A  A  Q  R  R  R  W  E  N  L  T  E  H  P  E  T  P  G   F2
       L  S  R  P  S  V  V  D  G  R  T  *  Q  S  T  L  K  L  Q  A  F3
181 tactctcgcggcccagcgtcgtcgatgggagaacctgacagagcaccctgaaactccagg 240
    ----:----|----:----|----:----|----:----|----:----|----:----|

      Splice site  P4 promoter>              TSS (T1.5A)>
     L  Y  R  K  R  Y  K  E  E  G  R  P  T  P  S  R  I  T  R  A    F1
      S  T  G  N  A  I  K  K  R  G  D  R  P  P  R  A  L  R  A  R   F2
       L  Q  E  T  L  *  R  R  G  E  T  D  P  L  A  H  Y  A  R  G  F3
241 ctctacaggaaacgctataaagaagaggggagaccgaccccctcgcgcattacgcgcgcg 300
    ----:----|----:----|----:----|----:----|----:----|----:----|

     A  A  R  R  T  D  L  L  D  L  H  V  S  R  S  G  G  T  P  G    F1
      R  H  G  G  R  I  S  W  I  Y  T  Y  R  G  A  V  A  P  Q  E   F2
       G  T  E  D  G  S  L  G  F  T  R  I  E  E  R  W  H  P  R  N  F3
301 gcggcacggaggacggatctcttggatttacacgtatcgaggagcggtggcaccccagga 360
    ----:----|----:----|----:----|----:----|----:----|----:----|

      Major CUG ORF (BCBL)>                     Minor CUG ORF (BCBL)>
     T  R  P  G  T  P  Q  E  P  G  S  T  P  E  P  C  E  P  C  S    F1
      P  G  L  A  H  P  R  N  P  V  V  P  P  N  P  A  N  P  A  V   F2
       P  A  W  H  T  P  G  T  R  *  Y  P  R  T  L  R  T  L  Q  Y  F3
361 acccggcctggcacaccccaggaacccggtagtacccccgaaccctgcgaaccctgcagt 420
    ----:----|----:----|----:----|----:----|----:----|----:----|

     T  P  A  R  S  R  T  P  H  P  P  P  Q  E  P  G  A  A  H  P    F1
      P  R  R  G  P  A  P  R  T  P  H  P  R  N  P  A  R  R  T  P   F2
       P  G  A  V  P  H  P  A  P  P  T  P  G  T  R  R  G  A  P  Q  F3
421 accccggcgcggtcccgcaccccgcaccccccaccccaggaacccggcgcggcgcacccc 480
    ----:----|----:----|----:----|----:----|----:----|----:----|
```

```
    R  N  P  A  R  R  T  P  G  T  R  R  G  A  P  Q  E  P  G  A    F1
     G  T  R  R  G  A  P  Q  E  P  G  A  A  H  P  R  N  P  A  R   F2
      E  P  G  A  A  H  P  R  N  P  A  R  R  T  P  G  T  R  R  G  F3
481 aggaacccggcgcggcgcaccccaggaacccggcgcggcgcaccccaggaacccggcgcg 540
    ----:----|----:----|----:----|----:----|----:----|----:----|

    A  H  P  R  N  P  A  R  R  T  P  G  T  R  R  G  A  P  Q  E    F1
     R  T  P  G  T  R  R  G  A  P  Q  E  P  G  A  A  H  P  R  N   F2
      A  P  Q  E  P  G  A  A  H  P  R  N  P  A  R  R  T  P  G  T  F3
541 gcgcaccccaggaacccggcgcggcgcaccccaggaacccggcgcggcgcaccccaggaa 600
    ----:----|----:----|----:----|----:----|----:----|----:----|

    P  G  A  A  H  P  R  N  P  A  R  R  T  P  G  T  R  R  G  A    F1
     P  A  R  R  T  P  G  T  R  R  G  A  P  R  E  P  G  A  A  H   F2
      R  R  G  A  P  Q  E  P  G  A  A  H  P  G  N  P  A  R  R  T  F3
601 cccggcgcggcgcaccccaggaacccggcgcggcgcaccccgggaacccggcgcggcgca 660
    ----:----|----:----|----:----|----:----|----:----|----:----|

    P  R  E  P  G  A  A  H  P  G  N  P  A  R  R  T  P  G  T  R    F1
     P  G  N  P  A  R  R  T  P  G  T  R  R  G  A  P  Q  E  P  G   F2
      P  G  T  R  R  G  A  P  R  E  P  G  A  A  H  P  R  N  P  A  F3
661 ccccgggaacccggcgcggcgcaccccgggaacccggcgcggcgcaccccaggaacccgg 720
    ----:----|----:----|----:----|----:----|----:----|----:----|

    R  G  A  P  Q  E  P  G  A  A  H  P  R  N  P  A  R  R  T  P    F1
     A  A  H  P  R  N  P  A  R  R  T  P  G  T  R  R  G  A  P  Q   F2
      R  R  T  P  G  T  R  R  G  A  P  Q  E  P  G  A  A  H  P  R  F3
721 cgcggcgcaccccaggaacccggcgcggcgcaccccaggaacccggcgcggcgcacccca 780
    ----:----|----:----|----:----|----:----|----:----|----:----|

    G  T  R  R  G  A  P  Q  P  P  P  R  E  P  G  A  L  L  P  G    F1
     E  P  G  A  A  H  P  S  L  L  P  G  N  L  V  P  S  S  P  G   F2
      N  P  A  R  R  T  P  A  S  S  P  G  T  W  C  P  P  P  R  E  F3
781 ggaacccggcgcggcgcaccccagcctcctccccgggaacctggtgccctcctccccggg 840
    ----:----|----:----|----:----|----:----|----:----|----:----|

    N  L  V  P  S  S  P  G  T  W  C  P  P  P  R  E  P  G  A  L    F1
     T  W  C  P  P  P  R  E  P  G  A  L  L  P  G  N  L  V  P  S   F2
      P  G  A  L  L  P  G  N  L  V  P  S  S  P  G  T  W  C  P  P  F3
841 aacctggtgccctcctccccgggaacctggtgccctcctccccgggaacctggtgccctc 900
    ----:----|----:----|----:----|----:----|----:----|----:----|

    L  P  G  N  L  V  P  S  S  P  G  T  W  C  P  P  P  R  E  P    F1
     S  P  G  T  W  C  P  P  P  R  E  P  G  A  L  L  P  G  N  L   F2
      P  R  E  P  G  A  L  L  P  G  N  L  V  P  S  S  P  G  T  W  F3
901 ctccccgggaacctggtgccctcctccccgggaacctggtgccctcctccccgggaacct 960
    ----:----|----:----|----:----|----:----|----:----|----:----|

    G  A  L  L  P  G  N  L  V  P  S  S  P  G  T  W  C  P  P  P    F1
     V  P  S  S  P  G  T  W  C  P  P  P  R  E  P  G  A  L  L  P   F2
      C  P  P  P  R  E  P  G  A  L  L  P  G  N  L  V  P  S  S  P  F3
961 ggtgccctcctccccgggaacctggtgccctcctccccgggaacctggtgccctcctccc 1020
    ----:----|----:----|----:----|----:----|----:----|----:----|
```

```
        R  E  P  G  A  L  L  P  G  N  L  V  P  S  S  P  L  L  Q  S      F1
         G  N  L  V  P  S  S  P  G  T  W  C  P  P  P  P  Y  S  N  P     F2
          G  T  W  C  P  P  P  R  E  P  G  A  L  L  P  L  T  P  I  P    F3
1021  cgggaacctggtgccctcctccccgggaacctggtgccctcctccccttactccaatcc 1080
      ----:----|----:----|----:----|----:----|----:----|----:----|

            Kaposin A>
        Q  C  M  D  R  G  L  T  V  F  V  A  V  H  V  P  D  V  L  L      F1
         N  A  W  I  E  A  *  R  C  L  W  Q  F  M  S  R  M  C  Y  *     F2
          M  H  G  *  R  L  D  G  V  C  G  S  S  C  P  G  C  V  T  K    F3
1081  caatgcatggatagaggcttgacggtgtttgtggcagttcatgtcccggatgtgttacta 1140
      ----:----|----:----|----:----|----:----|----:----|----:----|

        N  G  W  R  W  R  L  G  A  I  P  P  L  V  C  L  L  A  I  S      F1
         M  G  G  A  G  G  L  G  R  Y  H  H  S  F  V  C  W  R  L  V     F2
          W  V  A  L  E  A  W  G  D  T  T  T  R  L  S  V  G  D  *  C    F3
1141  aatgggtggcgctggaggcttggggcgataccaccactcgtttgtctgttggcgattagt 1200
      ----:----|----:----|----:----|----:----|----:----|----:----|

        V  V  P  P  S  G  Q  R  G  P  V  P  F  R  T  R  V  A  T  G      F1
         L  S  P  R  V  A  S  V  A  P  Y  H  S  G  H  E  L  Q  R  A     F2
          C  P  P  E  W  P  A  W  P  R  T  I  Q  D  T  S  C  N  G  R    F3
1201  gttgtccccccgagtggccagcgtggccccgtaccattcaggacacgagttgcaacgggc 1260
      ----:----|----:----|----:----|----:----|----:----|----:----|

                                                PolyA signal
     A  H  *  S  *  R  A  L  P  K  S  V  S  K  I  K  Y  K  N  T      F1
         R  T  E  A  S  V  P  S  R  R  V  S  V  K  *  N  T  K  T  Q     F2
          A  L  K  L  A  C  P  P  E  E  C  Q  *  N  K  I  Q  K  H  N    F3
1261  gcgcactgaagctagcgtgccctcccgaagagtgtcagtaaaataaaatacaaaaacaca 1320
      ----:----|----:----|----:----|----:----|----:----|----:----|
```

ZM124 Kaposin T1.7A transcript and flanking sequences (KT271466: bp 123,911-117,852)

```
                          ORF73|    P3 promoter??>
       R  S  W  G  L  S  T  G  N  D  I  K  A  T  P  L  P  L  F  P     F1
        G  P  G  D  S  P  Q  E  M  T  *  K  P  H  L  S  P  F  F  L    F2
         V  L  G  T  L  H  R  K  *  H  K  S  H  T  S  P  P  F  S  S   F3
     1 aggtcctggggactctccacaggaaatgacataaaagccacacctctccccctttttcct 60
       ----:----|----:----|----:----|----:----|----:----|----:----|

                   TSS T1.7A>                  uORF1>
       P  *  K  P  P  S  P  L  R  T  C  I  W  R  H  G  C  W  C  V     F1
        P  R  S  H  R  R  R  S  A  L  A  F  G  A  M  G  A  G  V  C    F2
         L  E  A  T  V  A  A  P  H  L  H  L  A  P  W  V  L  V  C  V   F3
    61 ccctagaagccaccgtcgccgctccgcacttgcatttggcgccatgggtgctggtgtgtg 120
       ----:----|----:----|----:----|----:----|----:----|----:----|

       C  G  A  V  F  S  R  P  I  Y  L  N  *  T  H  G  Q  R  L  A     F1
        V  G  Q  C  S  H  D  P  S  T  S  T  E  H  T  D  N  G  *  R    F2
         W  G  S  V  L  T  T  H  L  P  Q  L  N  T  R  T  T  A  S  V   F3
   121 tgtggggcagtgttctcacgacccatctacctcaactgaacacacggacaacggctagcg 180
       ----:----|----:----|----:----|----:----|----:----|----:----|

                                                 Potential CUG uORF3>
                          uORF2>    Potential CUG ORF>
       Y  S  R  G  P  A  S  S  M  G  E  P  D  R  A  P  *  N  S  R     F1
        T  L  A  A  Q  R  R  R  W  E  N  L  T  E  H  P  E  T  P  G    F2
         L  S  R  P  S  V  V  D  G  R  T  *  Q  S  T  L  K  L  Q  A   F3
   181 tactctcgcggcccagcgtcgtcgatgggagaacctgacagagcaccctgaaactccagg 240
       ----:----|----:----|----:----|----:----|----:----|----:----|

        Splice site  P4 promoter>              TSS (T1.5A)>
       L  Y  R  K  R  Y  K  D  K  G  R  P  T  P  S  R  I  T  R  A     F1
        S  T  G  N  A  I  K  T  R  G  D  R  P  P  R  A  L  R  A  R    F2
         L  Q  E  T  L  *  R  Q  G  E  T  D  P  L  A  H  Y  A  R  G   F3
   241 ctctacaggaaacgctataaagacaaggggagaccgacccctcgcgcattacgcgcgcg 300
       ----:----|----:----|----:----|----:----|----:----|----:----|

       A  A  R  R  T  D  L  L  D  L  H  V  S  R  S  G  G  T  P  G     F1
        R  H  G  G  R  I  S  W  I  Y  T  Y  R  G  A  V  A  P  Q  E    F2
         G  T  E  D  G  S  L  G  F  T  R  I  E  E  R  W  H  P  R  N   F3
   301 gcggcacggaggacggatctcttggatttacacgtatcgaggagcggtggcaccccagga 360
       ----:----|----:----|----:----|----:----|----:----|----:----|

       T  R  R  G  A  P  Q  E  P  G  S  T  P  E  P  C  E  P  C  S     F1
        P  G  A  A  H  P  R  N  P  V  V  P  P  N  P  A  N  P  A  V    F2
         P  A  R  R  T  P  G  T  R  *  Y  P  R  T  L  R  T  L  Q  Y   F3
   361 acccggcgcggcgcaccccaggaacccggtagtacccccgaaccctgcgaaccctgcagt 420
       ----:----|----:----|----:----|----:----|----:----|----:----|

       T  P  A  R  S  F  P  P  P  H  P  R  N  P  A  R  R  T  P  G     F1
        P  R  R  G  P  S  P  P  R  T  P  G  T  R  R  G  A  P  Q  E    F2
         P  G  A  V  L  P  P  P  A  P  Q  E  P  G  A  A  H  P  R  N   F3
   421 accccggcgcggtccttcccccccccgcaccccaggaacccggcgcggcgcaccccagga 480
       ----:----|----:----|----:----|----:----|----:----|----:----|

       T  R  *  C  P  R  T  L  Q  T  L  Q  Y  P  G  A  V  L  P  R     F1
        P  G  S  A  P  E  P  C  K  P  C  S  T  P  A  R  S  F  P  A    F2
         P  V  V  P  P  N  P  A  N  P  A  V  P  R  R  G  P  S  P  H   F3
```

```
 481 acccggtagtgcccccgaaccctgcaaaccctgcagtaccccggcgcggtccttccccgc 540
     ----:----|----:----|----:----|----:----|----:----|----:----|

     T  P  H  P  A  P  Q  E  P  G  A  A  Q  P  R  N  P  V  V  P    F1
      P  R  T  P  H  P  R  N  P  A  R  R  N  P  G  T  R  *  C  P   F2
       P  A  P  R  T  P  G  T  R  R  G  A  T  Q  E  P  G  S  A  P  F3
 541 accccgcaccccgcaccccaggaacccggcgcggcgcaacccaggaacccggtagtgccc 600
     ----:----|----:----|----:----|----:----|----:----|----:----|

     P  N  P  A  N  P  A  V  P  R  R  G  P  S  P  H  P  A  P  Q    F1
      R  T  L  Q  T  L  Q  Y  P  G  A  V  L  P  R  T  P  H  P  R   F2
       E  P  C  K  P  C  S  T  P  A  R  S  F  P  A  P  R  T  P  G  F3
 601 ccgaaccctgcaaaccctgcagtaccccggcgcggtccttccccgcaccccgcaccccag 660
     ----:----|----:----|----:----|----:----|----:----|----:----|

     E  P  G  A  A  H  P  R  N  P  A  R  R  T  P  G  T  R  R  G    F1
      N  P  A  R  R  T  P  G  T  R  R  G  A  P  Q  E  P  G  A  A   F2
       T  R  R  G  A  P  Q  E  P  G  A  A  H  P  R  N  P  A  R  R  F3
 661 gaacccggcgcggcgcaccccaggaacccggcgcggcgcaccccaggaacccggcgcggc 720
     ----:----|----:----|----:----|----:----|----:----|----:----|

     A  P  Q  E  P  G  A  A  H  P  R  N  P  A  R  R  T  P  G  T    F1
      H  P  R  N  P  A  R  R  T  P  G  T  R  R  G  A  P  Q  E  P   F2
       T  P  G  T  R  R  G  A  P  Q  E  P  G  A  A  H  P  R  N  P  F3
 721 gcaccccaggaacccggcgcggcgcaccccaggaacccggcgcggcgcaccccaggaacc 780
     ----:----|----:----|----:----|----:----|----:----|----:----|

     R  R  G  A  P  Q  P  P  P  R  E  P  G  A  L  L  P  G  N  P    F1
      G  A  A  H  P  S  L  L  P  G  N  L  V  P  S  S  P  G  T  R   F2
       A  R  R  T  P  A  S  S  P  G  T  W  C  P  P  P  R  E  P  G  F3
 781 cggcgcggcgcaccccagcctcctccccgggaacctggtgccctcctccccgggaacccg 840
     ----:----|----:----|----:----|----:----|----:----|----:----|

     V  P  S  S  P  G  T  R  C  P  P  P  R  E  P  G  A  L  L  P    F1
      C  P  P  P  R  E  P  G  A  L  L  P  G  N  P  V  P  S  S  P   F2
       A  L  L  P  G  N  P  V  P  S  S  P  G  T  R  C  P  P  P  R  F3
 841 gtgccctcctccccgggaacccggtgccctcctccccgggaacccggtgccctcctcccc 900
     ----:----|----:----|----:----|----:----|----:----|----:----|

     G  N  P  V  P  S  S  P  G  T  R  F  P  P  P  R  E  P  G  A    F1
      G  T  R  C  P  P  P  R  E  P  G  S  L  L  P  G  N  P  V  P   F2
       E  P  G  A  L  L  P  G  N  P  V  P  S  S  P  G  T  R  C  P  F3
 901 gggaacccggtgccctcctccccgggaacccggttccctcctccccgggaacccggtgcc 960
     ----:----|----:----|----:----|----:----|----:----|----:----|

                                                Kaposin A>
     L  L  P  G  N  P  V  P  S  S  P  L  T  P  I  P  M  H  G  *    F1
      S  S  P  G  T  R  C  P  P  P  P  L  L  Q  S  Q  C  M  D  R   F2
       P  P  R  E  P  G  A  L  L  P  P  Y  S  N  P  N  A  W  I  E  F3
 961 ctcctccccgggaacccggtgccctcctcccccttactccaatcccaatgcatggatag 1020
     ----:----|----:----|----:----|----:----|----:----|----:----|

     R  L  D  G  I  C  G  S  S  C  P  G  C  V  T  K  W  V  A  L    F1
      G  L  T  V  F  V  A  V  H  V  P  D  V  L  L  N  G  W  R  W   F2
       A  *  R  Y  L  W  Q  F  M  S  R  M  C  Y  *  M  G  G  A  G  F3
1021 aggcttgacggtatttgtggcagttcatgtcccggatgtgttactaaatgggtggcgctg 1080
     ----:----|----:----|----:----|----:----|----:----|----:----|
```

```
     E  A  W  G  D  T  T  T  R  L  S  V  G  D  *  C  C  P  P  E     F1
      R  L  G  A  I  P  P  L  V  C  L  L  A  I  S  V  V  P  P  S    F2
       G  L  G  R  Y  H  H  S  F  V  C  W  R  L  V  L  S  P  R  V   F3
1081 gaggcttggggcgataccaccactcgtttgtctgttggcgattagtgttgtccccccgag 1140
     ----:----|----:----|----:----|----:----|----:----|----:----|

     W  P  A  W  P  R  T  V  Q  D  T  S  C  N  G  R  A  L  K  L     F1
      G  Q  R  G  P  V  P  F  R  T  R  V  A  T  G  E  H  *  S  *    F2
       A  S  V  A  P  Y  R  S  G  H  E  L  Q  R  A  S  T  E  A  S   F3
1141 tggccagcgtggccccgtaccgttcaggacacgagttgcaacgggcgagcactgaagcta 1200
     ----:----|----:----|----:----|----:----|----:----|----:----|

                                 PolyA signal
     A  C  P  P  E  E  C  Q  *  N  K  I  Q  K  H  N  H  G  C  T     F1
      R  A  L  P  K  S  V  S  K  I  K  Y  K  N  T  I  T  V  A  P    F2
       V  P  S  R  R  V  S  V  K  *  N  T  K  T  Q  S  R  L  H  Q   F3
1201 gcgtgccctcccgaagagtgtcagtaaaataaaatacaaaaacacaatcacggttgcacc 1260
     ----:----|----:----|----:----|----:----|----:----|----:----|
```

ZM004 Kaposin T1.7A transcript and flanking sequences (KT271453: bp 123,768-117,391)

```
                       ORF73|    P3 promoter??>
     R  S  W  G  L  S  T  G  N  D  I  K  A  T  P  L  P  L  F  P     F1
      G  P  G  D  S  P  Q  E  M  T  *  K  P  H  L  S  P  F  F  L    F2
       V  L  G  T  L  H  R  K  *  H  K  S  H  T  S  P  P  F  S  S   F3
   1 aggtcctggggactctccacaggaaatgacataaaagccacacctctccccctttttcct 60
     ----:----|----:----|----:----|----:----|----:----|----:----|

                 TSS T1.7A>                  uORF1>
     P  *  K  P  P  S  P  L  R  T  C  I  W  R  H  G  C  W  C  V     F1
      P  R  S  H  R  R  R  S  A  L  A  F  G  A  M  G  A  G  V  C    F2
       L  E  A  T  V  A  A  P  H  L  H  L  A  P  W  V  L  V  C  V   F3
  61 ccctagaagccaccgtcgccgctccgcacttgcatttggcgccatgggtgctggtgtgtg 120
     ----:----|----:----|----:----|----:----|----:----|----:----|

     C  G  A  V  F  S  R  P  I  Y  L  N  *  T  H  G  Q  R  L  A     F1
      V  G  Q  C  S  H  D  P  S  T  S  T  E  H  T  D  N  G  *  R    F2
       W  G  S  V  L  T  T  H  L  P  Q  L  N  T  R  T  T  A  S  V   F3
 121 tgtggggcagtgttctcacgacccatctacctcaactgaacacacggacaacggctagcg 180
     ----:----|----:----|----:----|----:----|----:----|----:----|

                                               Potential CUG uORF3>
                         uORF2>     Potential CUG ORF>
     Y  S  R  G  P  A  S  S  M  G  E  P  D  R  A  P  *  N  S  R     F1
      T  L  A  A  Q  R  R  R  W  E  N  L  T  E  H  P  E  T  P  G    F2
       L  S  R  P  S  V  V  D  G  R  T  *  Q  S  T  L  K  L  Q  A   F3
 181 tactctcgcggcccagcgtcgtcgatgggagaacctgacagagcaccctgaaactccagg 240
     ----:----|----:----|----:----|----:----|----:----|----:----|

      Splice site  P4 promoter>               TSS (T1.5A)>
     L  Y  R  K  R  Y  K  E  E  G  R  R  T  P  S  R  I  T  R  A     F1
      S  T  G  N  A  I  K  K  R  G  D  G  L  P  R  A  L  R  A  R    F2
       L  Q  E  T  L  *  R  R  G  E  T  D  S  L  A  H  Y  A  R  G   F3
 241 ctctacaggaaacgctataaagaagaggggagacggactccctcgcgcattacgcgcgcg 300
     ----:----|----:----|----:----|----:----|----:----|----:----|

     A  A  R  R  T  D  L  S  D  L  H  V  S  R  S  G  G  T  P  G     F1
      R  H  G  G  R  I  S  R  I  Y  T  Y  R  G  A  V  A  P  Q  E    F2
       G  T  E  D  G  S  L  G  F  T  R  I  E  E  R  W  H  P  R  N   F3
 301 gcggcacggaggacggatctctcggatttacacgtatcgaggagcggtggcaccccagga 360
     ----:----|----:----|----:----|----:----|----:----|----:----|

     T  R  R  G  A  P  Q  E  P  G  S  T  P  E  P  C  E  P  C  S     F1
      P  G  A  A  H  P  R  N  P  V  V  P  P  N  P  A  N  P  V  V    F2
       P  A  R  R  T  P  G  T  R  *  Y  P  R  T  L  R  T  L  *  Y   F3
 361 acccggcgcggcgcaccccaggaacccggtagtacccccgaaccctgcgaaccctgtagt 420
     ----:----|----:----|----:----|----:----|----:----|----:----|

     T  P  A  R  S  P  P  R  T  P  G  T  R  R  G  A  P  Q  E  P     F1
      P  R  R  G  P  P  P  A  P  Q  E  P  G  A  A  H  P  R  N  P    F2
       P  G  A  V  P  P  P  H  P  R  N  P  A  R  R  T  P  G  T  R   F3
 421 accccggcgcggtcccccccccgcaccccaggaacccggcgcggcgcaccccaggaaccc 480
     ----:----|----:----|----:----|----:----|----:----|----:----|

     G  A  A  H  P  R  N  P  A  R  R  T  P  G  T  R  R  G  A  P     F1
      A  R  R  T  P  G  T  R  R  G  A  P  Q  E  P  G  A  A  H  P    F2
       R  G  A  P  Q  E  P  G  A  A  H  P  R  N  P  A  R  R  T  P   F3
```

```
 481 ggcgcggcgcaccccaggaacccggcgcggcgcaccccaggaacccggcgcggcgcaccc 540
     ----:----|----:----|----:----|----:----|----:----|----:----|

     Q  E  P  G  A  A  H  P  R  N  P  A  R  R  T  P  G  T  R  R     F1
      R  N  P  A  R  R  T  P  G  T  R  R  G  A  P  Q  E  P  G  A    F2
       G  T  R  R  G  A  P  Q  E  P  G  A  A  H  P  R  N  P  A  R   F3
 541 caggaacccggcgcggcgcaccccaggaacccggcgcggcgcaccccaggaacccggcgc 600
     ----:----|----:----|----:----|----:----|----:----|----:----|

     G  A  P  Q  E  P  G  A  X  X  X  X  X  X  X  R  N  P  A  R     F1
      A  H  P  R  N  P  A  R  X  X  X  X  X  X  X  G  T  R  R  G    F2
       R  T  P  G  T  R  R  G  X  X  X  X  X  X  X  E  P  G  A  A   F3
 601 ggcgcaccccaggaacccggcgcggnnnnnnnnnnnnnnnnnnnnaggaacccggcgcgg 660
     ----:----|----:----|----:----|----:----|----:----|----:----|

     R  T  P  G  T  R  R  G  A  P  Q  E  P  G  A  A  H  P  R  N     F1
      A  P  Q  E  P  G  A  A  H  P  R  N  P  A  R  R  T  P  G  T    F2
       H  P  R  N  P  A  R  R  T  P  G  T  R  R  G  A  P  Q  E  P   F3
 661 cgcaccccaggaacccggcgcggcgcaccccaggaacccggcgcggcgcaccccaggaac 720
     ----:----|----:----|----:----|----:----|----:----|----:----|

     P  A  R  R  T  P  G  T  R  R  G  A  P  Q  P  P  P  R  E  P     F1
      R  R  G  A  P  Q  E  P  G  A  A  H  P  S  L  L  P  G  N  L    F2
       G  A  A  H  P  R  N  P  A  R  R  T  P  A  S  S  P  G  T  W   F3
 721 ccggcgcggcgcaccccaggaacccggcgcggcgcaccccagcctcctccccgggaacct 780
     ----:----|----:----|----:----|----:----|----:----|----:----|

     G  A  L  L  P  G  N  L  V  P  S  S  P  G  T  W  C  P  P  P     F1
      V  P  S  S  P  G  T  W  C  P  P  P  R  E  P  G  A  L  L  P    F2
       C  P  P  P  R  E  P  G  A  L  L  P  G  N  L  V  P  S  S  P   F3
 781 ggtgccctcctccccgggaacctggtgccctcctccccgggaacctggtgccctcctccc 840
     ----:----|----:----|----:----|----:----|----:----|----:----|

     R  E  P  G  A  L  L  P  G  N  L  V  P  S  S  P  G  T  W  C     F1
      G  N  L  V  P  S  S  P  G  T  W  C  P  P  P  R  E  P  G  A    F2
       G  T  W  C  P  P  P  R  E  P  G  A  L  L  P  G  N  L  V  P   F3
 841 cgggaacctggtgccctcctccccgggaacctggtgccctcctccccgggaacctggtgc 900
     ----:----|----:----|----:----|----:----|----:----|----:----|

     P  P  P  R  E  P  G  A  L  L  P  G  N  L  V  P  S  S  P  G     F1
      L  L  P  G  N  L  V  P  S  S  P  G  T  W  C  P  P  P  R  E    F2
       S  S  P  G  T  W  C  P  P  P  R  E  P  G  A  L  L  P  G  N   F3
 901 cctcctccccgggaacctggtgccctcctccccgggaacctggtgccctcctccccggga 960
     ----:----|----:----|----:----|----:----|----:----|----:----|

     T  W  C  P  P  P  R  E  P  G  A  L  L  P  G  N  L  V  P  S     F1
      P  G  A  L  L  P  G  N  L  V  P  S  S  P  G  T  W  C  P  P    F2
       L  V  P  S  S  P  G  T  W  C  P  P  P  R  E  P  G  A  L  L   F3
 961 acctggtgccctcctccccgggaacctggtgccctcctccccgggaacctggtgccctcc 1020
     ----:----|----:----|----:----|----:----|----:----|----:----|
                             Kaposin A>
     S  P  L  L  Q  S  Q  C  M  D  R  G  L  T  V  F  V  A  V  H     F1
      P  P  Y  S  N  P  N  A  W  I  E  A  *  R  C  L  W  Q  F  M    F2
       P  L  T  P  I  P  M  H  G  *  R  L  N  G  V  C  G  S  S  C   F3
1021 tccccttactccaatcccaatgcatggatagaggcttaacggtgtttgtggcagttcat 1080
     ----:----|----:----|----:----|----:----|----:----|----:----|

     V  L  D  V  L  L  N  G  W  R  W  R  L  G  A  I  P  P  L  V     F1
```

```
      S  W  M  C  Y  *  M  G  G  A  G  G  L  G  R  Y  H  H  S  F    F2
       P  G  C  V  T  K  W  V  A  L  E  A  W  G  D  T  T  T  R  L   F3
1081 gtcctggatgtgttactaaatgggtggcgctggaggcttggggcgataccaccactcgtt 1140
     ----:----|----:----|----:----|----:----|----:----|----:----|

     C  V  L  A  I  S  V  V  P  P  S  G  Q  R  G  P  V  Q  F  R     F1
      V  C  W  R  L  V  L  S  P  R  V  A  S  V  A  P  Y  N  S  G    F2
       C  V  G  D  *  C  C  P  P  E  W  P  A  W  P  R  T  I  Q  D   F3
1141 tgtgtgttggcgattagtgttgtccccccgagtggccagcgtggccccgtacaattcagg 1200
     ----:----|----:----|----:----|----:----|----:----|----:----|

     T  R  V  A  T  G  A  H  *  S  *  R  A  L  P  R  S  V  S  K     F1
      H  E  L  Q  R  A  R  T  E  A  S  V  P  S  R  G  V  S  V  K    F2
       T  S  C  N  G  R  A  L  K  L  A  C  P  P  E  E  C  Q  *  N   F3
1201 acacgagttgcaacgggcgcgcactgaagctagcgtgccctcccgaggagtgtcagtaaa 1260
     ----:----|----:----|----:----|----:----|----:----|----:----|

     I  K  Y  K  N  T  I  T  V  A  P  S  T  T  S  N  T  Y  N  A     F1
      *  N  T  K  T  Q  S  R  L  H  Q  A  Q  H  Q  T  H  T  M  L    F2
       K  I  Q  K  H  N  H  G  C  T  K  H  N  I  K  H  I  Q  C  *   F3
1261 ataaaatacaaaaacacaatcacggttgcaccaagcacaacatcaaacacatacaatgct 1320
     ----:----|----:----|----:----|----:----|----:----|----:----|

     E  E  Q  A  Y  R  G  H  T  R  G  S  *  H  S  H  S  X           F1
      K  S  R  R  I  E  G  I  P  V  A  H  N  T  V  T  V             F2
       R  A  G  V  S  R  A  Y  P  W  L  I  T  Q  S  Q  X            F3
1321 gaagagcaggcgtatcgagggcatacccgtggctcataacacagtcacagtt 1372
     ----:----|----:----|----:----|----:----|----:----|--
```
