# Supplementary material for: Quantitative RNAseq analysis of Ugandan KS tumors reveals KSHV gene expression dominated by transcription from the LTd downstream latency promoter
Source: PLoS Pathog. 2018 Dec 17;14(12):e1007441. doi: 10.1371/journal.ppat.1007441 (PMC6312348; doi:10.1371/journal.ppat.1007441)
Supplement: S1 Table — (PDF) [file ppat.1007441.s005.pdf]

S1 Table – Summary of KS samples and RNAseq libraries

KS biopsy	Morpho-type	Gen-der	CD4	HIV (log)	RNA Library	Total Reads[1]	KSHV-mapped reads		
							Genome[2]	UCDS[3]	% total[4]
001-B	Macular	M	127	5.44	PE	9.1E+07	40446	30257	74.8
001-C	Macular	M	127	5.44	PE	9.7E+07	134856	100327	74.4
003-C	Macular	M	45	5.88	PE	9.2E+07	3491	2485	71.2
004-D	Nodular	M	85	5.44	PE	1.1E+08	3060	2647	86.5
005-C	Macular	M	199	6.08	PE	9.8E+07	13	11	84.6
006-B	Macular	M	391	5.26	PE	9.9E+07	38605	27181	70.4
006-C	Nodular	M	391	5.26	PE	1.0E+08	18703	14098	75.4
007-B	Macular	M	136	4.96	PE	9.4E+07	2549	2076	81.4
008-B	Nodular	M	422	5.93	PE	1.0E+08	53177	42617	80.1
008-C	Macular	M	422	5.93	PE	1.1E+08	8386	5643	67.3
009-B	Macular	M	175	6.14	PE	9.4E+07	2303	1541	66.9
010-B	Macular	M	25	5.58	PE	9.5E+07	11376	8928	78.5
010-C	Fungating	M	25	5.58	PE	1.1E+08	88	74	84.1
011-C	Nodular	M	8	5.55	PE	1.0E+08	2548	2177	85.4
011-D	Macular	M	8	5.55	PE	1.2E+08	5928	4959	83.7
012-C	Nodular	M	615	5.56	PE	1.0E+08	28	18	64.3
012-D	Macular	M	615	5.56	PE	9.5E+07	62240	43692	70.2
013-B	Macular	M	49	6.02	PE	1.0E+08	104164	83014	79.7
013-C	Nodular	M	49	6.02	PE	1.1E+08	158924	122818	77.3
014-B	Macular	M	11	6.14	PE	9.8E+07	822	719	87.5
015-B	Macular	M	207	5.71	PE	9.7E+07	39173	28344	72.4
018-B	Macular	M	217	5.76	PE	1.2E+08	676	545	80.6
020-B	Nodular	M	370	5.07	PE	1.1E+08	66210	50319	76
022-B	Macular	M	58	5.45	PE	1.2E+08	4348	3051	70.2
023-B	Fungating	F	191	5.53	PE	9.6E+07	146773	126439	86.1
024-B	Nodular	F	416	4.96	PE	1.1E+08	97610	72122	73.9
026-B	Macular	F	56	6.13	PE	1.0E+08	18393	13939	75.8
026-C	Macular	F	56	6.13	PE	1.1E+08	15978	11783	73.7
028-B	Macular	M	221	5.11	PE	8.3E+07	3534	2446	69.2
028-D	Fungating	M	221	5.11	PE	1.1E+08	116	91	78.4
029-B	Nodular	M	5	5.04	PE	9.2E+07	32373	23964	74
029-C	Nodular	M	5	5.04	PE	8.9E+07	9753	7297	74.8
030-B	Nodular	M	70	5	PE	1.0E+08	14930	12082	80.9
030-C	Nodular	M	70	5	PE	1.2E+08	139580	113586	81.4
032-B	Macular	F	274	5.76	PE	8.4E+07	2825	2506	88.7
034-B	Macular	F	237	5.12	PE	8.1E+07	3136	2550	81.3
037-B	Macular	M	574	4.74	PE	8.8E+07	10232	6921	67.6
088-C	Macular	M	102	6.22	ST	4.3E+07	2432	1647	67.7
092-C	Macular	M	50	6.67	ST	3.5E+07	210	187	89
099-C	Macular	M	215	5.50	ST	5.0E+07	73606	47617	64.7
101-C	Fungating	M	331	5.32	ST	5.2E+07	75915	52423	69.1

[1]Total reads in biopsy RNAseq library
[2]Total reads mapping to the complete KSHV GK18 strain reference sequence NC_009333 genome
[3]Reads mapping only to the UCDS features in the novel gene feature file KSHV NC_009333 UCDS ver 020116.GFF
[4]Percentage of reads mapping to the UCDS features compared to the complete genome. KSHV-specific reads not mapping to the set of UCDS features in the GFF file, mapped to regions of the KSHV genome encoding ambiguous overlapping or unknown transcripts.
