# Supplementary material for: Quantitative RNAseq analysis of Ugandan KS tumors reveals KSHV gene expression dominated by transcription from the LTd downstream latency promoter
Source: PLoS Pathog. 2018 Dec 17;14(12):e1007441. doi: 10.1371/journal.ppat.1007441 (PMC6312348; doi:10.1371/journal.ppat.1007441)
Supplement: S2 Table — (PDF) [file ppat.1007441.s006.pdf]

Latency region transcripts:

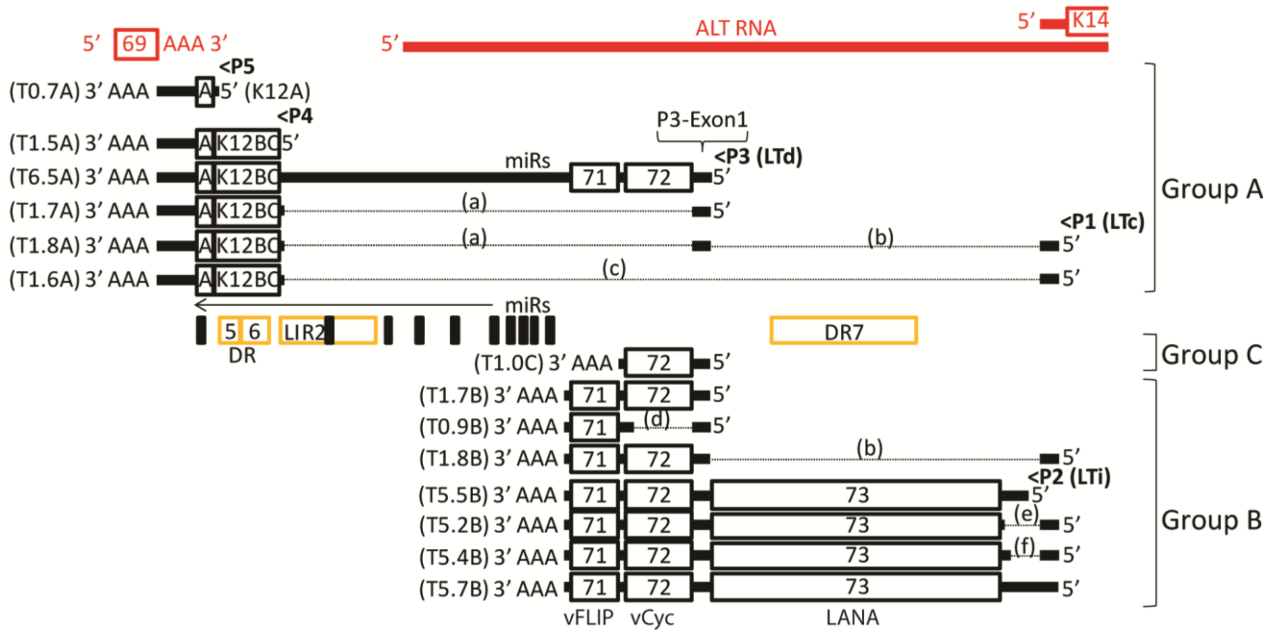

S2 Table: Latency Region Transcripts

mRNA[1]	Coding[2]	Promoter[3]	TSS[4] (bp)	Splice Donor[5] (bp)	Intron[6]	Splice Acceptor[7] (bp)	pA[8] (bp)	Size[9] (Kbp)	Reference[10]
**Group A[11]**									
T0.7A	K12<br>miR-K10,12	P5	118,233	-	-	-	117,553	0.69	[1]
T1.5A	K12A/B/C<br>miR-K10,12	P4	119,007	-	-	-	117,553	1.46	[2]
T1.7A	K12A/B/C<br>miR-K10,12<br>miR-K1-9,11<br>(i)[12]	P3(LTd)	124,009<br>124,090	123,843	“a”	119,047	117,553	1.667<br>1.748	[3, 4]
T6.5A	K12A/B/C<br>miR-K1-12	P3(LTd)	124,009?<br>124,090?	-	-	-	117,553	6.544	Proposed
T1.8A	K12A/B/C<br>miR-K10,12<br>miR-K1-9,11 (i)	P1(LTc)	128,049	127,962<br>123,843	“b”<br>“a”	124,024<br>119,047	117,553	1.771	[5]
T1.6A	K12A/B/C<br>miR-K10,12<br>miR-K1-9,11 (i)	P1(LTc)	128,049	127,962	“c”	119,047	117,553	1.588	[5]
**Group B[13]**									
T1.7B	ORF72/71	P3(LTd)	124,009	-	-	-	122,342	1.672	[3]
T0.9B	ORF71	P3(LTd)	124,009	123,843	“d”	123,107	122,342	0.936	[6]
T1.8B	ORF72/71	P1(LTc)	128,049	127,962	“b”	124,024	122,342	1.774	[7]
T5.5B	ORF73/72/71	P2(LTi)	127,757	-	-	-	122,342	5.453	[8]
T5.4B	ORF73/72/71	P1(LTc)	128,049	127,962	“f”	127,626	122,342	5.376	[7]
T5.2B	ORF73/72/71	P1(LTc)	128,049	127,962	“e”	127,462	122,342	5.212	[9, 10]
T5.7B	ORF73/72/71	P1(LTc)	128,049	-	-	-	122,342	5.712	[7]
**Group C[14]**									
T1.0C	ORF72	P3(LTd)	124,009?<br>124,090?	-	-	-	123,015	1.001<br>1.082	[9]

[1]mRNA transcripts (Fig 5B) derived from the KSHV GK18 reference sequence (NC_009333) are grouped according to the poly-A termination site (A, B or C) and the size is indicated in Kb. Due to heterogeneity in the length of the DR5, 6 and 7 repeat regions, the sizes of these transcripts vary slightly from corresponding transcripts from other KSHV strains. [2]Coding potential for mono-, bi- and poly-cistronic transcripts. [3]Latency-associated promoters: P1(LTc) constitutive latency promoter ~bp 128089; P2(LTi) inducible latency promoter ~bp 127,790; P3(LTd) downstream latency promoter ~bp 124,131; P4 ~bp 119,040; P5 ~bp 118,250. [4]Approximate position of the transcription start site (TSS). [5]Position of the splice donor sites. [6]Intron designation, as indicated in Fig. 5. [7]Position of the splice acceptor sites. [8]pA: polyadenylation signal. [9]Sizes of transcripts are estimated from the putative transcription start and termination sites, based on the GK18 sequence (NC_009333). [10]Original publication describing the transcript. [11]Group A transcripts terminating at pA site bp 117,548. [12](i) designates coding potential of the intron removed from the pre-mRNA. [13]Group B transcripts terminating at pA site bp 122,342. [14]Group C transcripts terminating at pA site bp 123,015.

References

1. Zhong W, Wang H, Herndier B, Ganem D. Restricted expression of Kaposi sarcoma-associated herpesvirus (human herpesvirus 8) genes in Kaposi sarcoma. Proc Natl Acad Sci U S A. 1996;93(13):6641-6.
2. Sadler R, Wu L, Forghani B, Renne R, Zhong W, Herndier B, et al. A complex translational program generates multiple novel proteins from the latently expressed kaposin (K12) locus of Kaposi's sarcoma-associated herpesvirus. J Virol. 1999;73(7):5722-30.
3. Pearce M, Matsumura S, Wilson AC. Transcripts encoding K12, v-FLIP, v-cyclin, and the microRNA cluster of Kaposi's sarcoma-associated herpesvirus originate from a common promoter. J Virol. 2005;79(22):14457-64. doi: 10.1128/JVI.79.22.14457-14464.2005. PubMed PMID: 16254382; PubMed Central PMCID: PMC1280212.
4. Li H, Komatsu T, Dezube BJ, Kaye KM. The Kaposi's sarcoma-associated herpesvirus K12 transcript from a primary effusion lymphoma contains complex repeat elements, is spliced, and initiates from a novel promoter. J Virol. 2002;76(23):11880-8. PubMed PMID: 12414930; PubMed Central PMCID: PMC136876.
5. Cai X, Cullen BR. Transcriptional origin of Kaposi's sarcoma-associated herpesvirus microRNAs. J Virol. 2006;80(5):2234-42. doi: 10.1128/JVI.80.5.2234-2242.2006. PubMed PMID: 16474131; PubMed Central PMCID: PMC1395403.
6. Grundhoff A, Ganem D. Mechanisms governing expression of the v-FLIP gene of Kaposi's sarcoma-associated herpesvirus. J Virol. 2001;75(4):1857-63. doi: 10.1128/JVI.75.4.1857-1863.2001. PubMed PMID: 11160684; PubMed Central PMCID: PMC114095.
7. Dittmer D, Lagunoff M, Renne R, Staskus K, Haase A, Ganem D. A cluster of latently expressed genes in Kaposi's sarcoma-associated herpesvirus. J Virol. 1998;72(10):8309-15.
8. Matsumura S, Fujita Y, Gomez E, Tanese N, Wilson AC. Activation of the Kaposi's sarcoma-associated herpesvirus major latency locus by the lytic switch protein RTA (ORF50). J Virol. 2005;79(13):8493-505. doi: 10.1128/JVI.79.13.8493-8505.2005. PubMed PMID: 15956592; PubMed Central PMCID: PMC1143749.
9. Sarid R, Wiezorek JS, Moore PS, Chang Y. Characterization and cell cycle regulation of the major Kaposi's sarcoma-associated herpesvirus (human herpesvirus 8) latent genes and their promoter. J Virol. 1999;73(2):1438-46.
10. Talbot SJ, Weiss RA, Kellam P, Boshoff C. Transcriptional analysis of human herpesvirus-8 open reading frames 71, 72, 73, K14, and 74 in a primary effusion lymphoma cell line. Virology. 1999;257(1):84-94.
