# Supplementary material for: Quantitative RNAseq analysis of Ugandan KS tumors reveals KSHV gene expression dominated by transcription from the LTd downstream latency promoter
Source: PLoS Pathog. 2018 Dec 17;14(12):e1007441. doi: 10.1371/journal.ppat.1007441 (PMC6312348; doi:10.1371/journal.ppat.1007441)
Supplement: S4 Table — (PDF) [file ppat.1007441.s008.pdf]

S4 Table: Comparison of total and primary transcripts in 33 KS tumors

KSHV UCDS Feature[1]	Total Transcripts[2] (TPM)[3]			Primary Transcripts[4] (TPM)	
	Median[5]	IQ Range[6]		Median	IQ Range
K1	0	0		0	0
ORF4a	125	0		125	0
ORF4b	425	0		425	0
ORF6	347	47		347	47
ORF7	25	0		25	0
ORF8	56	11		20	0
ORF9	185	34		78	16
ORF10	398	150		210	85
ORF11	2436	1373		1506	1088
K2	10138	7085		10094	6988
ORF2	29	0		29	0
K3	273	98		273	98
ORF70	57	0		57	0
K4	1714	322		1714	322
K4.1	481	0		117	0
K4.2A	177	0		66	0
K4.2	0	0		0	0
LIR1	1625	1321		1625	1321
DR1	1373	285		1373	285
DR2	799	152		799	152
OLAP	1628	416		1628	416
K5	4373	1717		4373	1717
K6	343	0		343	0
K7	267	54		267	54
PAN	37413	6571		37413	6571
ORF16	0	0		0	0
ORF17.5	558	104		239	48
ORF17	108	0		108	0
ORF18	71	0		71	0
ORF19	49	0		15	0
ORF20	0	0		0	0
ORF21	18	0		18	0
ORF22	70	0		11	0
ORF23	61	0		28	0
ORF24	39	0		39	0
ORF25	76	14		76	14
ORF26	48	0		9	0
ORF27	178	38		65	0
ORF28	0	0		0	0
ORF29B	24	0		24	0
ORF30	0	0		0	0
ORF31	37	0		0	0
ORF32	113	43		0	0
ORF33	402	42		223	0
ORF29A	47	0		47	0
ORF34	0	0		0	0
ORF35	147	0		125	0
ORF36	497	131		46	0
ORF37	989	143		140	0
ORF38	2153	433		560	67
ORF39	446	76		446	76
ORF40A	94	13		94	13
ORF41	107	0		107	0
ORF42	392	59		171	26
ORF43	91	11		91	11
ORF44	144	74		144	74
ORF45	2490	797		1045	259
ORF46	903	458		138	0
ORF47	599	169		127	0

ORF48	401	122		401	122
ORF49	840	439		840	439
ORF50	1153	513		1153	513
**K8a**	4187	1968		3020	707
**K8.1**	1988	430		1988	430
ORF52	470	88		470	88
ORF53	92	0		92	0
ORF54	297	30		297	30
ORF55	278	0		278	0
ORF56	92	24		92	24
**ORF57**	2773	707		2476	532
vIRF-1	107	30		107	30
vIRF-4	445	146		445	146
vIRF-3	386	238		386	238
vIRF-2	220	127		220	127
**ORF58**	3777	717		404	0
**ORF59**	2330	753		2330	753
ORF60	671	236		223	22
ORF61	328	0		328	0
ORF62	137	0		137	0
ORF63	101	28		101	28
ORF64	115	71		1	0
**ORF65**	1464	842		811	342
ORF66	613	457		71	0
ORF67	490	206		0	0
ORF67A	652	0		652	0
ORF68	438	288		438	288
ORF69	649	468		300	75
**K12A**	166227	150214		166227	150214
**DR5**	314710	223754		314710	223754
**DR6**	68524	44980		68524	44980
**mIR**	3814	2907		3814	2907
**ORF71**	16054	12526		1298	0
**ORF72**	14784	11251		14784	11251
**K12Aa**	134552	109348		134552	109348
ORF73	1704	1133		1704	1133
K14	71	0		71	0
ORF74	416	208		275	77
**ORF75**	24555	17559		8355	4974
**K15**	15557	9796		15557	9796

[1]The UCDS features used for mapping reads are indicated in genomic order top (left end of genome) to bottom (right end of genome). UCDS features highlighted in red correspond to the highly expressed transcripts in Figure 11.
[2]Total transcripts are determined by quantitation of the reads mapping to each UCDS feature and are normalized to the size of the feature and the total number of KSHV mapped reads in each sample.
[3]TPM = transcripts per million KSHV-mapped reads
[4]Primary transcripts are those transcripts derived from the promoter immediately upstream of each ORF and are determined by subtracting the transcripts for the immediately adjacent upstream ORF in bicistronic or polycistronic loci, as described in Table 3 ([1]).
[5]Median transcript levels across the 33 KS tumors with total KSHV-mapped reads greater than 1,000 – 008B was excluded
[6]Interquartile range of transcripts
[7]Transcripts highlighted in yellow indicate cases in which the primary transcript levels are lower than the levels of total transcripts mapping to that UCDS feature due to the presence of overlapping bicistronic or polycistronic transcripts.

1. Bruce AG, Barcy S, DiMaio T, Gan E, Garrigues HJ, Lagunoff M, et al. Quantitative Analysis of the KSHV Transcriptome Following Primary Infection of Blood and Lymphatic Endothelial Cells. Pathogens. 2017;6(1). doi: 10.3390/pathogens6010011. PubMed PMID: 28335496; PubMed Central PMCID: PMC5371899.
